# Synthesis and Photochemistry of Tris(trimethoxysilyl)acyl-silanes and 1,4-Tetrakis(silyl)-1,4-bisacylsilanes

**DOI:** 10.1021/acs.organomet.3c00531

**Published:** 2024-02-19

**Authors:** Thomas Lainer, Sabrina D. Pueschmann, Ana Torvisco, Roland C. Fischer, Michaela Flock, Michael Haas

**Affiliations:** Institute of Inorganic Chemistry, Graz University of Technology, Stremayrgasse 9/V, 8010 Graz, Austria

## Abstract

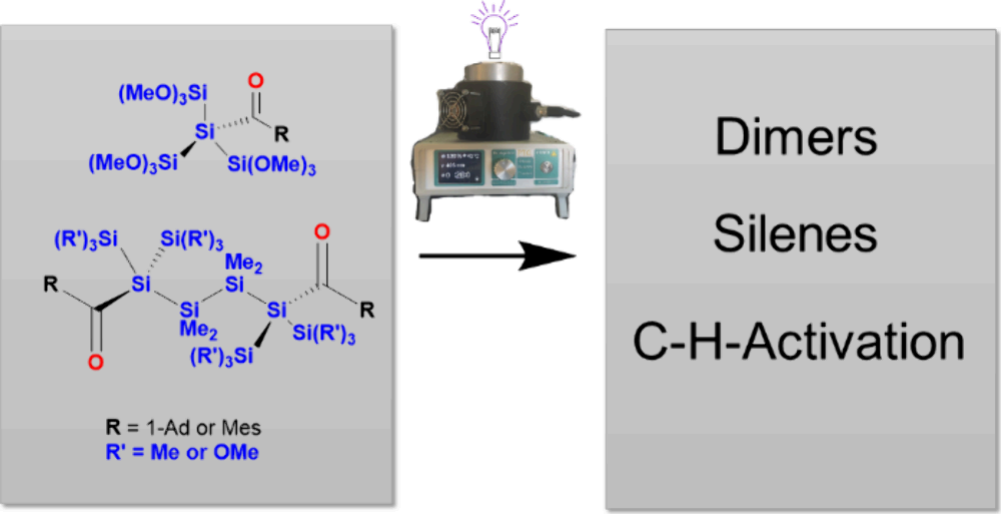

In this contribution, we present the synthesis of two groups of novel acylsilanes **1**–**6**. Compounds **1** and **2** represent tris(trimethoxysilyl)acylsilanes, and compounds **3**–**6** are 1,4-tetrakis(silyl)-1,4-bisacylsilanes. All isolated compounds were characterized by infrared (IR) and nuclear magnetic resonance (NMR) spectroscopy and X-ray crystallography. Additionally, these compounds were further analyzed by ultraviolet/visible (UV/vis) spectroscopy and their longest wavelength absorption bands were assigned by density functional theory (DFT) calculations. On the basis of the well-known Brook rearrangement of acylsilanes, we irradiated **1**–**6** in benzene solutions at 405 nm (λ) for several hours. Photolysis of compounds **1** and **2** afforded the same silene rearrangement products as found in previous investigations of structurally related acylsilanes. In addition, trapping experiments with MeOH further support our proposed mechanism for silene formation. The photolysis of tetrakis(trimethylsilyl)bisacylsilane **3** gave rise to the formation of a monosilene intermediate **10**; upon prolonged irradiation, the subsequently formed bissilene undergoes a fast dimerization to bicyclic product **11**. Interestingly, unlike the expected head-to-head dimerization of Brook-type silenes, this bissilene undergoes a selective head-to-tail dimerization. In contrast, tetrakis(trimethylsilyl)bisacylsilane **4** undergoes a selective and completely stereoselective double CH activation to air stable bicyclic system **12**. The mechanism of this rearrangement is fully described by DTF calculations. Unfortunately, tetrakis(trimethoxysilyl)bisacylsilanes **5** and **6** underwent unselective photochemical rearrangements.

## Introduction

**Scheme 1:**
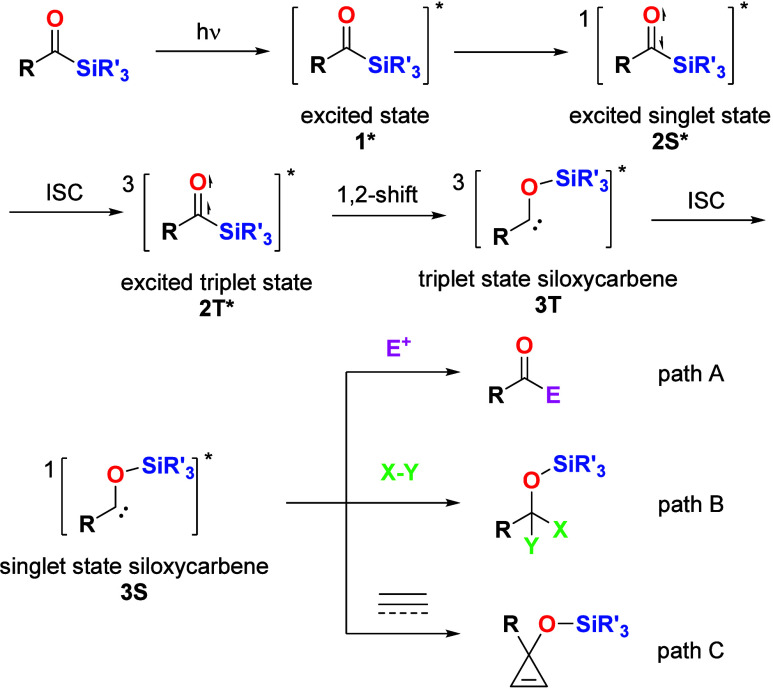
Mechanism for Siloxycarbene Formation and Representative Reactions

Recently, acylsilanes are the subject of intensive research by several branches of chemistry.^1−8^ In particular with the increasing interest in photochemistry, photochemically induced rearrangements of acylsilanes were revisited.^9−11^ The pioneer of this research field was A. G. Brook, who discovered and elaborated the photochemistry of acylsilanes.^12^ He showed that these compounds undergo a thermally induced or a photoinduced 1,2-shift of the silyl group forming siloxycarbenes as labile intermediates. The accepted mechanism for the formation of siloxycarbenes in the singlet state is shown in Scheme 1. Upon irradiation, the acylsilane system is promoted to excited state 1*. On the basis of the small energy gap between the nonbonding and antibonding orbitals of carbonyl compounds, the excited singlet state of acylsilane 2S* is formed by relaxation. An intersystem crossing (ISC) enables the formation of an excited triplet state of acylsilane 2T*, which is followed by 1,2-Brook rearrangement, leading to the corresponding triplet siloxycarbene intermediate **3T**. Subsequently, **3T** undergoes another ISC to form the singlet state of siloxycarbene **3S**.^13^ These carbenes have shown the ability to react as reagents in a variety of organic reactions. The nucleophilic addition of siloxycarbenes to various electrophiles (E^+^) has been used by many groups (Scheme 1, path A). In addition, such carbenes can insert into a large variety of C–H, B–H, or C–B bonds (Scheme 1, path B). Their addition to alkenes and alkynes has also been published (Scheme 1, path C).^14,15^

**Scheme 2:**
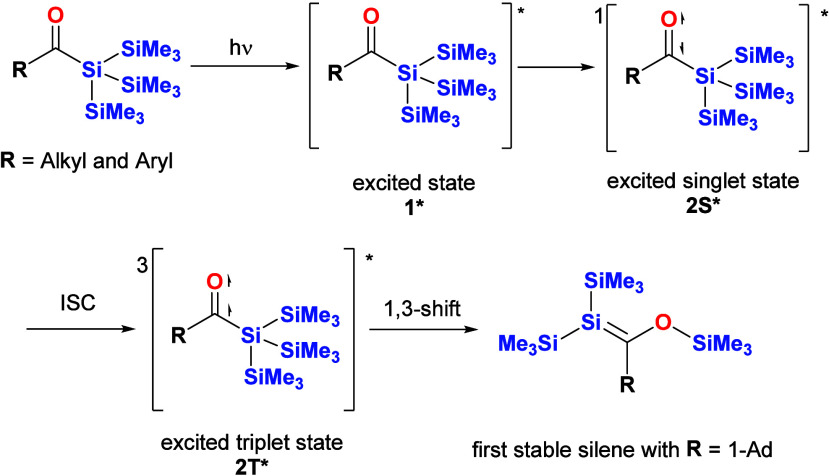
Mechanism for the Formation of Silenes

In the presence of Si–Si bonds in the backbone of the molecule, a photochemically induced 1,3-trimethlysilyl shift is possible. This “unusual” Brook rearrangement was exploited by Brook et al. and led to the isolation of the first stable silene (see Scheme 2).^16^ This was a milestone in organosilicon chemistry and tremendously boosted the number of papers on silene chemistry.

While Brook used a variety of different substituents at the carbonyl moiety, he did not change the substituents at the silyl groups. The implementation of KO^*t*^Bu as a standard reagent for the synthesis of polysilanes opened the door for new molecules.^17^ By a slight modification of the substituent pattern, we found the formation of exocylic^18^ as well as endocyclic silenes.^19,20^ Recently, we introduced a pathway toward tris(trimethoxysilyl)silanides as a new building block for polysilane chemistry.^19,21^ Moreover, we could also show that the nucleophilicity of the central Si atoms is significantly higher than that of structurally related silanides. The reason for this is the negative hyperconjugation induced by the three trimethoxysilyl groups. We therefore asked ourselves whether these groups can also influence the photochemistry of acylsilanes. Consequently, we synthesized novel tris(trimethoxy)acylsilanes and investigated their photochemistry. The second part of this work is devoted to α,ω-bis(acyl)polysilanes. Here, we report on their synthesis and their photochemistry.

## Results and Discussion

### Synthesis of Precursor Molecules

#### Tris(trimethoxysilyl)acylsilanes

**Scheme 3:**
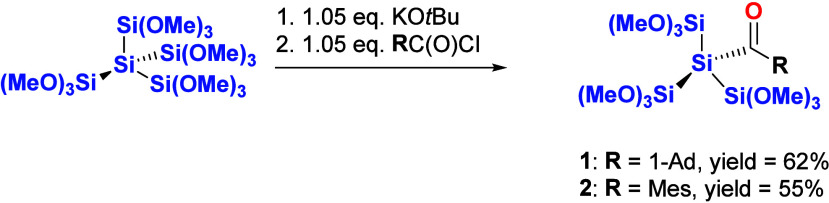
Synthesis of Acylsilanes **1** and **2**

Dodecamethoxyneopentasilane, which was synthesized according to the literature,^22^ was treated with equimolar amounts of potassium *tert*-butoxide (KO^*t*^Bu) in dry tetrahydrofuran (THF) to generate the silanide. Afterward, the anion is reacted with 1 equiv of the corresponding acid chloride (see Scheme 3). The reaction results in the clean formation of acylsilanes **1** and **2** in good yields. Analytical data are consistent with the proposed structures, exhibiting one resonance line in the ^29^Si NMR spectra for the three trimethoxysilyl groups near −42 ppm and one signal for the Si atom bearing the acyl group near −107 ppm for **1** and −98 ppm for **2**. In the ^13^C NMR spectrum, the signal at approximately δ = 242 ppm is characteristic of a carbonyl group bound to silicon.

#### Tetrakis(silyl)-1,4-bisacylsilanes

**Scheme 4:**
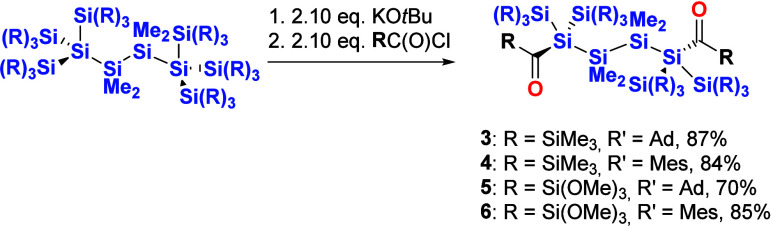
Synthesis of Acylsilanes **3–6**

Hexakis(trimethylsilyl)tetramethyltetrasilane and hexakis(trimethoxysilyl)tetramethyltetrasilane, which were synthesized according to the literature,^23,24^ were treated with 2.10 equiv of KO^*t*^Bu in dry DME and THF, respectively, to generate the dianionic species. Afterward, the dianions are reacted with 2.10 equiv of the corresponding acid chlorides (R = Ad and Mes). After recrystallization, products **3**–**6** can be isolated in high yields (see Scheme 4). Analytical data for **3** and **4** are consistent with the proposed structures, exhibiting one resonance line in the ^29^Si NMR spectra for the four trimethylsilyl groups near δ = −11 ppm, one signal for the dimethylsilyl groups near δ = −30 ppm, and one signal for the Si atom bearing the acyl group at approximately δ = −70 ppm. For compounds **5** and **6**, the ^29^Si NMR spectra show one signal for the four trimethoxysilyl groups near δ = −41 ppm, one signal for the dimethylsilyl groups near δ = −31 ppm, and one signal for the Si atom bearing the acyl group at δ = −93 ppm for compound **5** and at δ = −84 ppm for compound **6**. Again, in the ^13^C NMR spectrum, peaks for the carbonyl groups are observed at approximately δ = 245 ppm.

**Figure 1:**
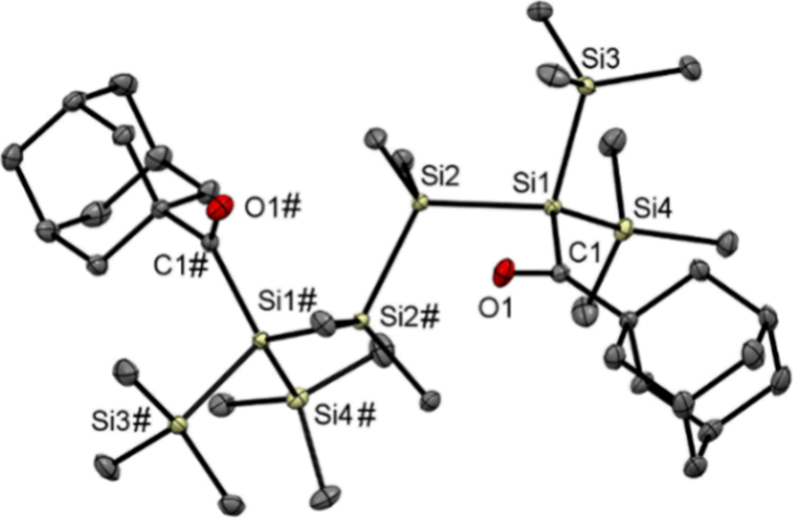
ORTEP representation for compound **3**. Thermal ellipsoids are depicted at the 50% probability level. Hydrogen atoms have been omitted for the sake of clarity. Selected bond lengths (angstroms) with estimated standard deviations: C1–O1, 1.2233(15); Si1–C1, 1.9607(12); Si1–Si2, 2.3750(4); Si1–Si3, 2.3634(5); Si1–Si4, 2.3655(5); Si2–Si2#, 2.3634(6).

**Figure 2:**
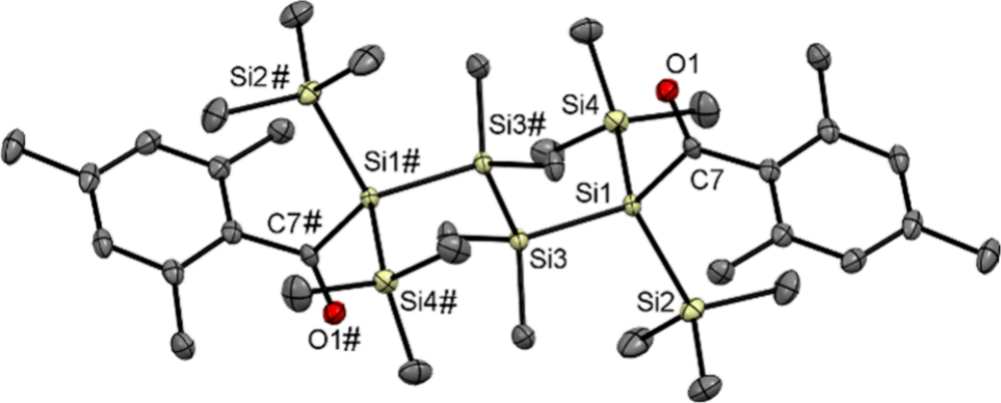
ORTEP representation for compound **4**. Thermal ellipsoids are depicted at the 50% probability level. Hydrogen atoms have been omitted for the sake of clarity. Selected bond lengths (angstroms) with estimated standard deviations: C1–O1, 1.2233(15); Si1–C1, 1.9607(12); Si1–Si2, 2.3750(4); Si1–Si3, 2.3634(5); Si1–Si4, 2.3655(5); Si2–Si2#, 2.3634(6).

We were able to grow crystals suitable for single-crystal X-ray structure analysis by cooling a solution of **3** and **4** in acetone to −30 °C (Figures 1 and 2). Compound **3** crystallizes in tetragonal space group *P*4_3_2_1_2 with four molecules per unit cell. Compound **4** crystallizes in monoclinic space group *P*2_1_/*c* with two molecules per unit cell.

### Spectroscopy

**Figure 3:**
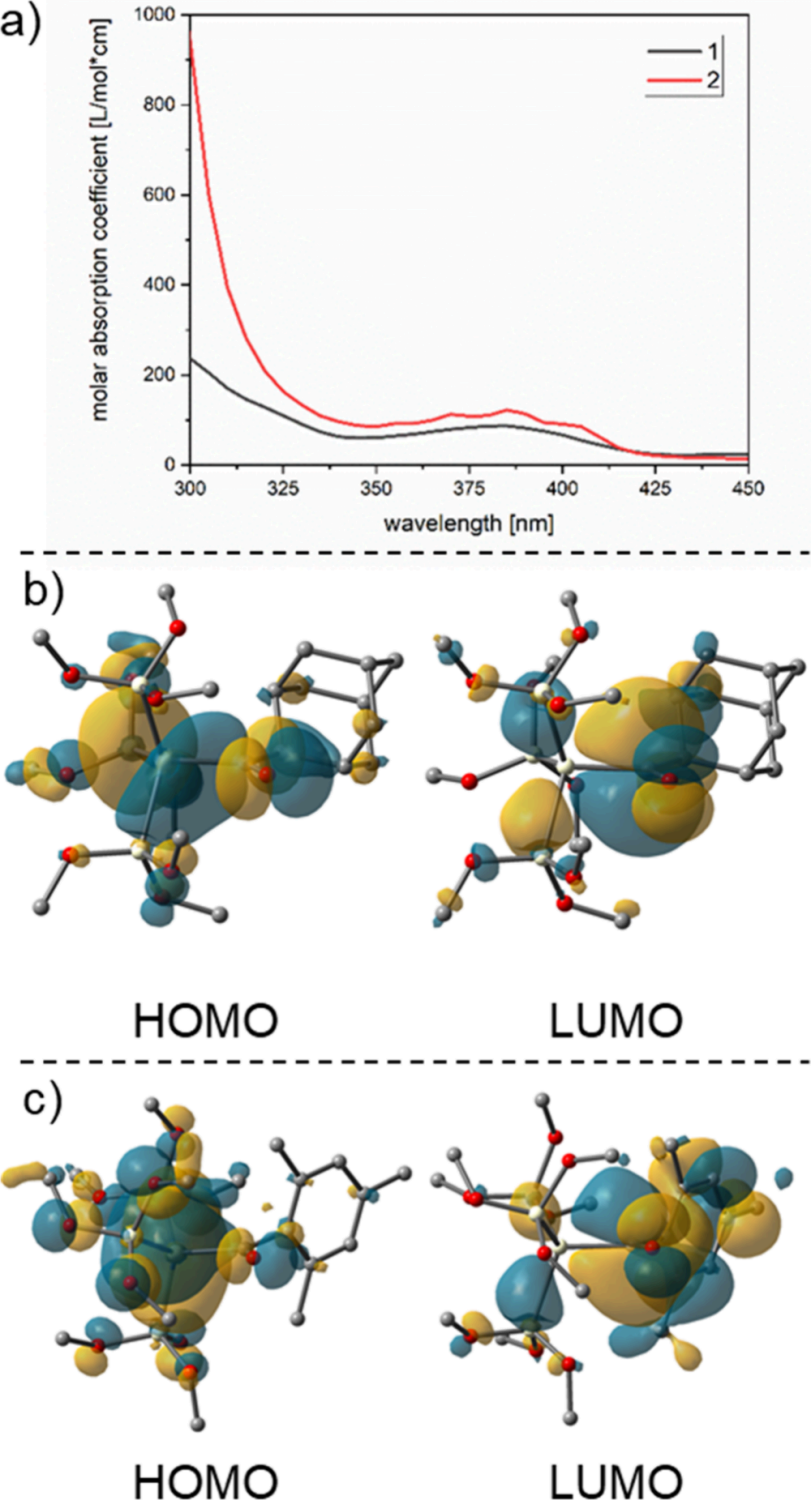
(a) UV/vis spectra of **1** and **2**. Experimental spectra in *n*-hexane at 1 × 10^–3^ mol L^–1^. (b) Orbitals involved in the first transition for the global minimum of compound **1** (with a contour value of 0.02 au). (c) Orbitals involved in the first transition for compound **2** (with a contour value of 0.02 au).

**Figure 4:**
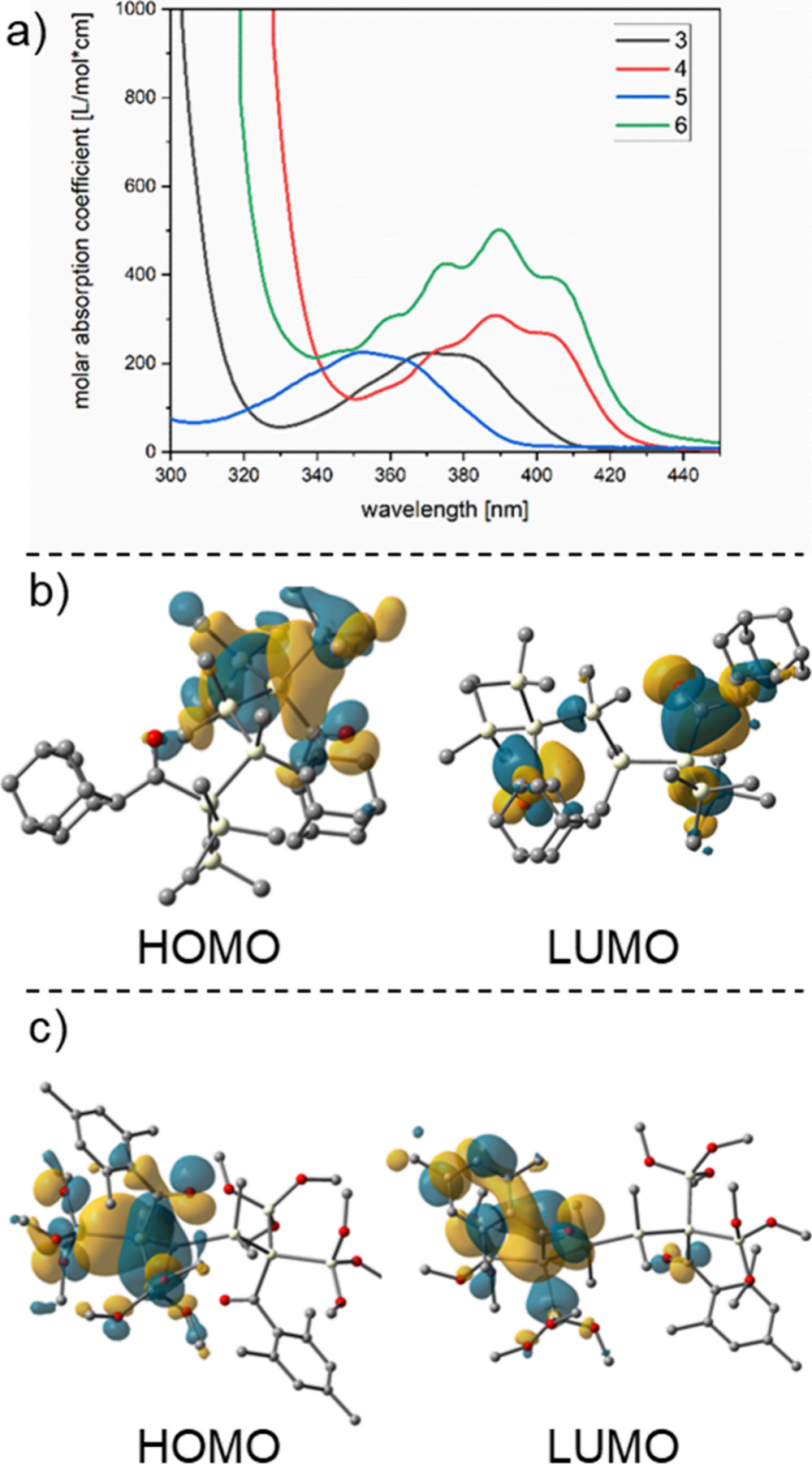
(a) UV/vis spectra of **3**–**6**. Experimental spectra in *n*-hexane at 1 × 10^–3^ mol L^–1^. (b) Orbitals involved in the first transition for compound **3** (with a contour value of 0.02 au). (c) Orbitals involved in the first transition for compound **6** (with a contour value of 0.02 au). Orbital pictures for the other compounds can be found in Figure S42.

To accurately determine the transition for the following photochemical experiments, UV/vis spectra of **1**–**6** were recorded. The UV/vis spectra of **1**–**6** reveal characteristic bands centered between 350 and 410 nm for the adamantoyl-substituted silanes and between 388 and 425 nm for the mesitoyl-substituted silanes (compare Figures 3 and 4). On the basis of time-dependent density functional theory (TDDFT) calculations, all of these transitions can be straightforwardly attributed to nσ → π* transitions. Consequently, we performed irradiations with light-emitting diodes (LEDs) having emission maxima centered at λ = 405 nm (≈295 kJ mol^–1^) for all compounds to selectively address these transitions.

### Photochemistry of **1–6**

#### Tris(trimethoxysilyl)acylsilanes

**Scheme 5:**
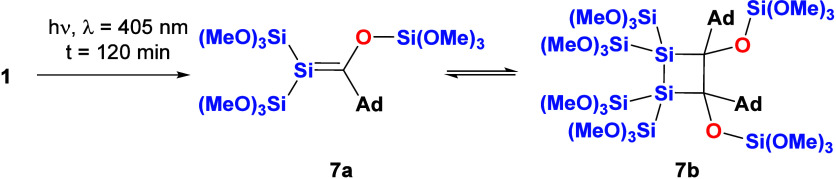
Photolysis of **1**

First, we investigated the photochemistry of tris(trimethoxysilyl)acylsilanes **1** and **2**. On the basis of our spectroscopic investigation, we performed the irradiation experiments at λ = 405 nm in benzene and in the absence of air and moisture. Photolysis of compounds **1** and **2** afforded the same silene rearrangement products as found in previous investigations of structurally related acylsilanes.^25,26^ In the case of **1**, we were able to observe the formation of silene **7a** and the corresponding dimerization product **7b** by performing NMR spectroscopy after irradiation for only 5 min. Upon prolonged irradiation, the staring material was completely consumed after 120 min and silene **7a** and dimer **7b** were obtained in a ratio of ∼1:1 with small amounts of unidentified compounds (see Scheme 5).

**Figure 5:**
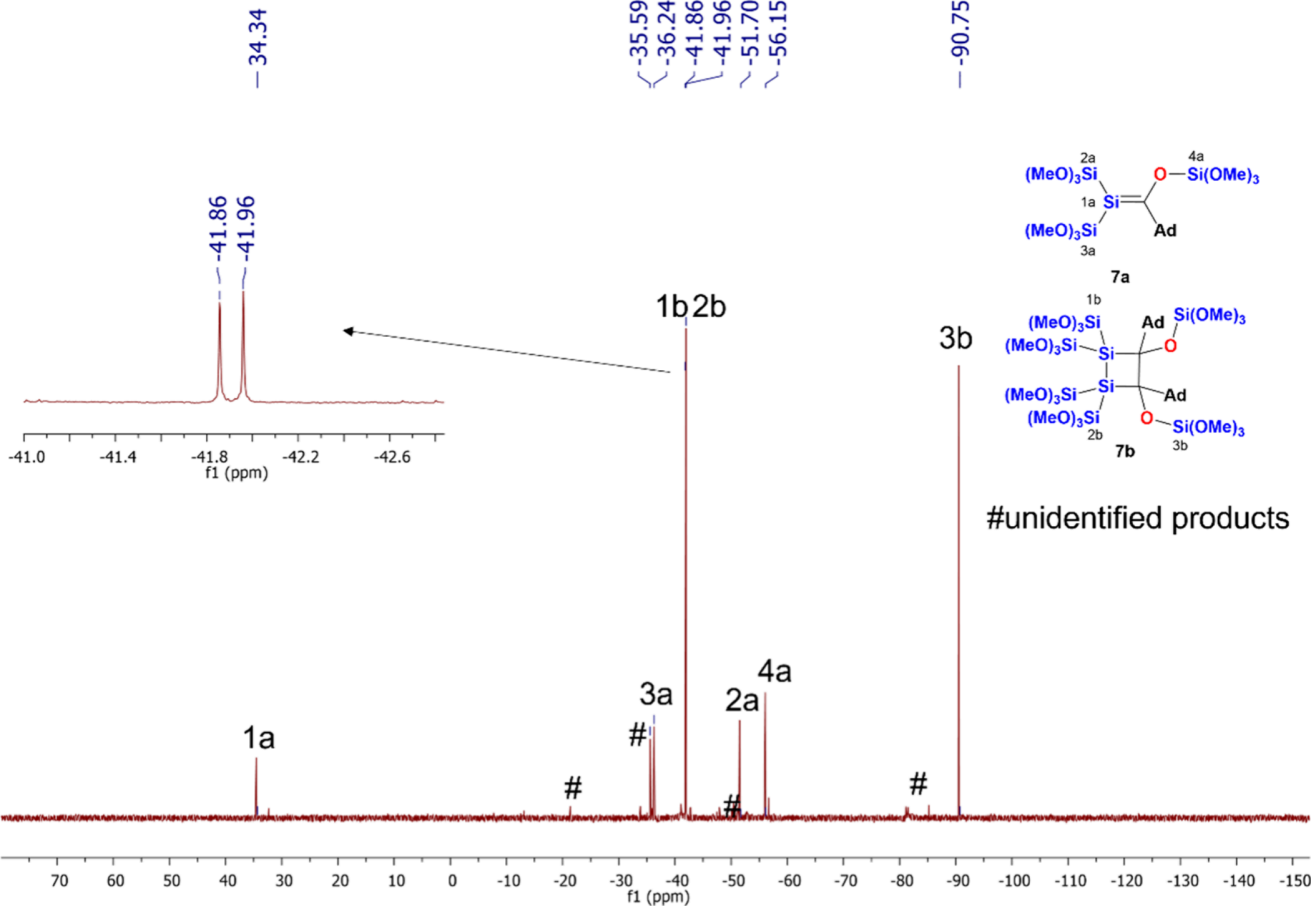
^29^Si NMR spectrum after irradiation at λ = 405 nm for 120 min, including the assignment of the observed resonance lines for **7a** and **7b**.

**Figure 6:**
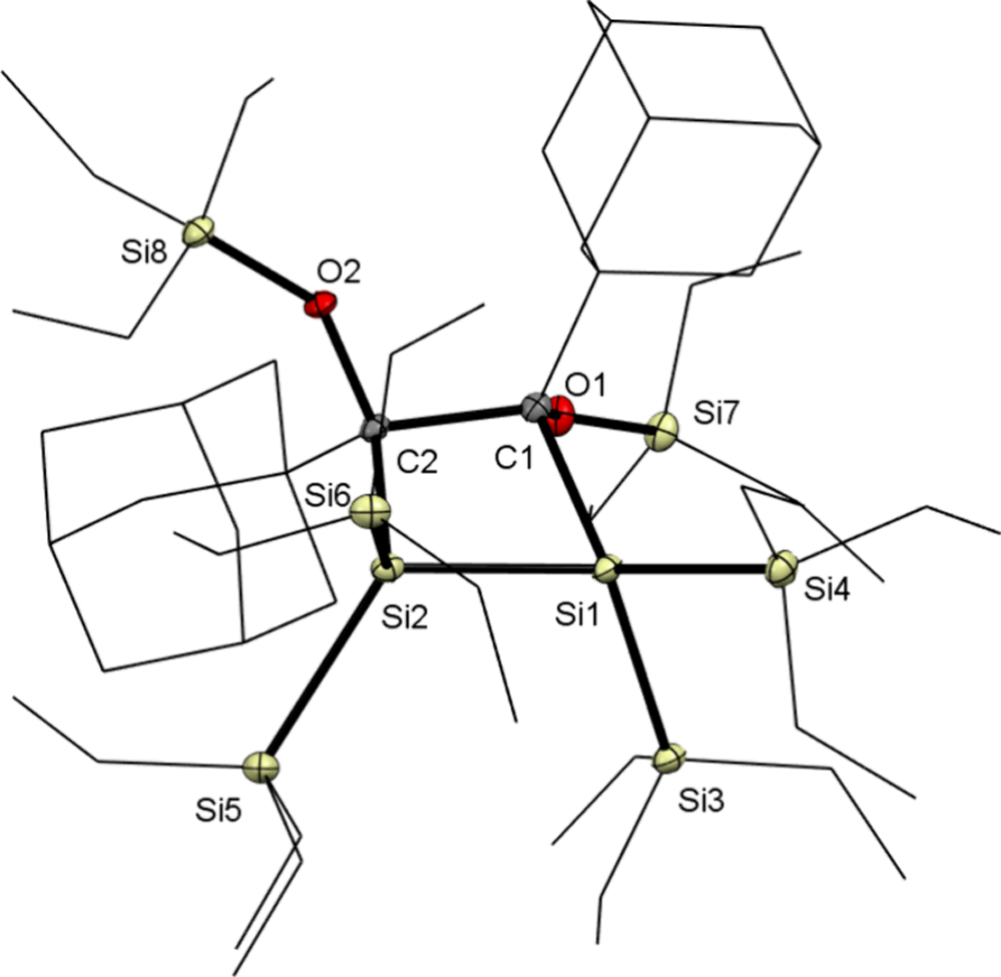
ORTEP representation for compound **7b**. Thermal ellipsoids are depicted at the 50% probability level. Hydrogen atoms have been omitted for the sake of clarity, Moreover, adamantyl (Ad) groups and methoxy groups are shown as wireframes for the sake of clarity. Selected bond lengths (angstroms) and angles (degrees) with estimated standard deviations: C1–O1, 1.4397(13); C1–C2, 1.6615(16); Si1–C1, 2.0088(12); Si1–Si2, 2.3430(5); Si1–Si3, 2.3677(4); Si2–C2, 2.0153(12); C2–O2, 1.4380(13); O1–Si7, 1.5945(9); C2–C1–Si, 1 98.90(7); C1–Si1–Si2, 80.02(3).

Further irradiation does not change the ratio of the products but afforded increased amounts of unidentified polymeric decomposition products at the expense of **7a** and **7b** (see Figure 23). Figure 5 shows the ^29^Si NMR spectrum after irradiation for 120 min, including the assignment of the observed resonance lines (all other obtained spectroscopic data that strongly support the structural assignment are shown in the Experimental Section, together with experimental details). Unfortunately, all attempts to separate both compounds failed, but crystals of sufficient quality for X-ray analysis for dimer **7b** were obtained by cooling a concentrated solution of the mixture in *n*-pentane to −30 °C (see Figure 6). As for all other structurally related Brook-type silenes, head-to-head dimerization was observed.

As outlined in Figure 5, the ^29^Si NMR spectrum depicts several well-separated shifts, which can be easily assigned to the two corresponding products. For silene **7a**, the characteristic signal for the Si=C fragment was observed at δ = 34.3 ppm, which is shifted significantly upfield to the corresponding trimethylsilyl-substituted silene isolated by Brook et al.^26^ Furthermore, the signals at δ = −36.2 ppm and δ = −51.70 ppm can be assigned to the Si(OMe)_3_ groups. Finally, the OSi(OMe)_3_ moiety appeared at δ = −56.2 ppm, which is shifted strongly downfield to other structurally related OSi(OMe)_3_ groups (see compound **7b**). We assume the ylidic resonance structure is responsible for this shift. For dimer **7b**, the Si(OMe)_3_ signals were assigned at δ = −41.9 ppm and δ = −42.0 ppm. The signal for the OSi(OMe)_3_ appeared at δ = −90.8 ppm. Previous experience with structurally related compounds allowed the full assignment of the ^29^Si NMR spectrum.^21^ The ^13^C NMR spectrum showed the characteristic shift for the Si=C fragment at δ = 172.9 ppm, which is also shifted strongly upfield to the corresponding trimethylsilyl-substituted silene isolated by Brook et al.^26^ Again, we assume that the ylidic resonance structure is responsible for this shift.

**Scheme 6:**
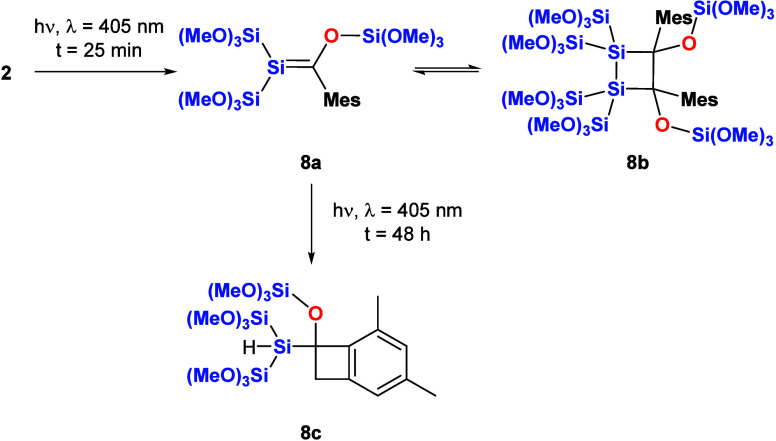
Photolysis of **2**

Again, the photolysis of mesityl-substituted acylsilane **2** was performed at λ = 405 nm in benzene and in the absence of air moisture. After irradiation for 25 min, the starting material was completely consumed. The performed NMR analysis at this stage showed the formation of three products. Again, the resonance lines characteristic for silene **8a** and its dimer, **8b**, were determined. In contrast to **1**, the ratio of this equilibrium is strongly shifted to the dimeric form (∼1:0.05). In addition, silene **8a** is photochemically unstable and underwent an intramolecular C–H bond addition reaction of one *o*-CH_3_ groups at the aromatic ring under formation of spirocyclic compound **8c** (see Scheme 6). This C–H bond addition reaction is a well-known reactivity of mesityl-substituted Brook-type silenes.^20,25^

**Figure 7:**
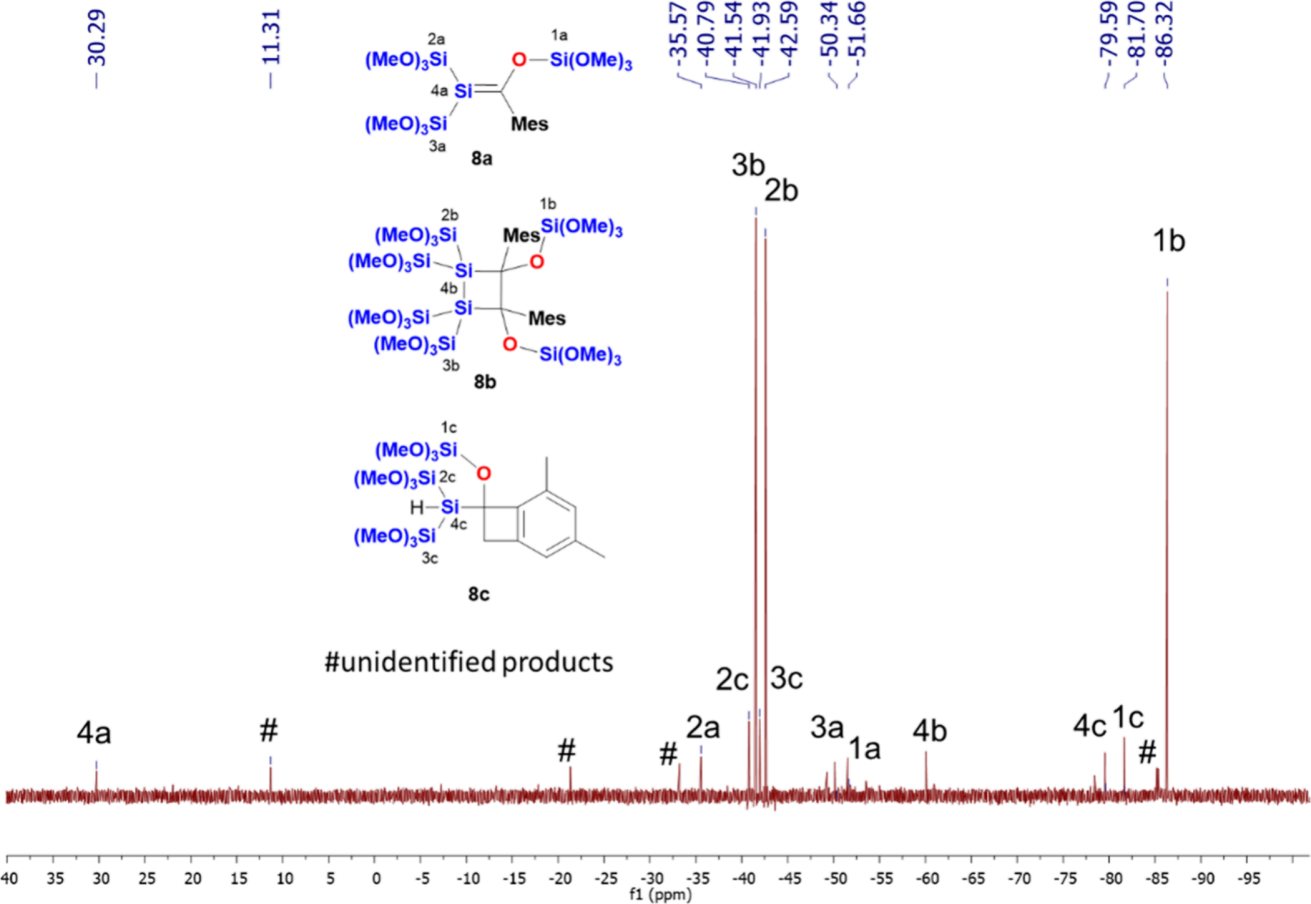
^29^Si NMR spectrum after the irradiation at λ = 405 nm for 25 min, including the assignment of the observed resonance lines for **8a**–**c**.

Figure 7 shows the ^29^Si NMR spectrum after the irradiation for 25 min, including the assignment of the observed resonance lines (all other obtained spectroscopic data that strongly support the structural assignment are shown in the Experimental Section, together with experimental details).

Again, for silene **8a**, the characteristic signal for the Si=C fragment was observed at δ = 30.3 ppm, and similar shifts for structurally related compounds are found in the same region.^18^ As all other shifts for the silene and the corresponding dimer are found in the same region as those for compound **7a** and **7b**, we summarize them in Table 1.

**Table 1:** Summarized ^29^Si NMR Shifts for **7a**, **7b**, **8a**, and **8b**

	**7a**	**7b**	**8a**	**8b**
Si=C	34.3		30.3	
Si(OMe)_3_	–36.2	–41.9	–36.6	–41.6
	–51.7	–42.0	–50.3	–42.6
OSi(OMe)_3_	–56.2	–90.8	–51.6	–86.3
Si–C		–73.0		–60.1

Upon prolonged irradiation, the signals for **8a** and **8b** slowly vanished, leading to the formation of C–H activation product **8c** as the main product. Unfortunately, all attempts to separate **8c** from small amounts of uncharacterizable side products failed. However, all other obtained spectroscopic data that strongly support the structural assignment are given in the Experimental Section, together with experimental details.

**Scheme 7:**
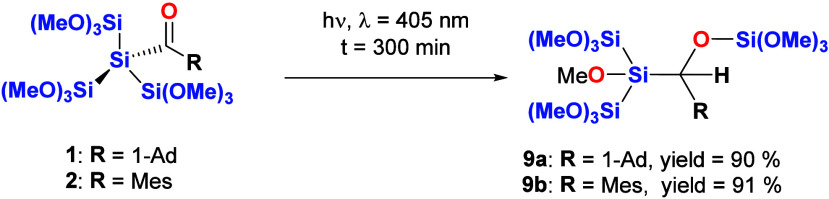
Photolysis of **1** and **2** in the Presence of MeOH

Our proposed mechanism for silene formation was supported by trapping experiments with MeOH in the presence of Et_3_N. For both acylsilanes, the expected 1,2-MeOH addition products of the silenes were obtained nearly quantitatively (see Scheme 7). Analytical and spectroscopic data that strongly support the structural assignment are summarized in the Experimental Section, together with experimental details.

#### Tetrakis(trimethylsilyl)-1,4-bisacylsilanes

**Scheme 8:**
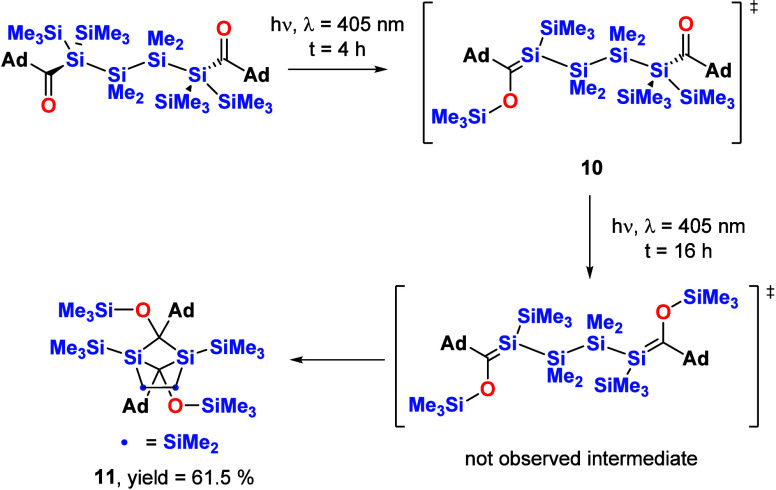
Photolysis of **3**

On the basis of our spectroscopic investigation, we performed the irradiation experiments at λ = 405 nm with compounds **3** and **4** in benzene and in the absence of air and moisture. In the case of **3**, we were able to observe the formation of a monosilene intermediate **10** and small amounts of dimerization product **11** by performing ^29^Si NMR spectroscopy after irradiation for 4 h (see Figure 10). These deeply yellow intermediate showed the characteristic Si=C shift at δ = 41.6 ppm, whereas the OSiMe_3_ signal can be found at δ = 13.2 ppm. Upon prolonged irradiation, the staring material and the silene intermediate were completely consumed and dimer **11** was obtained alongside with minor amounts of an uncharacterizable polymer (see Scheme 8). The end product showed a high photochemical stability with unchanged NMR spectra over a prolonged period of time. Compound **11** was isolated by crystallization from *n*-pentane at −30 °C. Analytical and spectroscopic data that strongly support the structural assignment are summarized in the Experimental Section, together with experimental details.

Interestingly, in contrast to the expected head-to-head dimerization of Brook-type silenes, this bissilene undergoes a selective head-to-tail dimerization.

**Figure 8:**
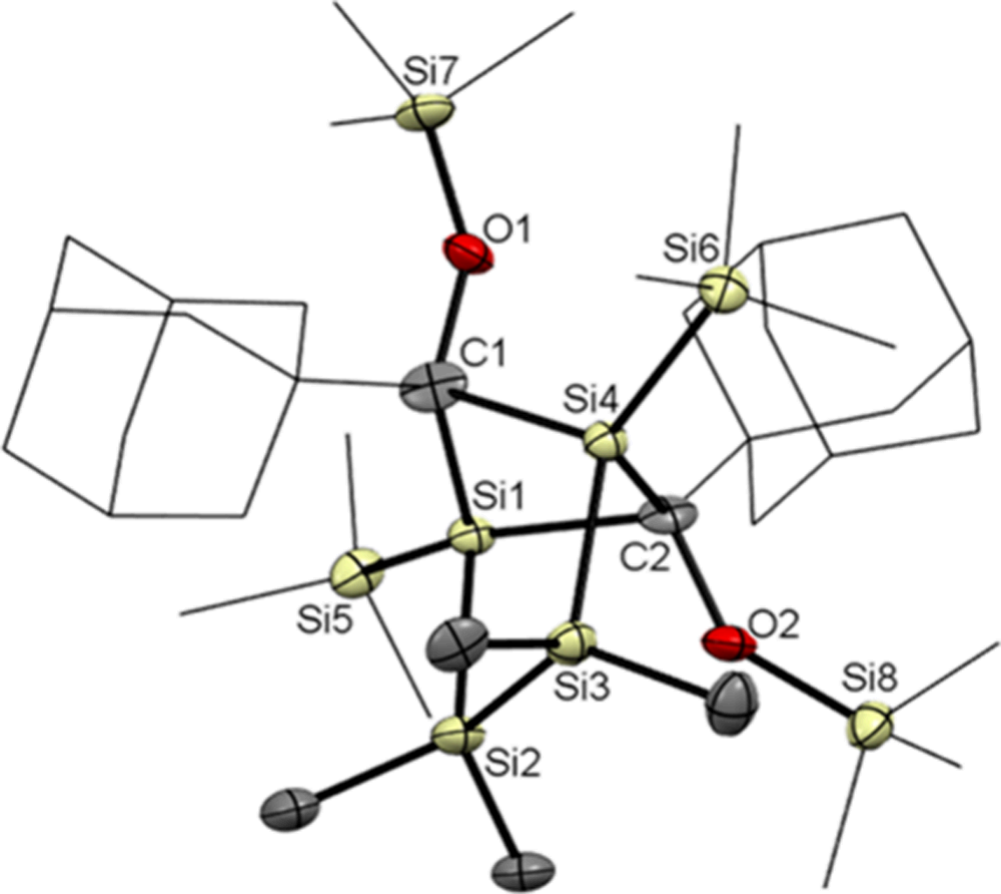
ORTEP representation for compound **11**. Thermal ellipsoids are depicted at the 50% probability level. Hydrogen atoms have been omitted for the sake of clarity, Moreover, adamantyl (Ad) groups and methyl groups are shown as wireframes for the sake of clarity. Selected bond lengths (angstroms) and angles (deg) with estimated standard deviations: Si–Si (mean), 2.3948; Si–O (mean), 1.6345; C–O (mean), 1.4865; Si1–C1, 1.995(13); Si1–C2, 1.986(14); Si4–C1, 1.980(15); Si4–C2, 2.003(13); O1–C1–Si4, 101.5(9); O1–C1–Si1, 116.9(8); O2–C2–Si4, 108.6(8); O2–C2–Si1, 109.7(8); Si1–C2–Si4, 82.6(5); Si1–C1–Si4, 82.9(5).

Moreover, we were able to grow crystals suitable for X-ray analysis, which were obtained by slowly evaporating a concentrated solution of **11** in acetone at room temperature. Compound **11** crystallized in triclinic space group *P*1, and the unit cell contains four molecules (see Figure 8).

**Figure 9:**
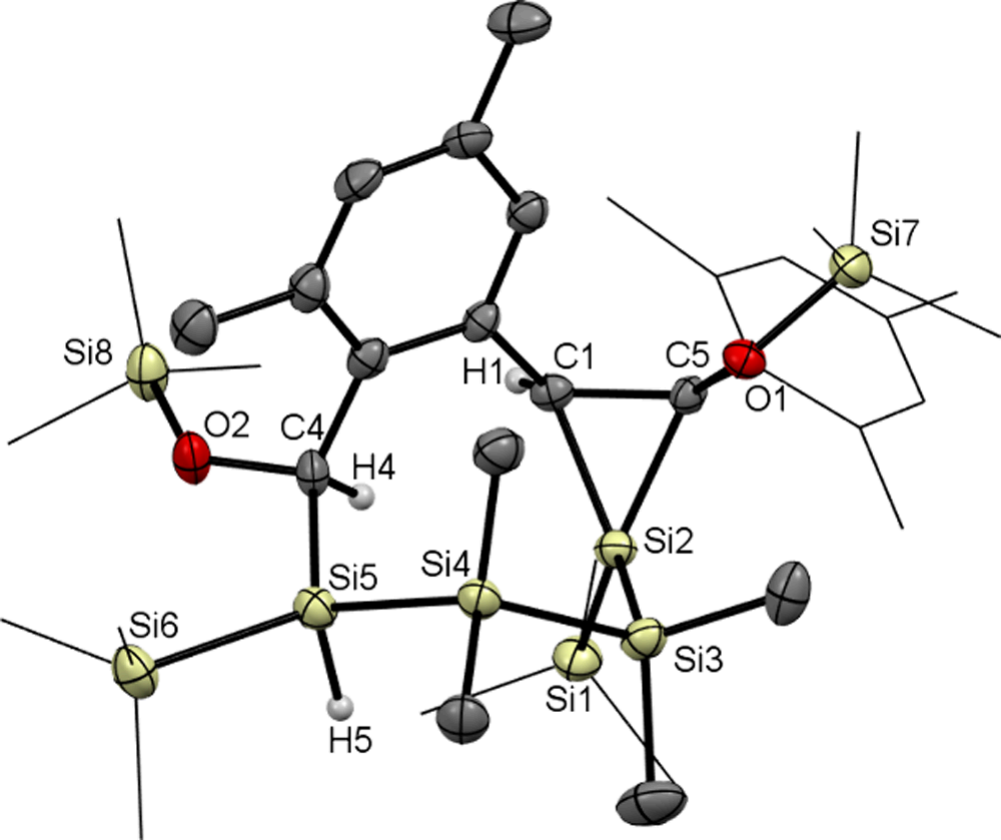
ORTEP representation for compound **12**. Thermal ellipsoids are depicted at the 50% probability level. Hydrogen atoms have been omitted for the sake of clarity, Moreover, one mesityl (Mes) group and methyl groups are shown as wireframes for the sake of clarity. Selected bond lengths (angstroms) and angles (degrees) with estimated standard deviations: Si–Si (mean), 2.3948; Si–O (mean), 1.6345; C–O (mean), 1.4865; Si1–C1, 1.995(13); Si1–C2, 1.986(14); Si4–C1, 1.980(15); Si4–C2, 2.003(13); O1–C1–Si4, 101.5(9); O1–C1–Si1, 116.9(8); O2–C2–Si4, 108.6(8); O2–C2–Si1, 109.7(8); Si1–C2–Si4, 82.6(5); Si1–C1–Si4, 82.9(5).

**Figure 10:**
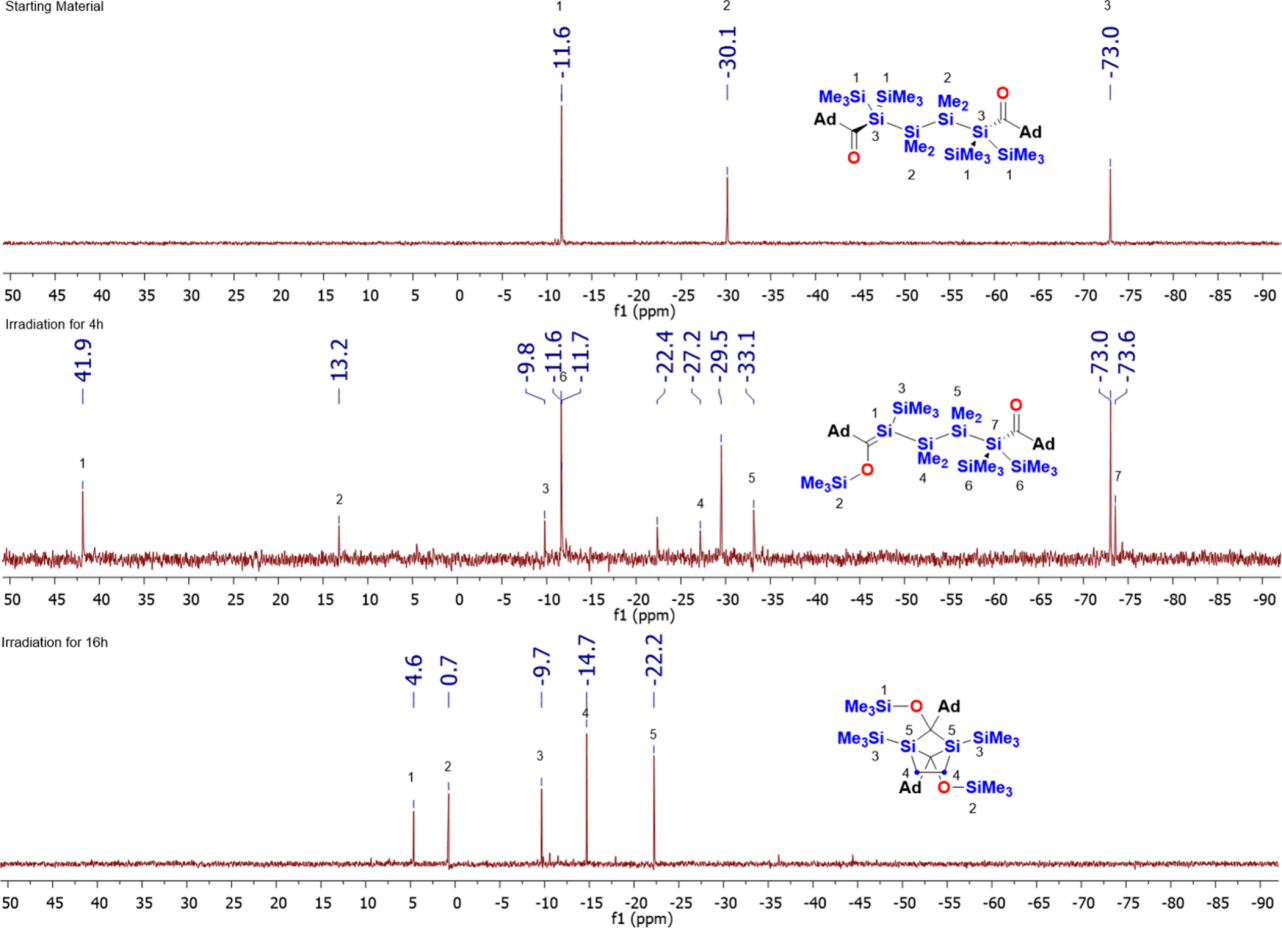
^29^Si NMR spectra before and after irradiation at λ = 405 nm for 4 and 16 h, including the assignment of the observed resonance lines for **10** and **11**.

**Scheme 9:**
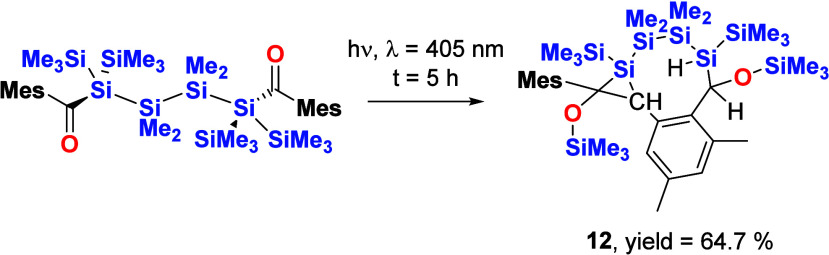
Photolysis of **4**

**Figure 11:**
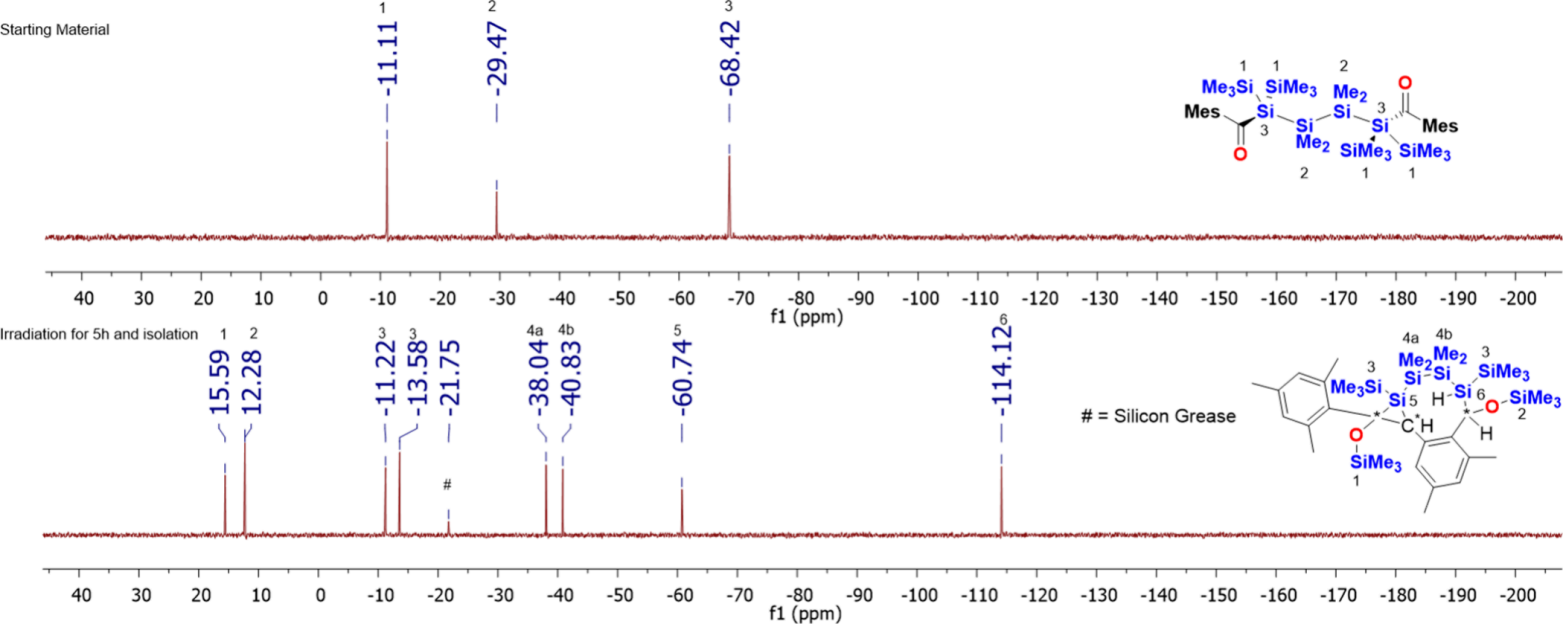
^29^Si NMR spectra before and after irradiation at λ = 405 nm for 5 h and isolation via crystallization in acetone, including the assignment of the observed resonance lines for **12**.

Mesityl-substituted bisacylsilane **4** was irradiated for 4 h at λ = 405 nm in benzene and in the absence of air and moisture. The progress was again monitored by ^29^Si NMR spectroscopy. Here we found that the starting material was nearly consumed, and in addition to the selective formation of one product, signals for the Si=C group at δ = 35.6 ppm and for the OSiMe_3_ group at δ = 15.61 ppm were observed, indicating a monosilane formation. After irradiation for an additional 1 h, the starting material was completely consumed (see Figure 11). Final product **12** is air stable and was isolated by crystallization from acetone at room temperature in an excellent yield of 64.7% (see Scheme 9).

Moreover, we were able to grow crystals suitable for X-ray analysis, which were obtained by slowly evaporating a concentrated solution of **12** in acetone at room temperature. Compound **12** crystallized in monoclinic space group *P*2_1/*c*_, and the unit cell contains four molecules. Interestingly, for compound **4**, we would assume a C–H activation at both double bonds, as known in the literature,^18,20,25^ generating a four-membered ring system at both former silene moieties. In contrast to this assumption, compound **4** undergoes an intramolecular ring closure leading to bicyclic derivative **12**, which is confirmed by NMR spectroscopy and X-ray crystallography. Photochemical product **12** contains five chiral centers at Si2, Si5, C1, C4, and C5, with alternating *S* and *R* enantiomers. X-ray crystallography could not clarify if the SRSRS or the RSRSR configuration crystallized (see Figure 9).

**Figure 12:**
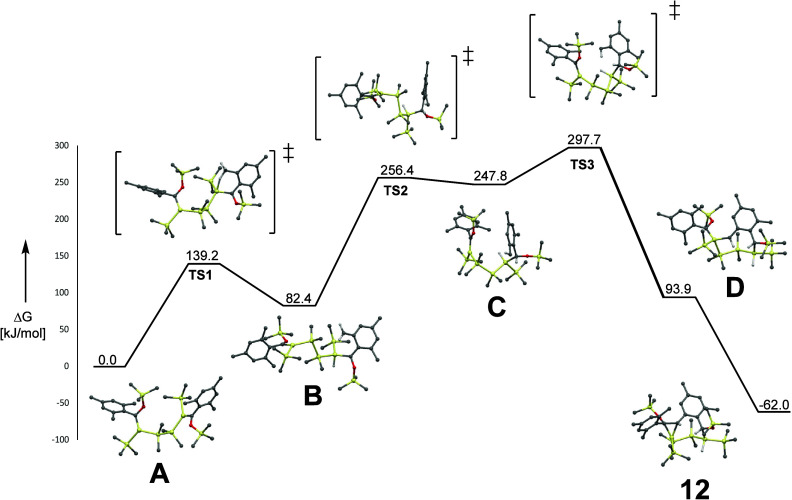
Computed reaction profile of the whole process of all intermediates and TSs using the B3LYP/GD3 functional with the 6-31G** basis set.

**Figure 13:**
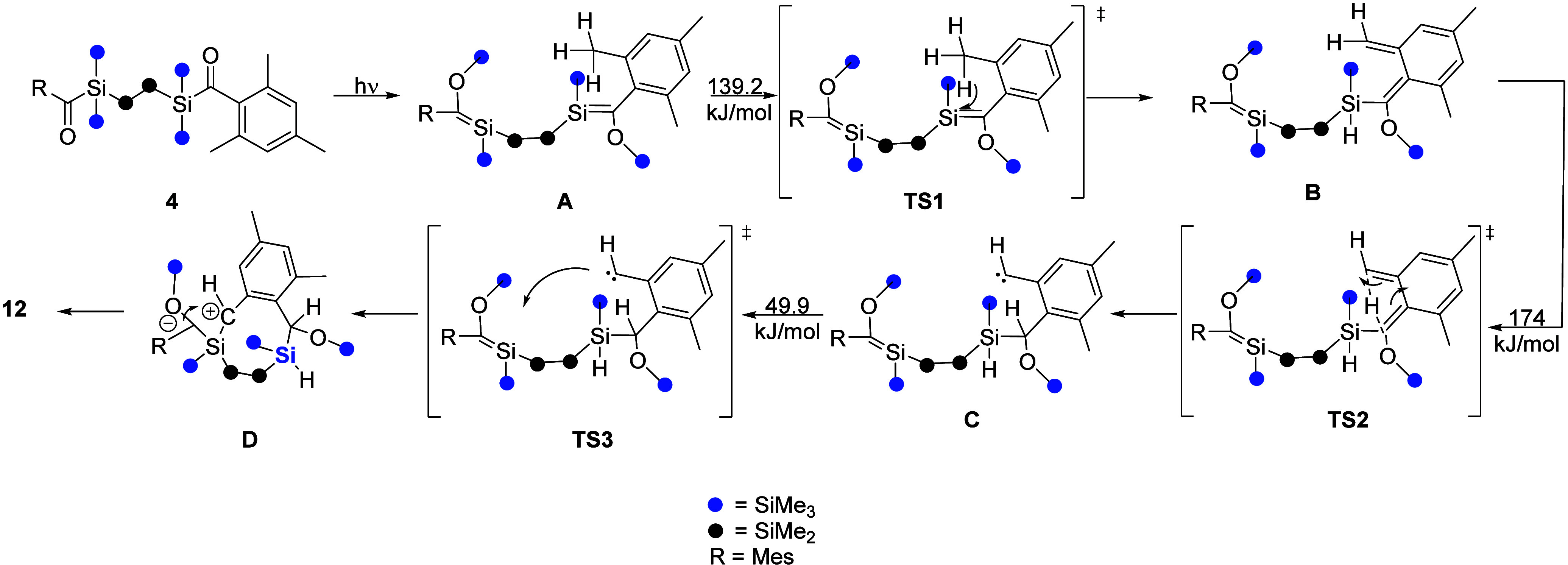
DFT-calculated mechanism with important intermediates and transition states.

The lack of important intermediates after the silene formation toward the ring closure made quantum mechanistic calculations necessary to shed light on the mechanism. The mechanism of this photolysis reaction is proposed in Figures 12 and 13. Due to the high energies during the irradiation, isolation and detection of silene formation on both sides are not possible. The Si backbone shows a 108° conformation (A), which favors the following steps. The first reaction step after silene formation is the transfer of one hydrogen of the *o*-CH_3_ group to the double-bonded silicon (**TS1**). The reaction pathway according to the literature, where first the hydrogen is transferred to the carbon, is unfavored. **B** exhibits a structure with an acyclic double bond. This double bond interferes with the aromatic ring system leading to a nonplanar formation. Another hydrogen is transferred from the now -CH_2_ group to the carbon of the former double bond, leading to a saturated Si–C bond (**TS2**). **C** shows the minimum of the carbene with the saturated Si–C bond. The next transition, **TS3**, is the approach and rotation of the carbene to the remaining Si=C bond. Due to the geometry of this intermediate, the partly negatively charged carbon (-CH) interacts (D) with the second double bond, generating the photochemical and air stable bicyclic endproduct **12**, containing an eight- and three-membered ring system.

We also performed the trapping experiments in MeOH in the presence of Et_3_N. However, for both compounds the main products were again bicyclic derivatives **11** and **12**. We assume that the intramolecular rearrangement also occurs in the presence of MeOH, wehich is the dominant process.

#### Tetrakis(trimethoxysilyl)-1,4-bisacylsilanes

Compounds **5** and **6** were photolyzed at λ = 405 nm in C_6_D_6_ for several hours. In contrast to the other derivatives, silene formation was not detected by NMR analysis. However, we observed different peaks in the O(SiOMe)_3_ region, which speaks to the Brook rearrangement step with unstable silene formation and a low activation barrier for the next step. However, no selective products after prolonged photolysis were formed according to NMR spectroscopy. Consequently, on the basis of the experience gained with compounds **1** and **2**, we stopped our investigations here.

## Conclusion

To conclude, we were able to synthesize and fully characterize novel mono- and bis(acyl)polysilanes. On the basis of TDDFT calculations, their longest wavelength absorptions can be straightforwardly attributed to nσ → π* transitions. Furthermore, we performed photochemistry with all isolated derivatives at a preparative scale. Compounds **1** and **2** undergo a selective Brook-type rearrangement and form an equilibrium with the corresponding dimers. Moreover, mesityl-substituted silene **8a** undergoes a CH activation and forms spirocyclic compound **8c**. On the basis of the lack of crystallization, the instability on silica gel made the isolation of the formed photoproducts impossible. However, the proposed mechanism for the silene formation was supported by trapping experiments with MeOH in the presence of Et_3_N. For tetrakis(trimethylsilyl)-1,4-bisacylsilanes **3** and **4**, the same photochemical experiments were performed. Instead of the expected bissilenes, both compounds undergo highly selective hitherto unknown rearrangements. In case of adamantyl derivative **3**, we observed an unprecedented head-to-tail intramolecular ring closure of the bissilene as the sole product. In contrast, mesityl derivative **4** undergoes a selective and completely stereoselective double C–H activation to the air stable bicyclic system, which is fully characterized and validated by X-ray crystallography. The mechanism of this rearrangement is fully described by DTF calculations. Finally, tetrakis(trimethoxysilyl)bisacylsilanes **5** and **6** underwent unselective photochemical rearrangements.

## Experimental Section

### General Procedures

#### Synthesis

All experiments were performed under a nitrogen atmosphere using standard Schlenk techniques. Solvents were dried using a column solvent purification system.^27^ (Me_2_Si)_2_Cl_2_ (≥98%), KO*t*Bu (>98%), 1-adamantoyl chloride (>98%), 2,4,6-trimethylbenzoyl chloride (>98%), and benzene-*d*_6_ (99.5 atom %, D) were used without any further purification. Dodecamethoxyneopentasilane, 1,1,1,4,4,4-tetrakis(trimethoxylsilyl)-2,2,3,3-tetramethyltetrasilane,^21^ 1,4-dipotassium-1,1,4,4-tetrakis(trimethoxysilyl)tetramethyltetrasilane,^24^ and 1,4-dipotassium-1,1,4,4-tetrakis(trimethylsilyl)tetramethyltetrasilane^23^ were synthesized according to the literature. Et_3_N was dried with Linde-type 4A molecular sieves according to the literature.^28^ Methanol was dried with magnesium and iodine according to published procedures.^28^ For the measurement of air-sensitive samples, benzene-*d*_6_ was additionally dried above a sodium/potassium alloy during a 12 h reflux. Melting points were determined using the Stuart SMP50 apparatus and are uncorrected. Elemental analyses were carried out on a Hanau Vario Elementar EL apparatus.

#### Irradiation Experiments

Photochemical experiments were performed on a self-made photoreactor with arrays of various LEDs having an emission spectrum centered at 365, 405, 550, and 590 nm. The photoreactor setup comprises 24 LEDs per defined wavelength. Experiments were performed with a total electrical output power of 25 W. Additionally, an integrated cooling system maintained a constant reaction temperature of 23 °C.

#### UV/Vis Spectroscopy

UV/vis spectra were acquired using a PerkinElmer Lambda 5 spectrometer.

#### Fourier Transform Infrared (FT-IR) Spectroscopy

The steady-state and time-resolved FT-IR spectra in solution were recorded on a Bruker Alpha spectrometer running OPUS 7.5 software in transmission mode.

#### NMR Spectroscopy

^1^H, ^13^C, and ^29^Si NMR spectra were recorded on a Varian INOVA 400, a Varian INOVA 300, a 200 MHz Bruker AVANCE DPX, or a Bruker Avance 300 MHz spectrometer in benzene-*d*_6_ and referenced versus TMS using the internal ^2^H lock signal of the solvent.

#### DFT Calculations

All structures of the investigated compounds have been optimized and verified by vibrational frequency calculations at the DFT level using B3LYP/6-31G** together with a Grimme dispersion correction (GD3).^29^ TDDFT calculations at the B3LYP(GD3)/6-311++G** level were applied to compute five vertical excitations. All calculations were performed using Gaussian16.^30^

#### X-ray Crystallography

All crystals suitable for single-crystal X-ray diffractometry were removed from a vial or Schlenk flask and immediately covered with a layer of silicone oil. A single crystal was selected, mounted on a glass rod on a copper pin, and placed in a cold N_2_ stream. XRD data were collected for compound **3**, **4**, **7b**, **11**, and **12** on a Bruker APEX II diffractometer using an Incoatec microfocus sealed tube of Mo Kα radiation (λ = 0.71073 Å) and a CCD area detector. Empirical absorption corrections were applied using SADABS or TWINABS.^31,32^ The structures were determined with either the use of direct methods or the intrinsic phasing option in SHELXT and refined by the full-matrix least-squares procedures in SHELXL^33−35^ or Olex2.^36^ The space group assignments and structural solutions were evaluated using PLATON.^37,38^ Non-hydrogen atoms were refined anisotropically. Hydrogen atoms were located in either a difference map or calculated positions corresponding to standard bond lengths and angles. Disorder was handled by modeling the occupancies of the individual orientations using free variables to refine the respective occupancy of the affected fragments (PART).^39^Table S1 contains crystallographic data and details of measurements and refinement for all compounds. Crystallographic data (excluding structure factors) have been deposited with the Cambridge Crystallographic Data Centre (CCDC): 2314683 for **3**, 2314681 for **4**, 2314684 for **7b**, 2314680 for **11**, and 2314682 for **12**.

### Experimental Procedures

#### Synthesis of **1**

A solution of [Si(OMe)_3_]_3_SiK in 20 mL of THF was freshly prepared from 1.00 g of Si[Si(OMe)_3_]_4_ (1.95 mmol, 1.00 equiv) and 0.23 g of KO^*t*^Bu (2.05 mmol, 1.05 equiv) and slowly added to a solution of 0.41 g (2.05 mmol, 1.05 equiv) of 1-adamantoyl chloride in 20 mL of *n*-pentane at −78 °C. Subsequently, the mixture was stirred for an additional 30 min at −78 °C, allowed to warm to room temperature, and finally stirred for an additional 60 min. The solvent was removed via vacuum. Twenty milliliters of *n*-pentane was added, and the salts were filtered off. Removal of the solvent afforded 0.67 g of **1** (62%) as a colorless oil. Anal. Calcd (%) for C_20_H_42_O_10_Si_4_: C, 43.29; H, 7.63. Found: C, 43.10; H, 7.59. ^29^Si NMR (C_6_D_6_, TMS): δ −42.0 [*Si*(OMe)_3_], −107.6 (*Si*q). ^13^C NMR (C_6_D_6_, TMS): δ 242.7 (*C=*O), 50.5 (O*C*H_3_), 53.2, 37.1, 37.1, 28.8 (Ad-*C*). ^1^H NMR (C_6_D_6_, TMS): δ 3.62 (s, 27H, OC*H*_3_), 2.00 (s, 9H, Ad-C*H*_2_ and -C*H*), 1.68, 1.66 (m, 6H, Ad-C*H*_2_). IR (neat): ν(C=O) 1628, 1452 (m) cm^–1^. UV/vis: λ (nm) [ε (L mol^–1^ cm^–1^)] 380 (86).

#### Synthesis of **2**

A solution of [Si(OMe)_3_]_3_SiK in 20 mL of THF was freshly prepared from 1.00 g of Si[Si(OMe)_3_]_4_ (1.95 mmol, 1.00 equiv) and 0.23 g of KO^*t*^Bu (2.05 mmol, 1.05 equiv) and slowly added to a solution of 0.38 g (2.05 mmol, 1.05 equiv) of 2,4,6-trimethylbenzoyl chloride in 20 mL of *n*-pentane at −78 °C. Subsequently, the mixture was stirred for an additional 30 min at −78 °C, allowed to warm to room temperature, and finally stirred for an additional 60 min. The solvent was removed via vacuum. Twenty milliliters of *n*-pentane was added, and the salts filtered off. Removal of the solvent afforded 0.57 g of **2** (55%) as a slightly yellow oil. Anal. Calcd (%) for C_19_H_38_O_10_Si_4_: C, 42.35; H, 7.11. Found: C, 42.45; H, 7.05%. ^29^Si NMR (C_6_D_6_, TMS): δ −41.5 [*Si*(OMe)_3_], −97.9 (*Si*q). ^13^C NMR (C_6_D_6_, TMS): δ 241.8 (*C=*O), 146.9, 137.7, 132.4, 128.7 (Mes-*C*), 50.3 (O*C*H_3_), 21.0, 19.0 (Mes-*C*H_3_). ^1^H NMR (C_6_D_6_, TMS): δ 6.62 (s, 2H, Mes-*H*), 3.50 (s, 27H, OC*H*_3_), 2.32 (s, 6H, Mes-C*H*_3_), 2.06 (s, 3H, Mes-C*H*_3_). IR (neat): ν(C=O) 1624, 1608, 1451 (m) cm^–1^. UV/vis: λ (nm) [ε (L mol^–1^ cm^–1^)] 355 (92), 370 (113), 385 (123), 404 (85).

#### Synthesis of **3**

A solution of (Me_3_Si)_2_KSi_2_Me_4_(SiMe_3_)_2_K in 20 mL of DME was freshly prepared from 3.00 g (4.91 mmol, 1.00 equiv) of (Me_3_Si)_3_Si_2_Me_4_(SiMe_3_)_3_ and 1.16 g (10.3 mmol, 2.10 equiv) of KO^*t*^Bu and slowly added to a solution of 2.05 g (10.3 mmol, 2.10 equiv) of 1-adamantoyl chloride in 50 mL of diethyl ether at −78 °C. Subsequently, the mixture was stirred for an additional 30 min at −78 °C, allowed to warm to room temperature, and finally stirred for an additional 60 min. After aqueous workup with 100 mL of 3% sulfuric acid, the organic layer was separated and dried over Na_2_SO_4_ and the solvents were removed on a rotary evaporator. Drying under vacuo (0.02 mbar) and recrystallization from an acetone solution at −30 °C afforded 3.40 g of **3** (87%) as a white solid. Mp: 187–189 °C. Anal. Calcd (%) for C_38_H_78_O_2_Si_8_: C, 57.65; H, 9.93. Found: C, 56.64; H, 9.70. ^29^Si NMR (CDCl_3_, TMS): δ −11.5 (*Si*Me_3_), −30.1 (*Si*Me_2_), −72.9 (*Si*C=O). ^13^C NMR (CDCl_3_, TMS): δ 249.8 (*C*=O), 51.9, 37.4, 36.8, 28.2 (Ad-*C*), 3.0 [Si(*C*H_3_)_3_], 0.4 [Si(*C*H_3_)_2_]. ^1^H NMR (CDCl_3_, TMS): δ 2.07 (s, 6H, Ad-C*H*_2_ and -C*H*), 1.68, 1.66 (m, 24H, Ad-C*H*_2_), 0.35 [s, 12H, Si(C*H*_3_)_2_], 0.28 [s, 36H, Si(C*H*_3_)_3_]. IR (neat): ν(C=O) 1622, 1451 (m) cm^–1^. UV/vis: λ (nm) [ε (L mol^–1^ cm^–1^)] 372 (222).

#### Synthesis of **4**

A solution of (Me_3_Si)_2_KSi_2_Me_4_(SiMe_3_)_2_K in 20 mL of DME was freshly prepared from 3.00 g (4.91 mmol, 1.00 equiv) of (Me_3_Si)_3_Si_2_Me_4_(SiMe_3_)_3_ and 1.16 g (10.3 mmol, 2.10 equiv) of KO^*t*^Bu and slowly added to a solution of 1.88 g (10.3 mmol, 2.10 equiv) of 2,4,6-trimethylbenzoyl chloride in 50 mL of diethyl ether at −78 °C. Subsequently, the mixture was stirred for an additional 30 min at −78 °C, allowed to warm to room temperature, and finally stirred for an additional 60 min. After aqueous workup with 100 mL of 3% sulfuric acid, the organic layer was separated and dried over Na_2_SO_4_ and the solvents were removed on a rotary evaporator. Drying under vacuo (0.02 mbar) and recrystallization from an acetone solution at −30 °C afforded 3.12 g of **4** (84%) as a yellow solid. Mp: 160–161 °C. Anal. Calcd (%) for C_36_H_70_O_2_Si_8_: C, 56.92; H, 9.29. Found: C, 56.95; H, 9.35. ^29^Si NMR (C_6_D_6_, TMS): δ −11.1 (*Si*Me_3_), −29.5 (*Si*Me_2_), −68.4 (*Si*C=O). ^13^C NMR (C_6_D_6_, TMS): δ 247.5 (*C*=O), 147.5, 137.9, 131.7, 129.1 (Mes-*C*), 21.0, 20.2 (Mes-*C*H_3_), 2.5 [Si(*C*H_3_)_3_], 0.4 [Si(*C*H_3_)_2_]. ^1^H NMR (C_6_D_6_, TMS): δ 6.59 (s, 4H, Mes-*H*), 2.22 (s, 12H, Mes-C*H*_3_), 2.05 (s, 6H, Mes-C*H*_3_), 0.69 [s, 12H, Si(C*H*_3_)_2_], 0.31 [s, 36H, Si(C*H*_3_)_3_]. IR (neat): ν(C=O) 1606, 1592 (m) cm^–1^. UV/vis: λ (nm) [ε (L mol^–1^ cm^–1^)] 371 (220), 388 (308), 404 (265).

#### Synthesis of **5**

A solution of [(MeO)_3_Si]_2_KSi_2_Me_4_[Si(OMe)_3_]_2_K in 20 mL of THF was freshly prepared from 3.00 g (3.33 mmol, 1.00 equiv) of [(MeO)_3_Si]_3_Si_2_Me_4_[Si(OMe_3_)]_3_ and 0.79 g (7.00 mmol, 2.10 equiv) of KO^*t*^Bu and slowly added to a solution of 1.39 g (7.00 mmol, 2.10 equiv) of 1-adamantoyl chloride in 50 mL of toluene at −78 °C. Subsequently, the mixture was stirred for an additional 30 min at −78 °C, allowed to warm to room temperature, and finally stirred for an additional 60 min. Afterward, the volatile compounds were removed under reduced pressure. Again, toluene was added to filter the polymeric residue. Drying under vacuo (0.02 mbar) and recrystallization from a *n*-pentane solution at −70 °C afforded 2.30 g of **5** (70%) as a white solid. Mp: 181–182 °C. Anal. Calcd (%) for C_38_H_78_O_14_Si_8_: C, 46.40; H, 7.99. Found: C, 46.56; H, 8.20. ^29^Si NMR (C_6_D_6_, TMS): δ −30.7 (*Si*Me_2_), −40.7 [*Si*(OMe)_3_], −93.0 (*Si*C=O). ^13^C NMR (C_6_D_6_, TMS): δ 245.3 (*C*=O), 50.6 (O*C*H_3_), 52.9, 37.6, 37.1, 28.9 (Ad-*C*), −1.5 [Si(*C*H_3_)_2_]. ^1^H NMR (C_6_D_6_, TMS): δ 3.63 (s, 36H, OC*H*_3_), 2.10 (s, 18H, Ad-C*H*_2_ and -C*H*), 1.79,1.68 (m, 12H, Ad-C*H*_2_), 0.93 [s, 12H, Si(C*H*_3_)_2_]. IR (neat): ν(C=O) 1606, 1592 (m) cm^–1^. UV/vis: λ (nm) [ε (L mol^–1^ cm^–1^)] 352 (225).

#### Synthesis of **6**

A solution of [(MeO)_3_Si]_2_KSi_2_Me_4_[Si(OMe)_3_]_2_K in 20 mL of THF was freshly prepared from 3.00 g (3.33 mmol, 1.00 equiv) of [(MeO)_3_Si]_3_Si_2_Me_4_[Si(OMe_3_)]_3_ and 0.79 g (7.00 mmol, 2.10 equiv) of KO^*t*^Bu and slowly added to a solution of 1.28 g (7.00 mmol, 2.10 equiv) of 2,4,6-trimethylbenzoyl chloride in 50 mL of toluene at −78 °C. Subsequently, the mixture was stirred for an additional 30 min at −78 °C, allowed to warm to room temperature, and finally stirred for an additional 60 min. Afterward, the volatile compounds were removed under reduced pressure. Again, toluene was added to filter the polymeric residue. Drying under vacuo (0.02 mbar) and recrystallization from an *n*-pentane solution at −70 °C afforded 2.70 g of **6** (85%) as a yellow solid. Mp: 165–167 °C. Anal. Calcd (%) for C_36_H_70_O_14_Si_8_: C, 45.44; H, 7.41. Found: C, 45.69; H, 7.63. ^29^Si NMR (C_6_D_6_, TMS): δ −30.9 (*Si*Me_2_), −42.0 [*Si*(OMe)_3_], −84.3 (*Si*C=O). ^13^C NMR (C_6_D_6_, TMS): δ 244.3 (*C*=O), 147.3, 137.7, 132.5, 128.9 (Mes-*C*), 50.5 (O*C*H_3_), 21.1, 19.6 (Mes-*C*H_3_), −2.4 [Si(*C*H_3_)_2_]. ^1^H NMR (C_6_D_6_, TMS): δ 6.66 (s, 4H, Mes-*H*), 3.55 (s, 36H, OC*H*_3_), 2.45 (s, 12H, Mes-C*H*_3_), 2.07 (s, 6H, Mes-C*H*_3_), 0.87 [s, 12H, Si(C*H*_3_)_2_]. IR (neat): ν(C=O) 1606, 1592 (m) cm^–1^. UV/vis: λ (nm) [ε (L mol^–1^ cm^–1^)] 359 (300), 374 (424), 390 (502), 406 (389).

#### Photolysis of **1** in C_6_D_6_

First, 57 mg (0.10 mmol) of **1** in C_6_D_6_ (0.6 mL) in an NMR tube was photolyzed with the photoreactor at λ = 405 nm for 2 h. At this time, NMR analysis of the resulting clear solution showed that the starting material had been consumed completely. A mixture of silene **7a** and dimer **7b** could be obtained and characterized by ^29^Si NMR spectroscopy. Complete conversion to the dimer or separation was not successful.

For **7a**. ^29^Si NMR (C_6_D_6_, TMS): δ 34.3 (*Si*=C), −36.3 [*Si*(*O*Me)_3_], −51.7 [*Si*(*O*Me)_3_], −56.2 [O*Si*(OMe)_3_]. ^1^H NMR (C_6_D_6_, TMS): δ 3.67 [s, 9H, Si(OC*H*_3_)_3_], 3.61 [s, 9H, Si(OC*H*_3_)_3_], 3.55 [s, 9H, Si(OC*H*_3_)_3_], 1.5–2.0 (m, 16H, Ad).

For **7b**. ^29^Si NMR (C_6_D_6_, TMS): δ −41.9 [*Si*(*O*Me)_3_], −42.0 [*Si*(*O*Me)_3_], −73.0 (*Si*C), −90.8 [O*Si*(OMe)_3_]. ^1^H NMR (C_6_D_6_, TMS): δ 3.71 [s, 18H, (Si(OMe)_3_], 3.67 [s, 18H, Si(OMe)_3_], 3.55 [s, 18H, Si(OMe)_3_], 1.5–2.0 (m, 16H, Ad).

#### Photolysis of **2** in C_6_D_6_

First, 56 mg (0.10 mmol) of **2** in C_6_D_6_ (0.6 mL) in an NMR tube was photolyzed with the photoreactor at λ = 405 nm for 25 min. At this time, NMR analysis of the resulting solution showed that the starting material had been consumed completely. At this stage, the signals for three different products (silene **8a**, the corresponding dimer **8b**, and C–H bond activation product **8c**) were observed. Upon prolonged irradiation, silene **8a** is completely converted into **8c**. After removal of the solvents, **8c** could be obtained in nearly quantitative yield as a colorless oil with small amounts of uncharacterizable side products.

For **8a**. ^29^Si NMR (C_6_D_6_, TMS): δ 30.3 (*Si*=C), −35.6 [*Si*(OMe)_3_], −50.3 [*Si*(OMe)_3_], −51.7 [*Si*(OMe)_3_]. ^13^C NMR (C_6_D_6_, TMS): δ 201.79 (Si=C).

For **8b**. ^29^Si NMR (C_6_D_6_, TMS): δ −41.5 [*Si*(OMe)_3_], −42.6 [*Si*(OMe)_3_], −60.1 (*Si*C), −86.3 [O*Si*(OMe)_3_]. ^1^H NMR (C_6_D_6_, TMS): δ 6.80 (s, 4H, Mes-*H*), 3.72 [s, 18H, Si(OC*H*_3_)_3_], 3.35 [s, 18H, Si(OC*H*_3_)_3_], 3.31 [s, 18H, Si(OC*H*_3_)_3_], 2.70 (s, 12H, Mes-C*H*_3_), 2.09 (s, 6H, Mes-C*H*_3_).

For **8c**. ^29^Si NMR (C_6_D_6_, TMS): δ −40.8 [*Si*(OMe)_3_], −41.9 [*Si*(OMe)_3_], −79.6 (*Si*H), −81.7 [O*Si*(OMe)_3_]. ^13^C NMR (C_6_D_6_, TMS): δ 146.2, 140.4, 138.5, 133.3, 129.2, 121,1 (Mes*-C*), 72.9 (SiC), 50.6, 49.7, 49.6 [Si(O*Me*)_3_], 46.3 (*C*H), 21.8, 16.6 (Mes-*C*H_3_). ^1^H NMR (C_6_D_6_, TMS): δ 6.74 (s, 1H, Mes-*H*), 6.70 (s, 1H, Mes-*H*), 4.30 (s, 1H, SiH), 3.61 [s, 9H, Si(OC*H*_3_)_3_], 3.41 [s, 9H, Si(OC*H*_3_)_3_], 3.40 [s, 9H, Si(OC*H*_3_)_3_], 2.51 (s, 3H, Mes-C*H*_3_), 2.11 (s, 3H, Mes-C*H*_3_).

#### Preparation of **9a**. Photolysis of **1** in C_6_H_6_/MeOH

First, 0.5 g (0.9 mmol) of **1** and 3 drops of anhydrous Et_3_N in 6.0 mL of C_6_H_6_ and 1 mL of methanol were photolyzed at λ = 405 nm for 6 h. Then, NMR analysis of the resulting clear solution showed that the starting material had been consumed completely. After removal of the solvents, **9a** could be obtained in nearly quantitative yield (90%, 0.46 g) as a white oil with small amounts of side products. Anal. Calcd (%) for C_21_H_46_O_11_Si_4_: C, 42.97; H, 7.90. Found: C, 43.15; H, 7.70. ^29^Si NMR (C_6_D_6_, TMS): δ −9.9 (*Si*OMe), −47.3 [*Si*(OMe)_3_], −47.9 [*Si*(OMe)_3_], −80.7 [O*Si*(OMe)_3_]. ^13^C NMR (C_6_D_6_, TMS): δ 79.2 (*C*H), 53.6 (O*Me*), 50.9, 49.9. 49.8 [Si(O*Me*)_3_], 39.9, 37.3, 30.0, 28.9 (Ad-*C*). ^1^H NMR (C_6_D_6_, TMS): δ 4.31 (s, 1H, C*H*), 3.72 (s, 3H, SiOC*H*_3_), 3.66 [s, 9H, Si(OC*H*_3_)_3_], 3.62 [s, 18H, Si(OC*H*_3_)_3_], 2.11–1.82 (m, 15H, Ad-*H*).

#### Preparation of **9b**. Photolysis of **2** in C_6_H_6_/MeOH

First, 0.5 g (0.9 mmol) of **2** and 3 drops of anhydrous Et_3_N in 6 mL of C_6_H_6_ and 1 mL of methanol were photolyzed with the photoreactor at λ = 405 nm for 6 h. Then, NMR analysis of the resulting clear solution showed that the starting material had been consumed completely. After removal of the solvents, **9b** could be obtained as a white oil (91%, 0.47 g) Anal. Calcd (%) for C_20_H_42_O_11_Si_4_: C, 42.08; H, 7.42. Found: C, 42.15; H, 7.50. ^29^Si NMR (C_6_D_6_, TMS): δ −3.7 (*Si*OMe), −49.0 [*Si*(OMe)_3_], −49.0 [*Si*(OMe)_3_], −78.9 [O*Si*(OMe)_3_]. ^13^C NMR (C_6_D_6_, TMS): δ 138.8, 135.8, 135.5, 135.0, 130.2, 128.6, (Mes*-C*), 68.2 (*C*H), 54.2 (O*Me*), 50.5, 49.7. 49.6 [Si(O*Me*)_3_], 21.5, 20.6, 20.5 (Mes-*C*H_3_). ^1^H NMR (C_6_D_6_, TMS): δ 6.82 (s, 1H, Mes-*H*), 6.74 (s, 1H, Mes-*H*), 5.87 (s, 1H, C*H*), 3.57 [s, 9H, Si(OC*H*_3_)_3_], 3.49 [s, 9H, Si(OC*H*_3_)_3_], 3.49 (s, 3H, SiOC*H*_3_), 3.38 [s, 9H, Si(OC*H*_3_)_3_], 2.84 (s, 3H, Mes-C*H*_3_), 2.58 (s, 3H, Mes-C*H*_3_), 2.08 (3H, Mes-C*H*_3_).

#### Photolysis of **3**

First, 150 mg (0.18 mmol) of **3** in 10 mL of C_6_H_6_ was photolyzed with the photoreactor at λ = 405 nm for 4 h. Reaction control by ^29^Si NMR showed the formation of monosilane intermediate **10**, bicyclic structure **11**, and the starting material. Subsequently, the sample was photolyzed for additional 16 h to ensure complete conversion of the starting material. Removal of the solvents and recrystallization in *n*-pentane at −30 °C afforded 92 mg (61.5%) of **11** as colorless crystals. For **10**. ^29^Si NMR (C_6_D_6_, TMS): δ 41.6 (*Si*=C), 13.2 (O*Si*Me_3_), −9.8 (SiMe_3_), −27.2 (SiMe_2_), −33.1 (SiMe_2_), −73.6 (*Si*q).

For **11**. Mp: 96–101 °C. Anal. Calcd (%) for C_38_H_78_O_2_Si_8_: C, 57.65; H, 9.93. Found: C, 57.59; H, 9.85. ^29^Si NMR (C_6_D_6_, TMS): δ 4.3 (O*Si*Me_3_), 0.4 (O*Si*Me_3_), −10.1 (*Si*Me_3_), −15.1 (*Si*q), −22.6 (SiMe_2_). ^1^H NMR (C_6_D_6_, TMS): δ 2.76–1.61 (m, 30H, Ad*-H*), 0.66 [s, 6H, Si(C*H*_3_)_2_], 0.64 [s, 6H, Si(C*H*_3_)_2_], 0.45 [s, 18H, Si(C*H*_3_)_3_], 0.44 [s, 9H, Si(C*H*_3_)_3_], 0.43 [s, 9H, Si(C*H*_3_)_3_]. ^13^C NMR (C_6_D_6_, TMS): δ 41.3, 41.1, 41.0, 36.6, 29.5, 29.2 (Ad-*C*), 8.1, 6.8 (Si*Me*_3_), 5.5, 3.7 (Si*Me*_2_), 3.1 (S*iMe*_3_).

#### Photolysis of **4**

First, 150 mg (0.2 mmol) of **4** in C_6_D_6_ (0.6 mL) in an NMR tube was photolyzed with the photoreactor at λ = 405 nm for 5 h. Then, NMR analysis of the resulting solution showed that the starting material had been consumed completely. Recrystallization in acetone at room temperature afforded 97.1 mg (64.7%) of **12** as colorless crystals. Anal. Calcd (%) for C_36_H_70_O_2_Si_8_: C, 56.92; H, 9.29. Found: C, 57.25; H, 9.51. ^29^Si NMR (C_6_D_6_, TMS): δ 16.0 (O*Si*Me_3_), 12.7 (O*Si*Me_3_), −10.8 (*Si*Me_3_), −13.2 (*Si*Me_3_), −37.6 (*Si*Me_2_), −60.3 (*Si*H), −113.7 (*Si*q). ^1^H NMR (C_6_D_6_, TMS): δ 7.65 (s, 1H, Mes-*H*), 6.86 (s, 1H, Mes-*H*), 6.79 (s, 1H, Mes-*H*), 6.75 (s, 1H, Mes-*H*), 6.61 (d, 1H, C*H*), 4.12 (d, 1H, Si*H*), 2.98 (s, 1H, C*H*), 2.87 (s, 3H, Mes-C*H*_3_), 2.69 (s, 3H, Mes-C*H*_3_), 2.64 (s, 3H, Mes-C*H*_3_), 2.29 (s, 3H, Mes-C*H*_3_), 2.12 (s, 3H, Mes-C*H*_3_), 0.49 [s, 3H, Si(C*H*_3_)_2_], 0.46 [s, 9H, Si(C*H*_3_)_3_], 0.40 [s, 3H, Si(C*H*_3_)_2_], 0.24 [s, 3H, Si(C*H*_3_)_2_], 0.15 [s, 9H, Si(C*H*_3_)_3_], 0.11, 0.07 [s, 9H, Si(C*H*_3_)_3_], −0.41 (s, 3H, SiMe_2_). ^13^C NMR (C_6_D_6_, TMS): δ 140.5, 140.1, 138.2, 137.1 136.6, 136.1, 135.8, 133.7, 130.6, 129.9, 129.7, 127.9, 127.6 (Mes-*C*), 69.7, 65.2 (*CH*), 30.1 (*Cq*), 22.8, 22.7, 21.5, 20.9, 20.7 (Mes-*C*H_3_), 2.5, 1.6, 0.6, 0.3 (Si*Me*_3_), −3.3, −4.2, −5.0, −7.1 (Si*Me*_2_)

#### Photolysis of **3** in C_6_H_6_/MeOH

First, 150 mg (0.18 mmol) of **3** and 3 drops of anhydrous Et_3_N in 2.0 mL of C_6_H_6_ and 1 mL of methanol were photolyzed at λ = 405 nm for 6 h. After removal of the solvents, **11** was obtained as the only characterizable compound.

#### Photolysis of **4** in C_6_H_6_/MeOH

First, 150 mg (0.2 mmol) of **4** and 3 drops of anhydrous Et_3_N in 2.0 mL of C_6_H_6_ and 1 mL of methanol were photolyzed at λ = 405 nm for 6 h. After removal of the solvents, **12** was obtained as the only characterizable compound.

## Supporting Information Available

NMR spectra and UV/vis spectra (PDF)
X-ray crystallographic information (XYZ)

## Supplementary Material

om3c00531_si_001.pdf

om3c00531_si_002.xyz
