# Supplementary material for: Synthesis and Photochemistry of Tris(trimethoxysilyl)acyl-silanes and 1,4-Tetrakis(silyl)-1,4-bisacylsilanes
Source: Organometallics. 2024 Feb 19;43(16):1713–25. doi: 10.1021/acs.organomet.3c00531 (PMC11351433; doi:10.1021/acs.organomet.3c00531)
Supplement: Supplementary file 1 — om3c00531_si_001.pdf [file om3c00531_si_001.pdf]

Supporting Information

Synthesis and Photochemistry of Tris(trimethoxysilyl)acylsilanes and 1,4-Tetrakis(silyl)-1,4-bisacylsilanes

Thomas Lainer, Sabrina D. Pueschmann, Ana Torvisco, Roland C. Fischer, Michaela Flock and Michael Haas*

Institute of Inorganic Chemistry, Graz University of Technology, Stremayrgasse 9/IV, 8010 Graz (Austria)
*E-mail: michael.haas@tugraz.at

Table of Content

Analytical Section 4

NMR-Spectroscopy 4

Figure S1: ^{1}H NMR spectrum of 1 (C_6D_6 solution, vs ext. TMS, ppm) 4

Figure S2: ^{13}C NMR spectrum of 1 (C_6D_6 solution, vs ext. TMS, ppm) 4

Figure S3: ^{29}Si NMR spectrum of 1 (C_6D_6 solution, vs ext. TMS, ppm) 5

Figure S4: ^{1}H NMR spectrum of 2 (C_6D_6 solution, vs ext. TMS, ppm) 5

Figure S5: ^{13}C NMR spectrum of 2 (C_6D_6 solution, vs ext. TMS, ppm) 6

Figure S6: ^{29}Si NMR spectrum of 2 (C_6D_6 solution, vs ext. TMS, ppm) 6

Figure S7: ^{1}H NMR spectrum of 3 ($CDCl_3$ solution, vs ext. TMS, ppm) 7

Figure S8: ^{13}C NMR spectrum of 3 ($CDCl_3$ solution, vs ext. TMS, ppm) 7

Figure S9: ^{29}Si NMR spectrum of 3 ($CDCl_3$ solution, vs ext. TMS, ppm) 8

Figure S10: ^{1}H NMR spectrum of 4 (C_6D_6 solution, vs ext. TMS, ppm) 8

Figure S11: ^{13}C NMR spectra of 4 (C_6D_6 solution, vs ext. TMS, ppm) 9

Figure S12: ^{29}Si NMR spectra of 4 (C_6D_6 solution, vs ext. TMS, ppm) 9

Figure S13: ^{1}H NMR spectra of 5 (C_6D_6 solution, vs ext. TMS, ppm) 10

Figure S14: ^{13}C NMR spectra of 5 (C_6D_6 solution, vs ext. TMS, ppm) 10

Figure S15: ^{29}Si NMR spectra of 5 (C_6D_6 solution, vs ext. TMS, ppm) 11

Figure S16: ^{1}H NMR spectrum of 6 (C_6D_6 solution, vs ext. TMS, ppm) 11

Figure S17: ^{13}C-NMR spectrum of 6 (C_6D_6 solution, vs ext. TMS, ppm) 12

Figure S18: ^{29}Si NMR spectrum of 6 (C_6D_6 solution, vs ext. TMS, ppm) 12

Figure S19: ^{1}H NMR spectrum of a mixture of 7a,7b after 2h irradiation at 405 nm (C_6D_6 solution, vs ext. TMS, ppm) 13

Figure S20: ^{29}Si-INEPT-NMR spectrum of a mixture of 7a,7b after 2h irradiation at 405 nm (C_6D_6 solution, vs ext. TMS, ppm) 14

Figure S21: ^{29}Si-NMR spectrum of a mixture of 7a,7b after 2h irradiation at 405 nm (C_6D_6 solution, vs ext. TMS, ppm) 14

Figure S22: ^{13}C-NMR spectrum of a mixture of 7a,7b after 2h irradiation at 405 nm (C_6D_6 solution, vs ext. TMS, ppm) 15

Figure S23: ^{29}Si-NMR spectrum of a mixture of 7a,7b after 16h irradiation at 405 nm (C_6D_6 solution, vs ext. TMS, ppm) 15

Figure S24: ^{1}H-NMR spectrum of 9a (C_6D_6 solution, vs ext. TMS, ppm) 16

Figure S25: ^{29}Si-INEPT-NMR spectrum of 9a (C_6D_6 solution, vs ext. TMS, ppm) 16

Figure S26: ^{13}C-NMR spectrum of 9a (C_6D_6 solution, vs ext. TMS, ppm) 17

Figure S27: ^{1}H-NMR spectrum of a mixture of 8a,8b and 8c after 25 min irradiation at 405 nm (C_6D_6 solution, vs ext. TMS, ppm) 17

Figure S28: ^{29}Si-INEPT_NMR spectrum of a mixture of 8a,8b and 8c after 25 min irradiation at 405 nm (C_6D_6 solution, vs ext. TMS, ppm) 18

Figure S29: ^{13}C-NMR spectrum of a mixture of 8a,8b and 8c after 25min irradiation at 405 nm (C_6D_6 solution, vs ext. TMS, ppm) 18

Figure S30: ^{1}H-NMR spectrum of 8c after 48 h irradiation at 405 nm (C_6D_6 solution, vs ext. TMS, ppm) 19

Figure S31: ^{29}Si-INEPT-NMR spectrum of 8c after 48 h irradiation at 405 nm (C_6D_6 solution, vs ext. TMS, ppm) 19

Figure S32: ^{13}C-NMR spectrum of 8c after 48 h irradiation at 405 nm (C_6D_6 solution, vs ext. TMS, ppm) 20

Figure S33: ^{1}H-NMR spectrum of 9b after irradiation at 405 nm (C_6D_6 solution, vs ext. TMS, ppm) 20

Figure S34: ^{29}Si-INEPT-NMR spectrum 9b after irradiation at 405 nm (C_6D_6 solution, vs ext. TMS, ppm) 21

Figure S35: ^{13}C-NMR spectrum of 9b after irradiation at 405 nm (C_6D_6 solution, vs ext. TMS, ppm) 21

Figure S36: ^{1}H-NMR spectrum of 11 (C_6D_6 solution, vs ext. TMS, ppm)........ 22

Figure S37: ^{29}Si-INEPT-NMR spectrum of 11 (C_6D_6 solution, vs ext. TMS, ppm) 22

Figure S38: ^{13}C-NMR spectrum of 11 (C_6D_6 solution, vs ext. TMS, ppm) 23

Figure S39: ^{1}H-NMR spectrum of 12 (C_6D_6 solution, vs ext. TMS, ppm)........ 23

Figure S40: ^{29}Si-INEPT-NMR spectrum of 12 (C_6D_6 solution, vs ext. TMS, ppm) 24

Figure S41: ^{13}C-NMR spectrum of 12 (C_6D_6 solution, vs ext. TMS, ppm) 24

X-ray Crystallography........ 25

Table S1. Crystallographic data and details of measurements for compounds 3, 4, 7b, 11 and 12. 25

Figure S42 Molecular orbitals involved in S1 excitation for compounds 3 to 6, drawn with contour values of 0.02 a.u........ 27

Analytical Section

NMR-Spectroscopy

Figure S1: ^{1}H NMR spectrum of **1** (C_6D_6 solution, vs ext. TMS, ppm)

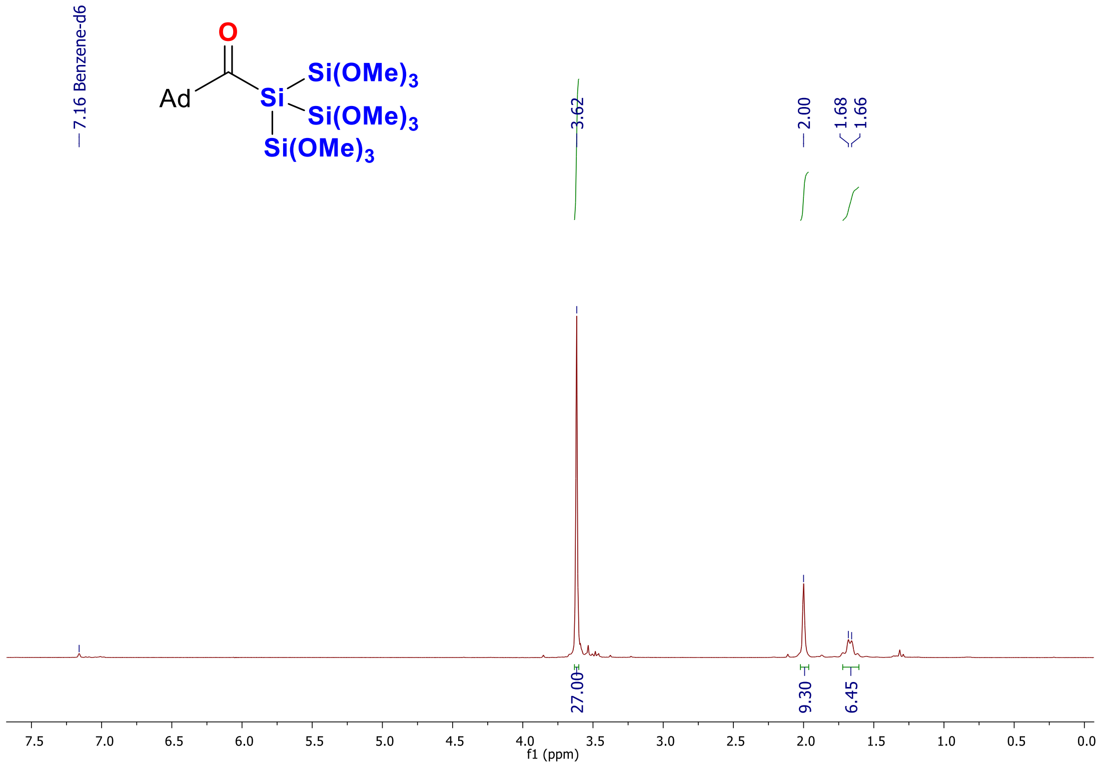

Figure S2: ^{13}C NMR spectrum of **1** (C_6D_6 solution, vs ext. TMS, ppm)

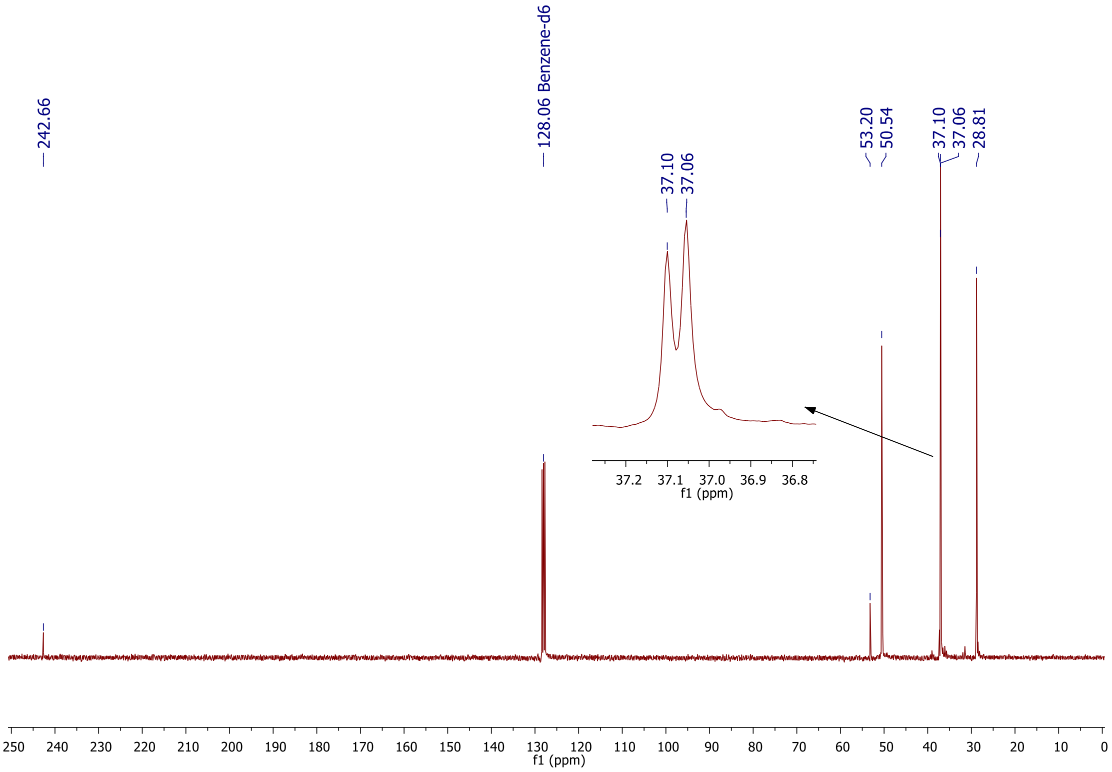

Figure S3: ^{29}Si NMR spectrum of **1** (C_6D_6 solution, vs ext. TMS, ppm)

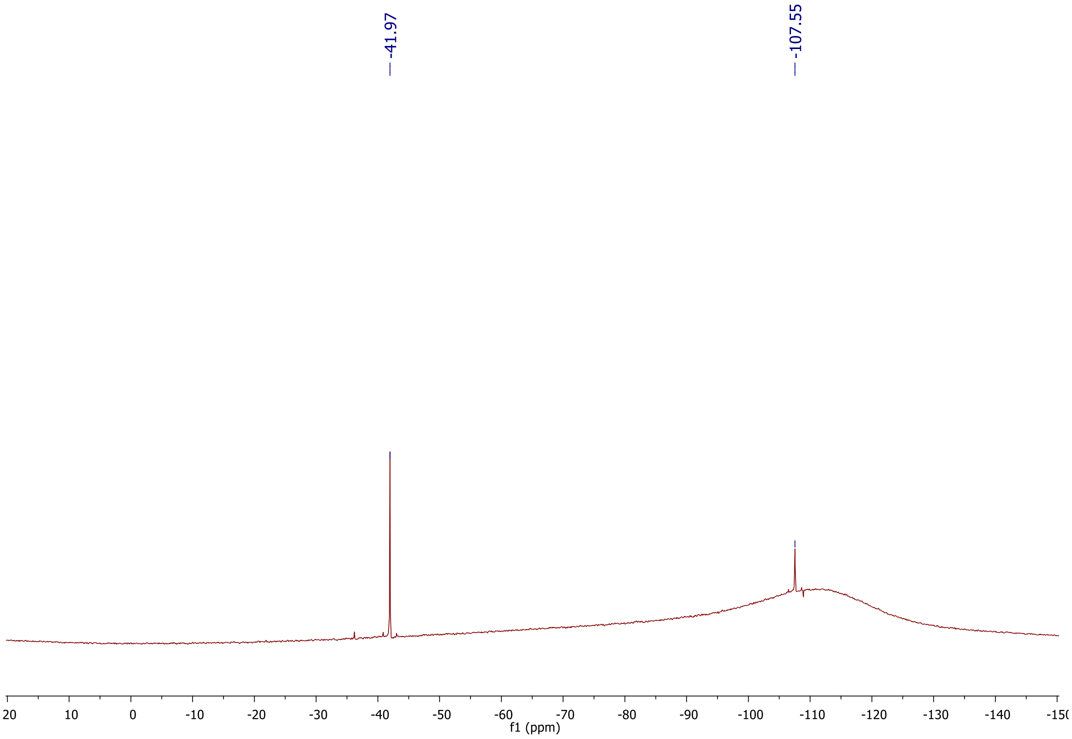

Figure S4: ^{1}H NMR spectrum of **2** (C_6D_6 solution, vs ext. TMS, ppm)

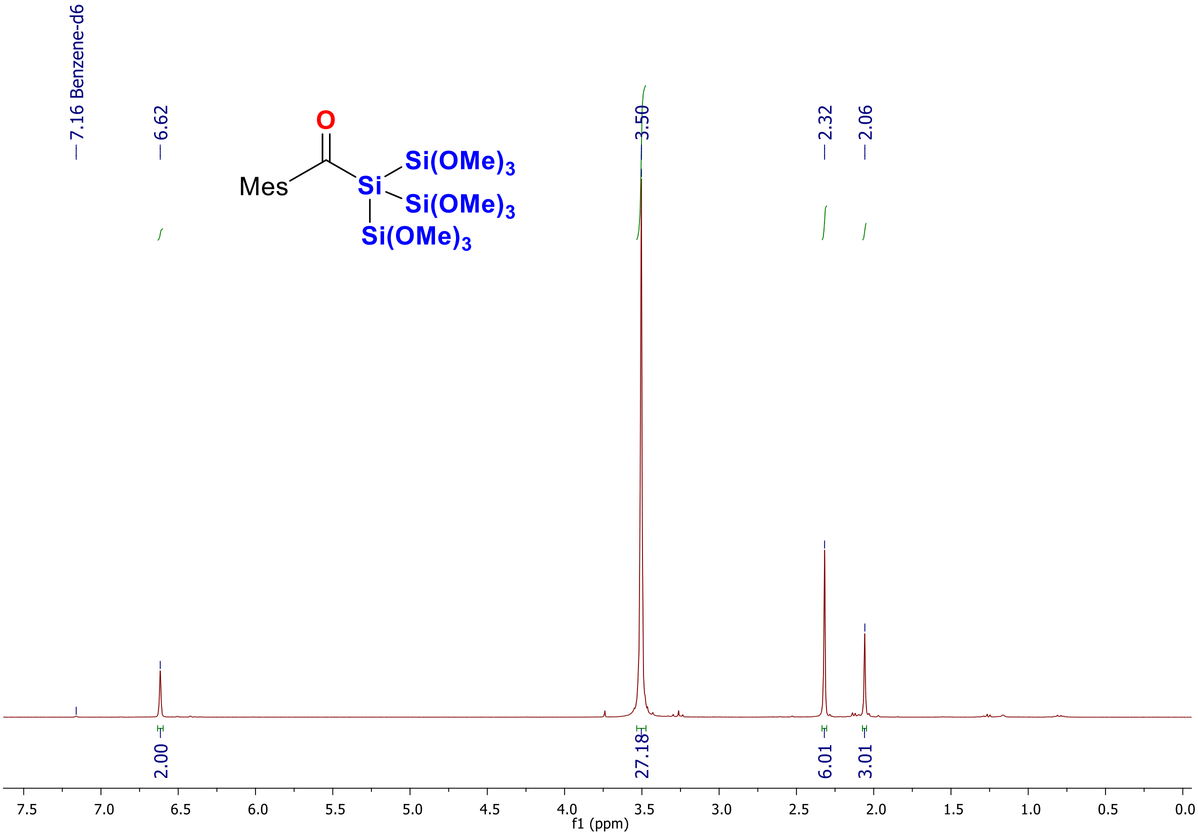

Figure S5: ^{13}C NMR spectrum of **2** (C_6D_6 solution, vs ext. TMS, ppm)

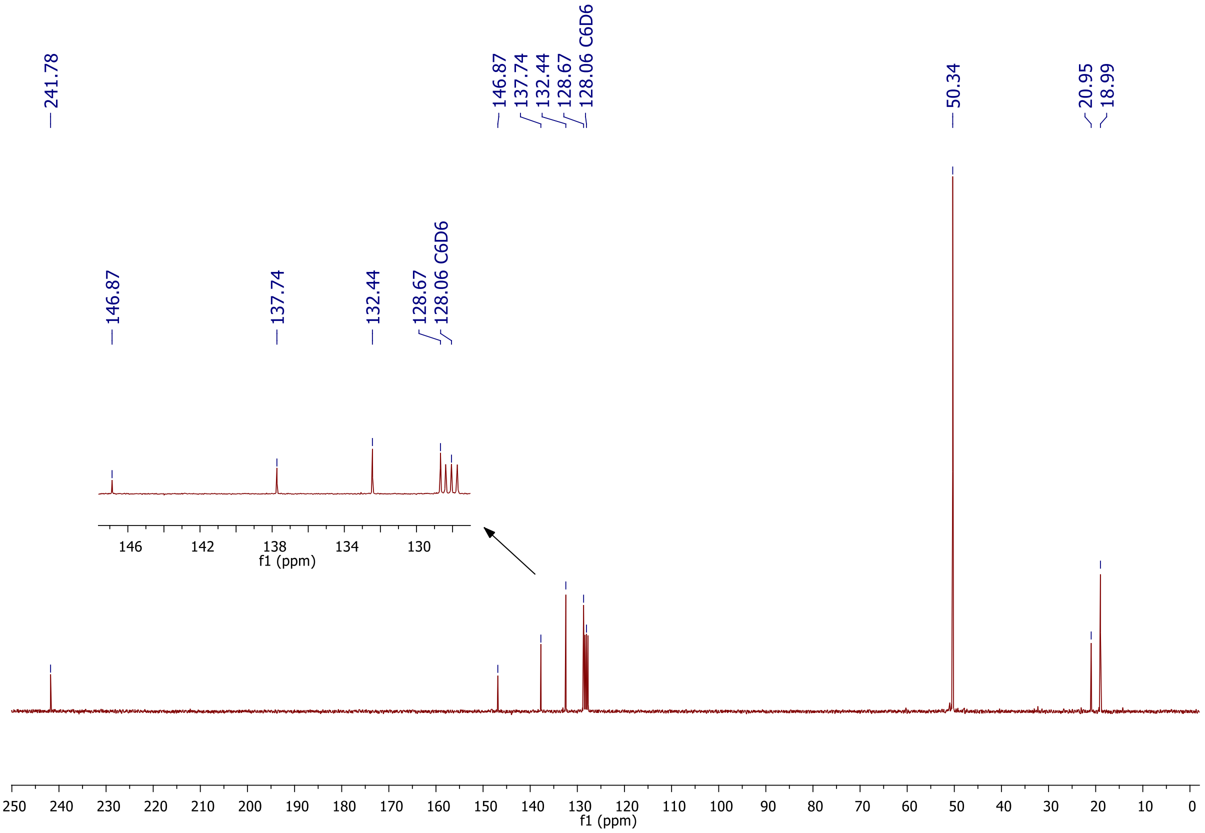

Figure S6: ^{29}Si NMR spectrum of **2** (C_6D_6 solution, vs ext. TMS, ppm)

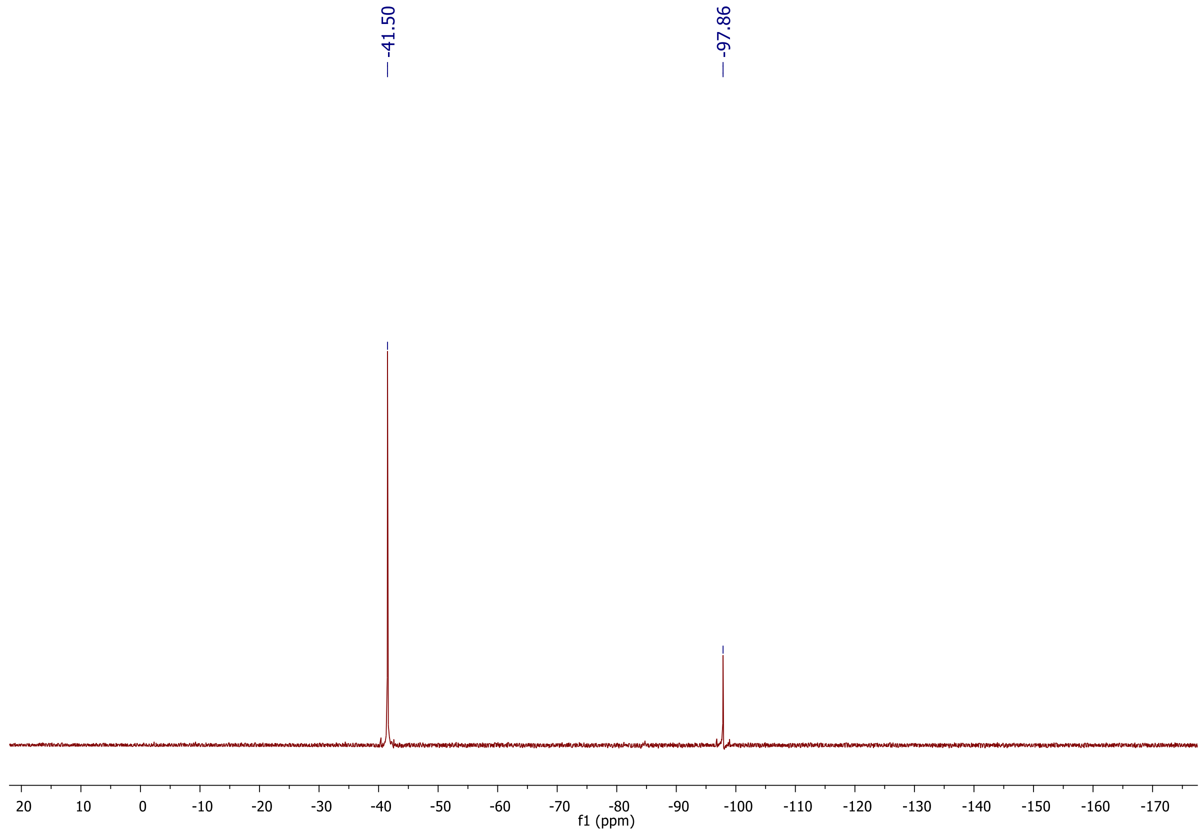

Figure S7: ^{1}H NMR spectrum of **3** ($CDCl_3$ solution, vs ext. TMS, ppm)

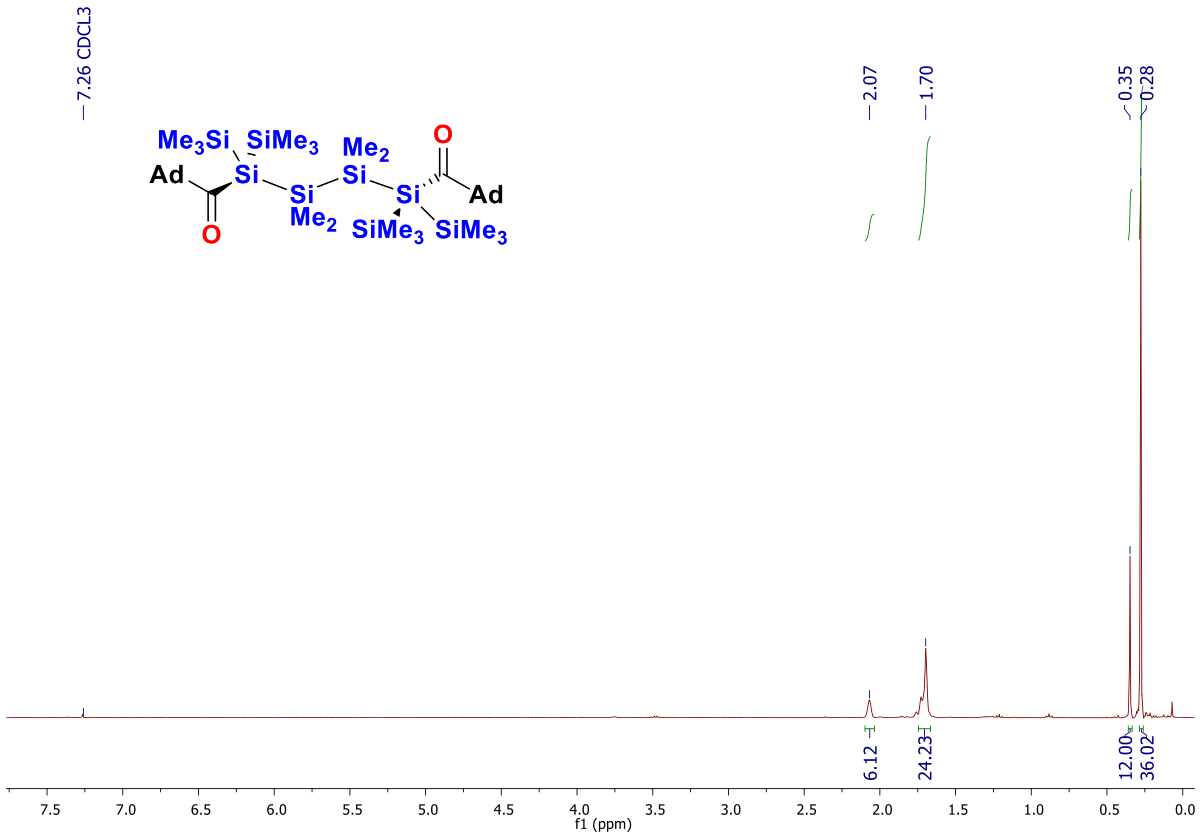

Figure S8: ^{13}C NMR spectrum of **3** ($CDCl_3$ solution, vs ext. TMS, ppm)

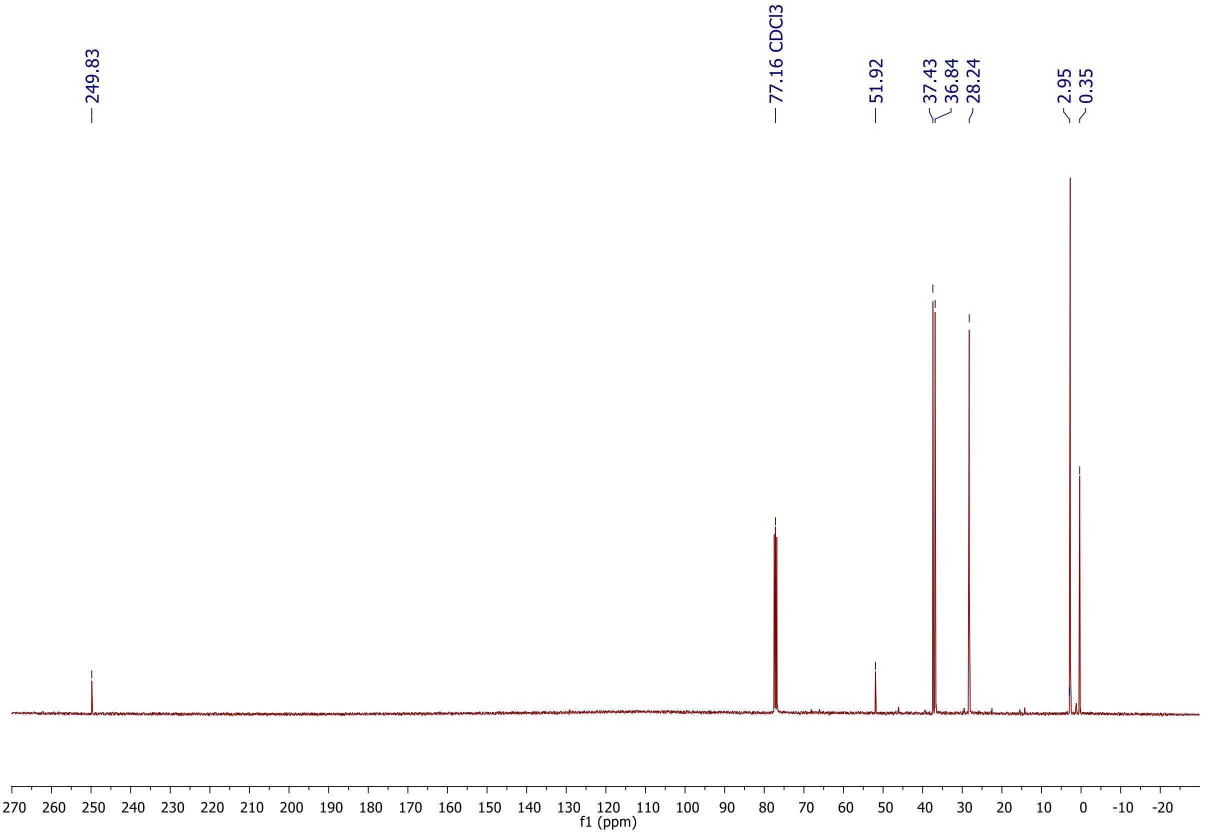

Figure S9: ^{29}Si NMR spectrum of **3** ($CDCl_3$ solution, vs ext. TMS, ppm)

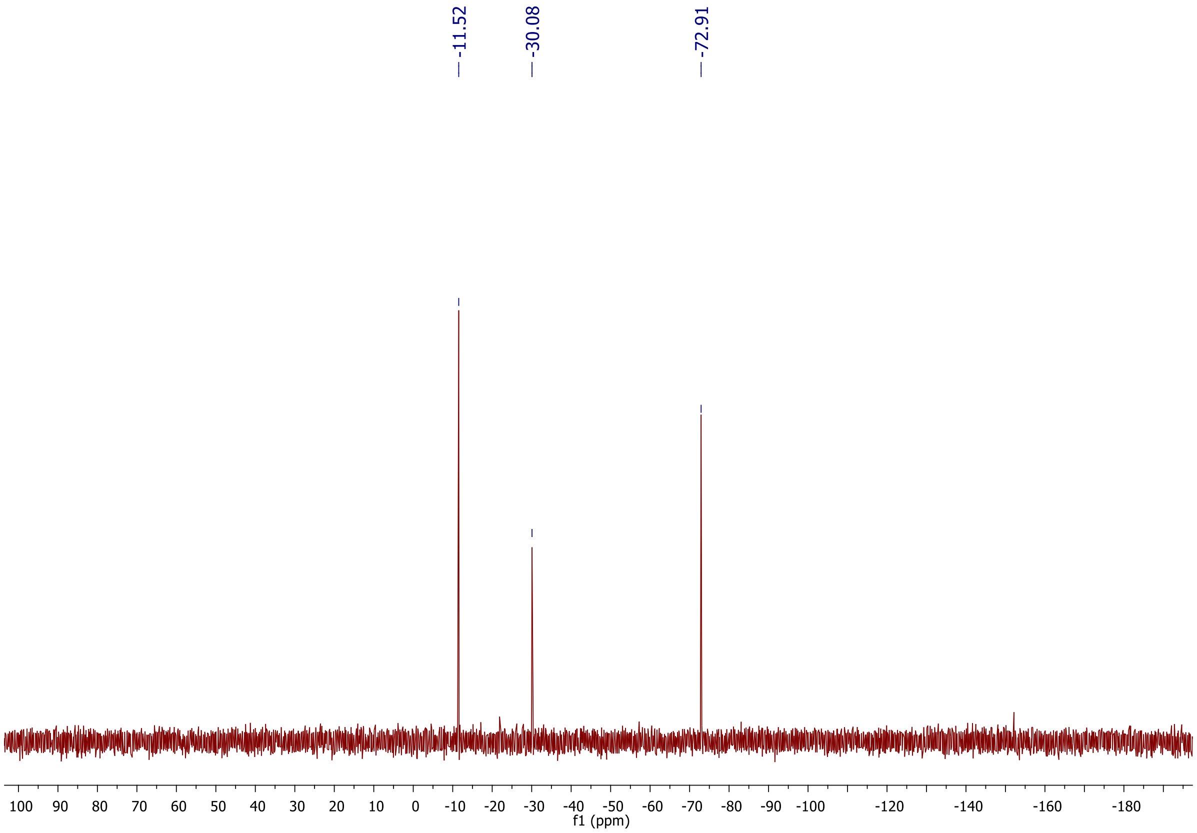

Figure S10: ^{1}H NMR spectrum of **4** (C_6D_6 solution, vs ext. TMS, ppm)

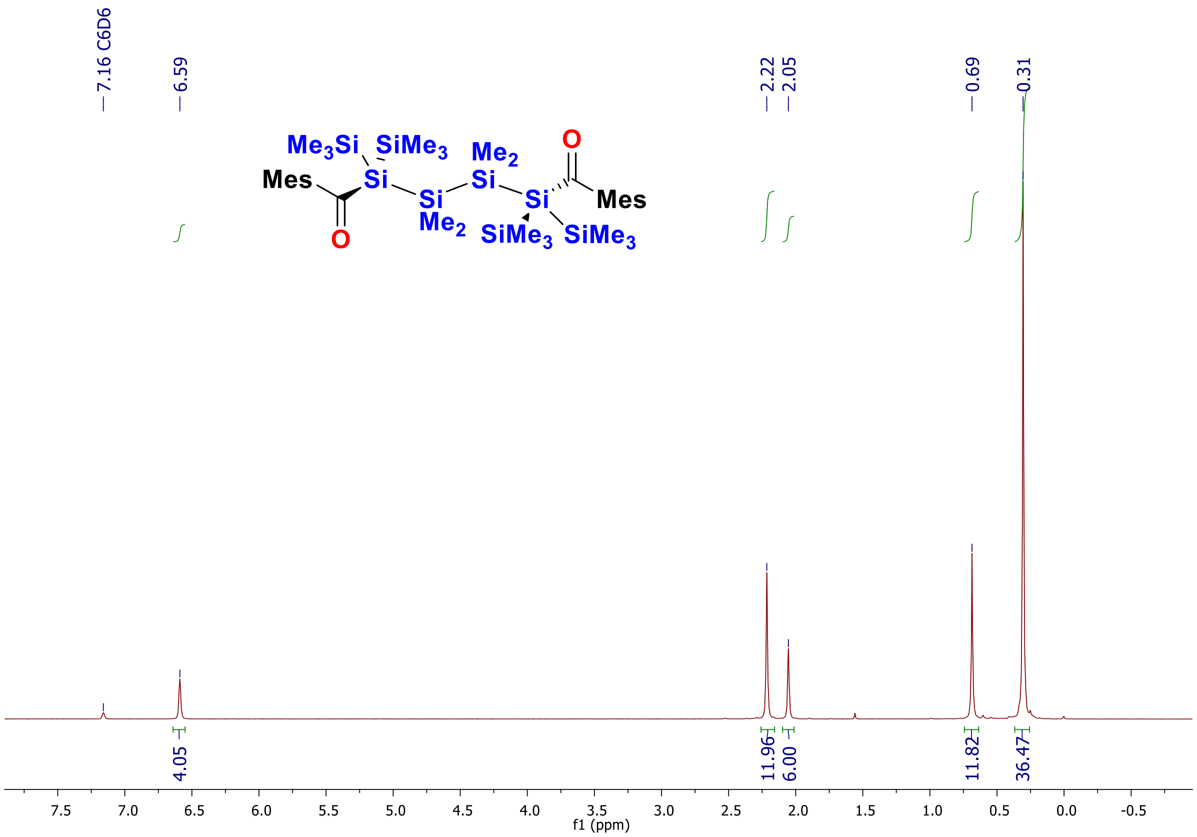

Figure S11: ^{13}C NMR spectra of **4** (C_6D_6 solution, vs ext. TMS, ppm)

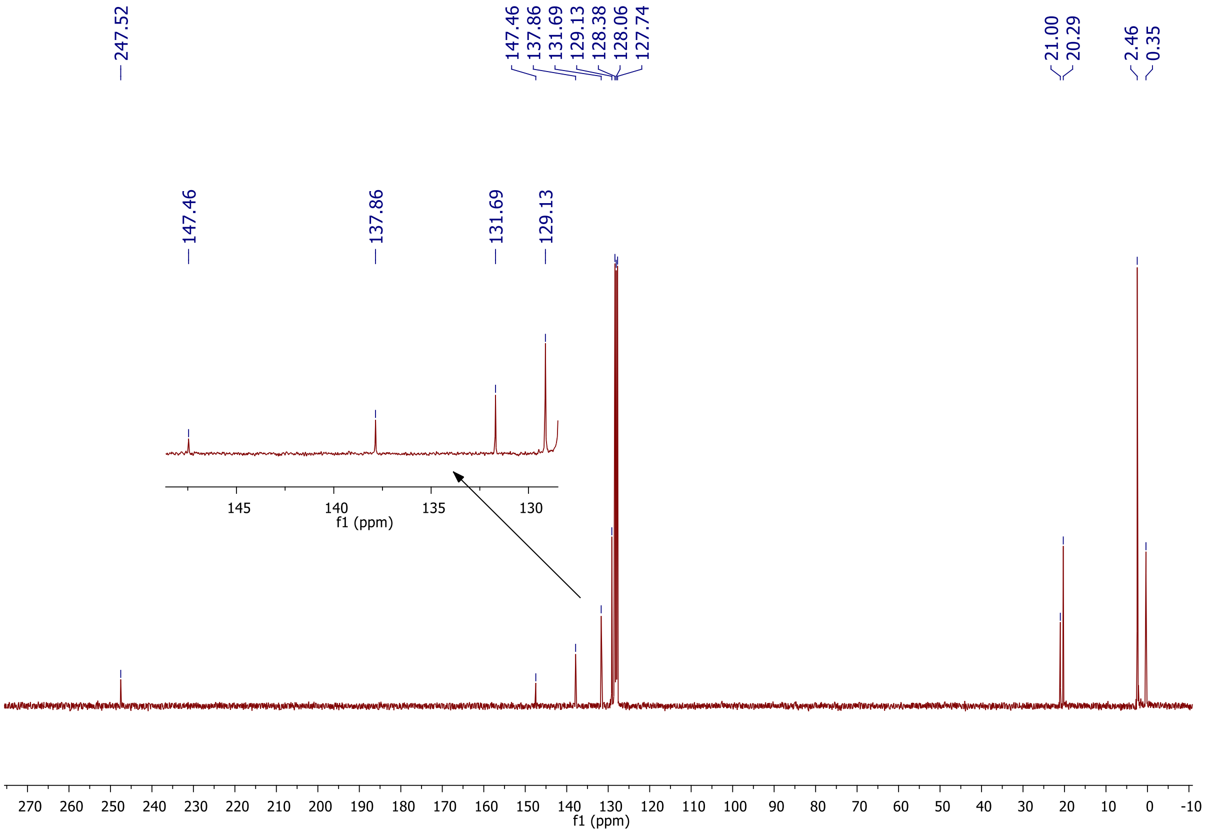

Figure S12: ^{29}Si NMR spectra of **4** (C_6D_6 solution, vs ext. TMS, ppm)

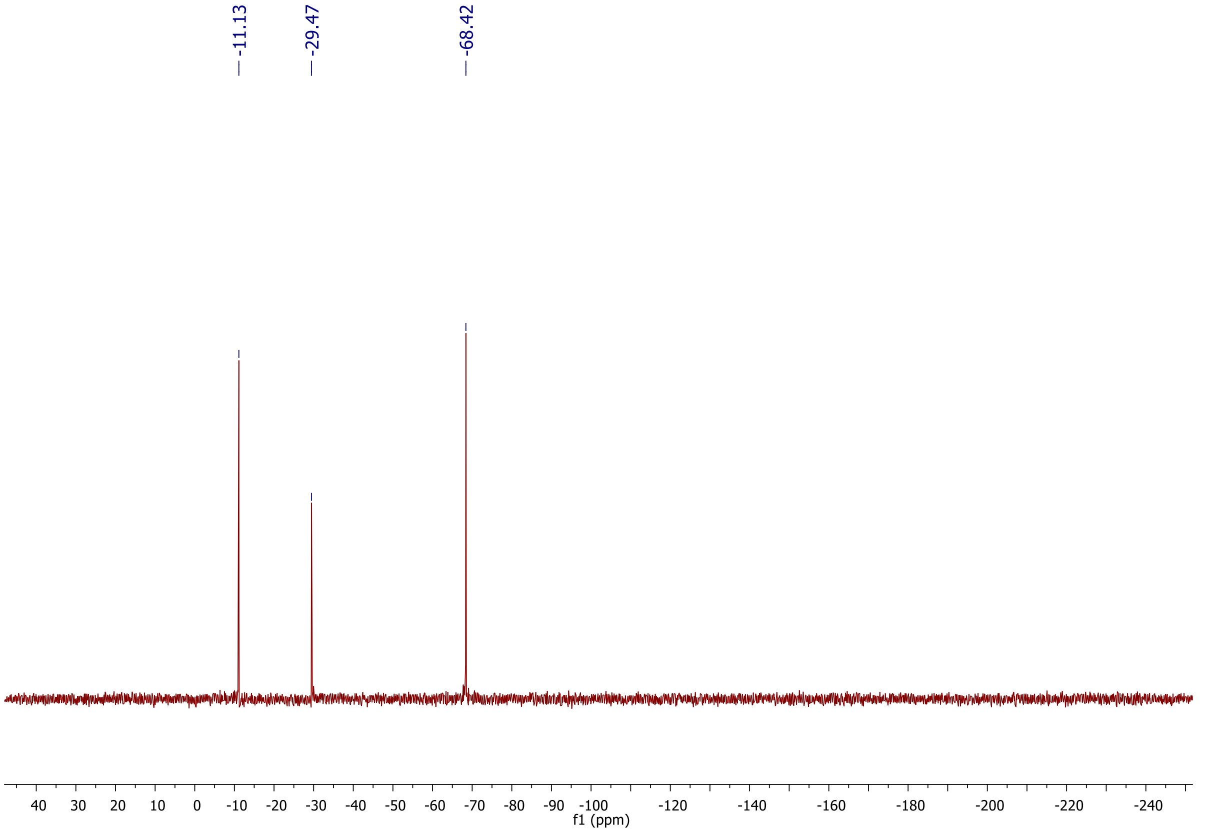

Figure S13: ^{1}H NMR spectra of **5** (C_6D_6 solution, vs ext. TMS, ppm)

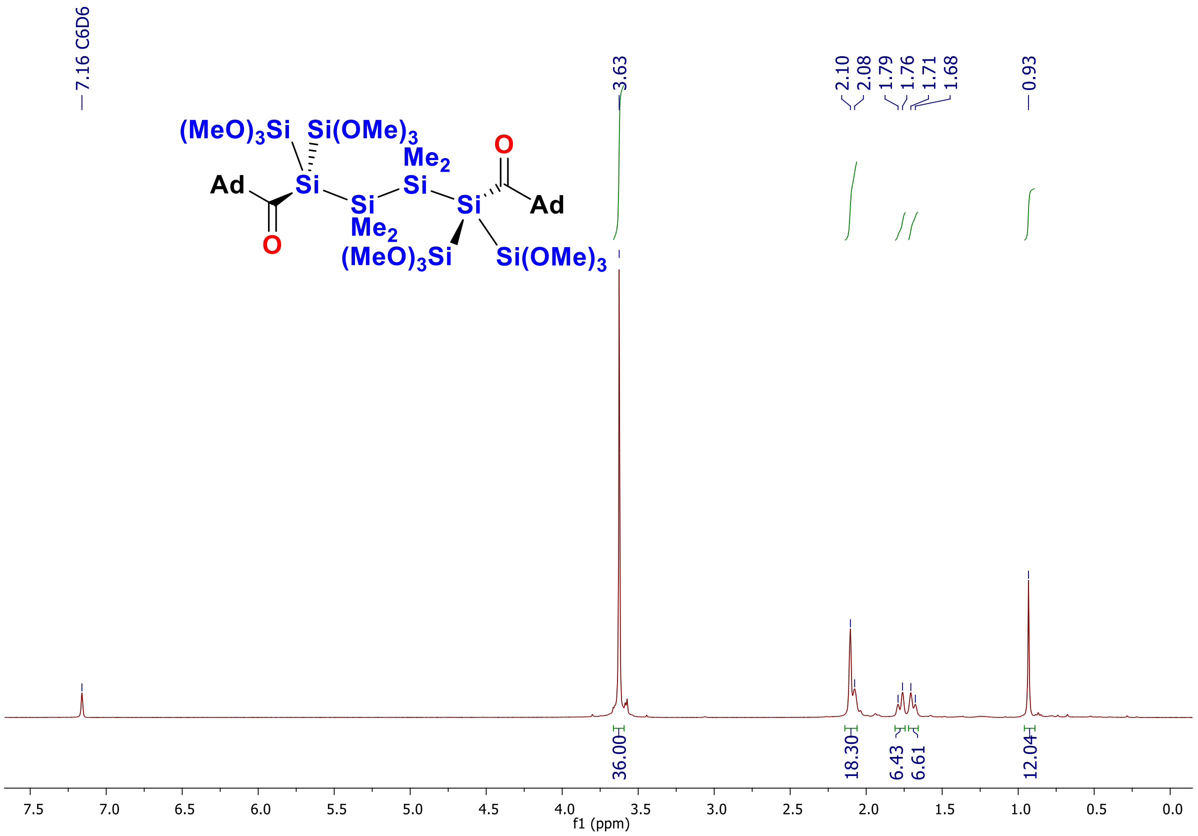

Figure S14: ^{13}C NMR spectra of **5** (C_6D_6 solution, vs ext. TMS, ppm)

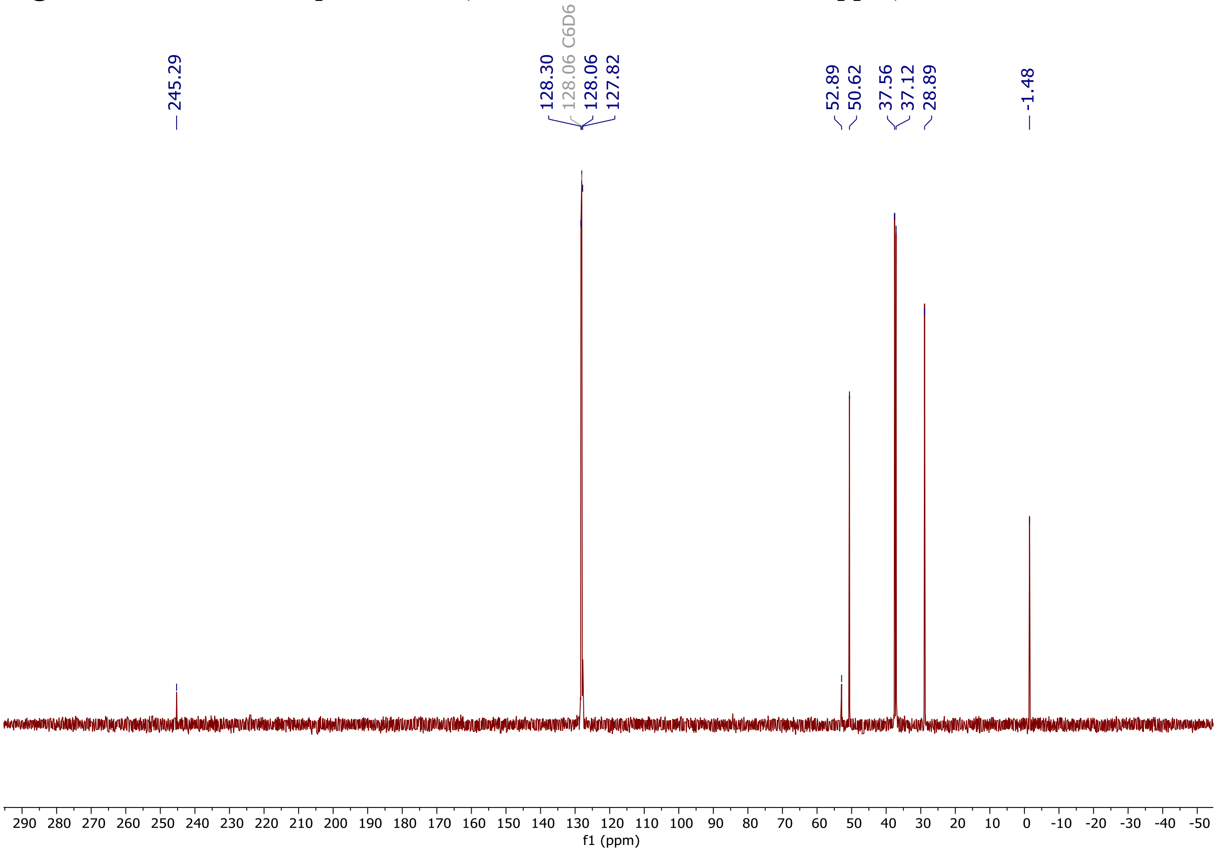

Figure S15: ^{29}Si NMR spectra of 5 (C_6D_6 solution, vs ext. TMS, ppm)

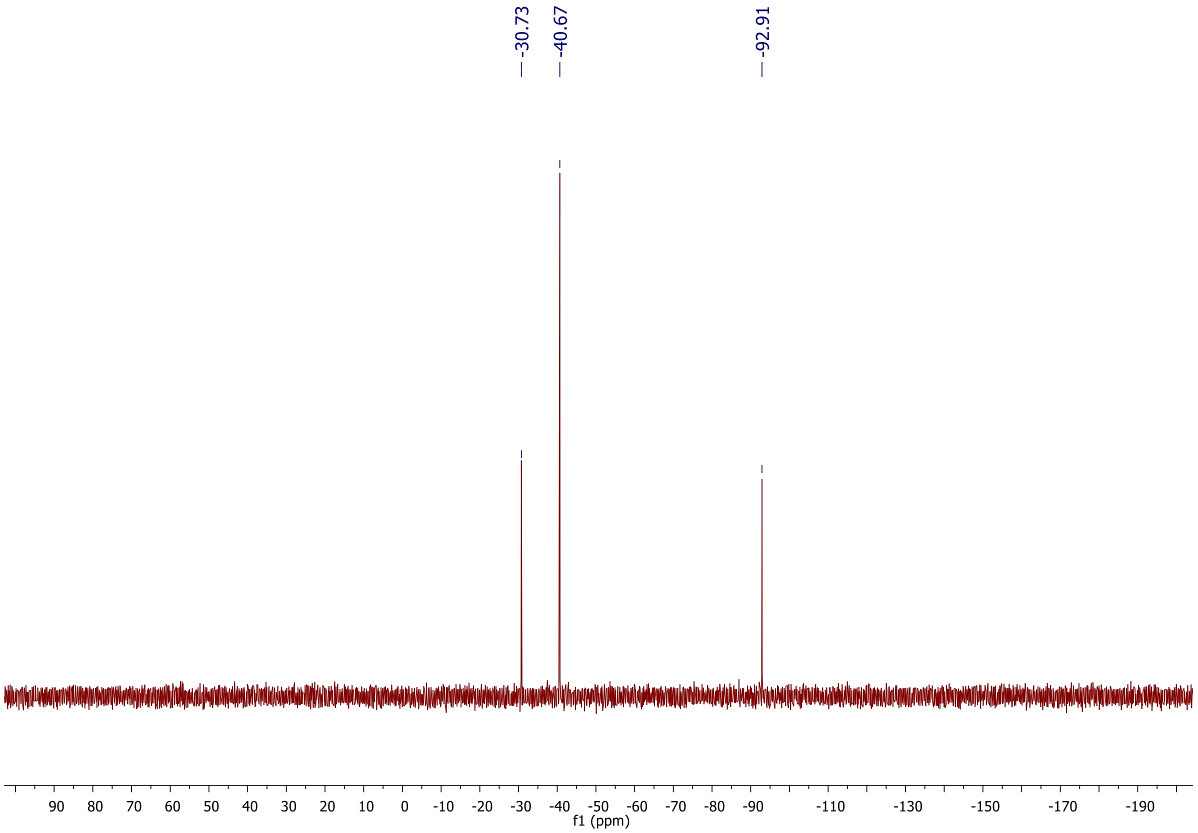

Figure S16: ^{1}H NMR spectrum of 6 (C_6D_6 solution, vs ext. TMS, ppm)

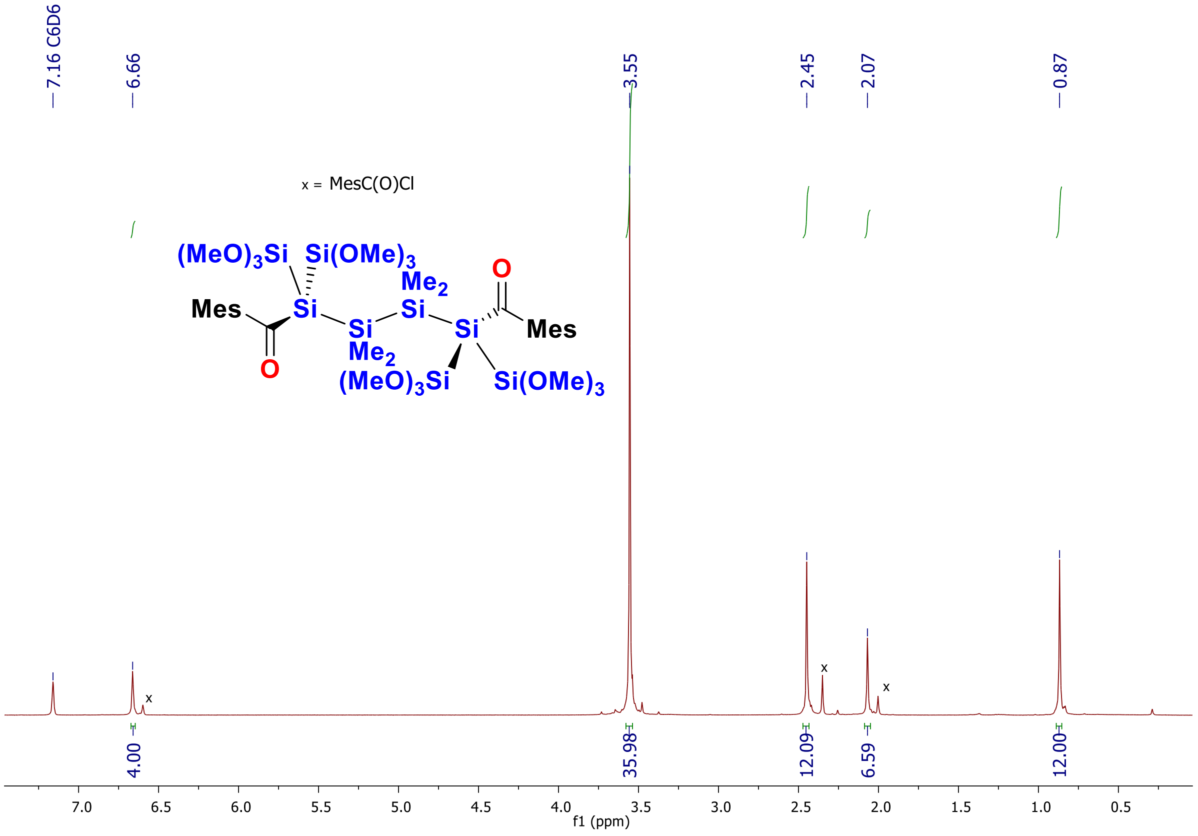

Figure S17: ^{13}C-NMR spectrum of **6** (C_6D_6 solution, vs ext. TMS, ppm)

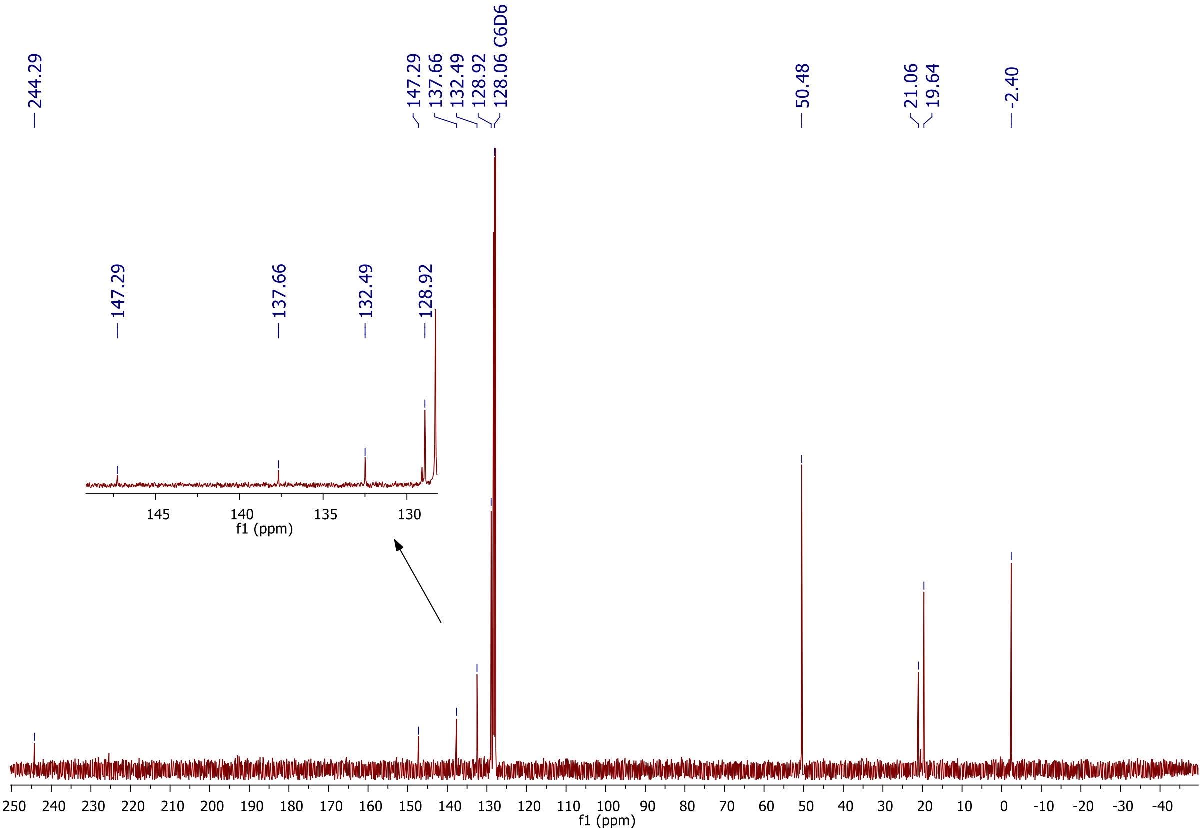

Figure S18: ^{29}Si NMR spectrum of **6** (C_6D_6 solution, vs ext. TMS, ppm)

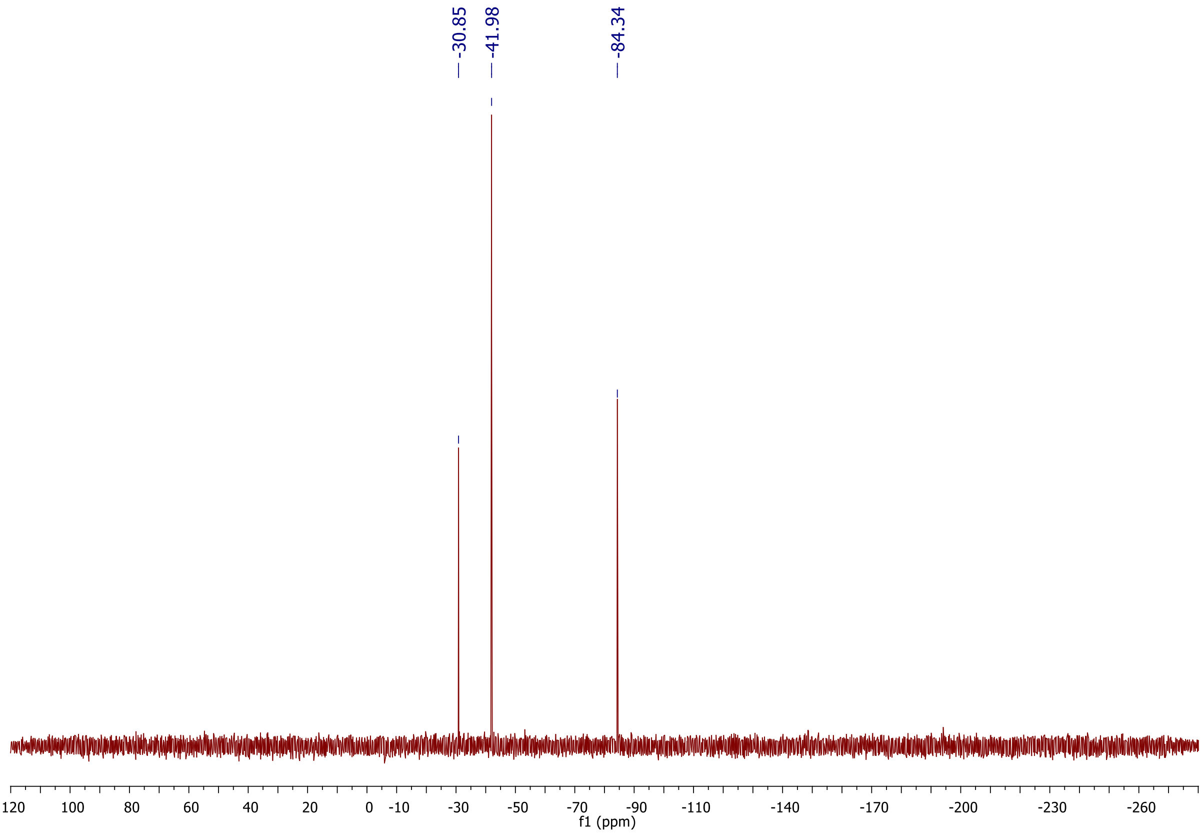

Figure S19: ^{1}H NMR spectrum of a mixture of 7a,7b after 2h irradiation at 405 nm (C_6D_6 solution, vs ext. TMS, ppm)

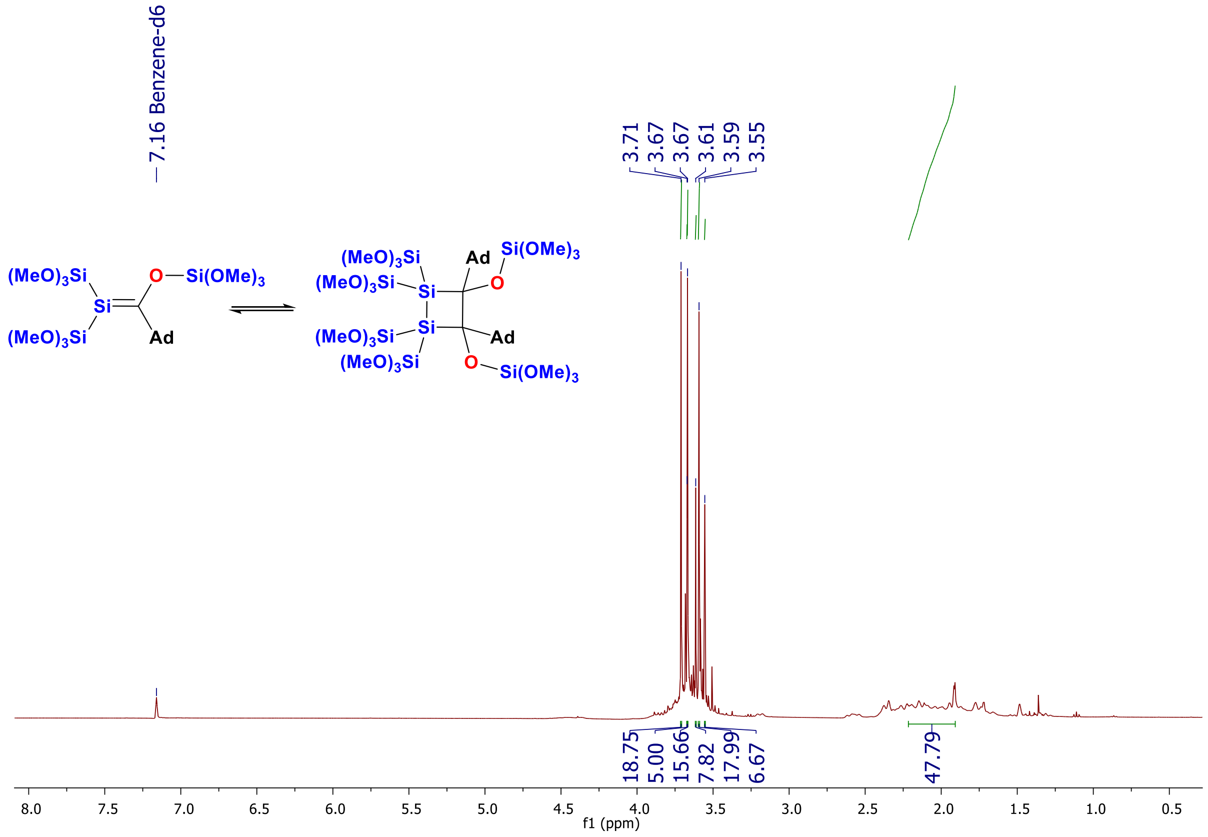

Figure S20: ^{29}Si-INEPT-NMR spectrum of a mixture of **7a,7b** after 2h irradiation at 405 nm (C_6D_6 solution, vs ext. TMS, ppm)

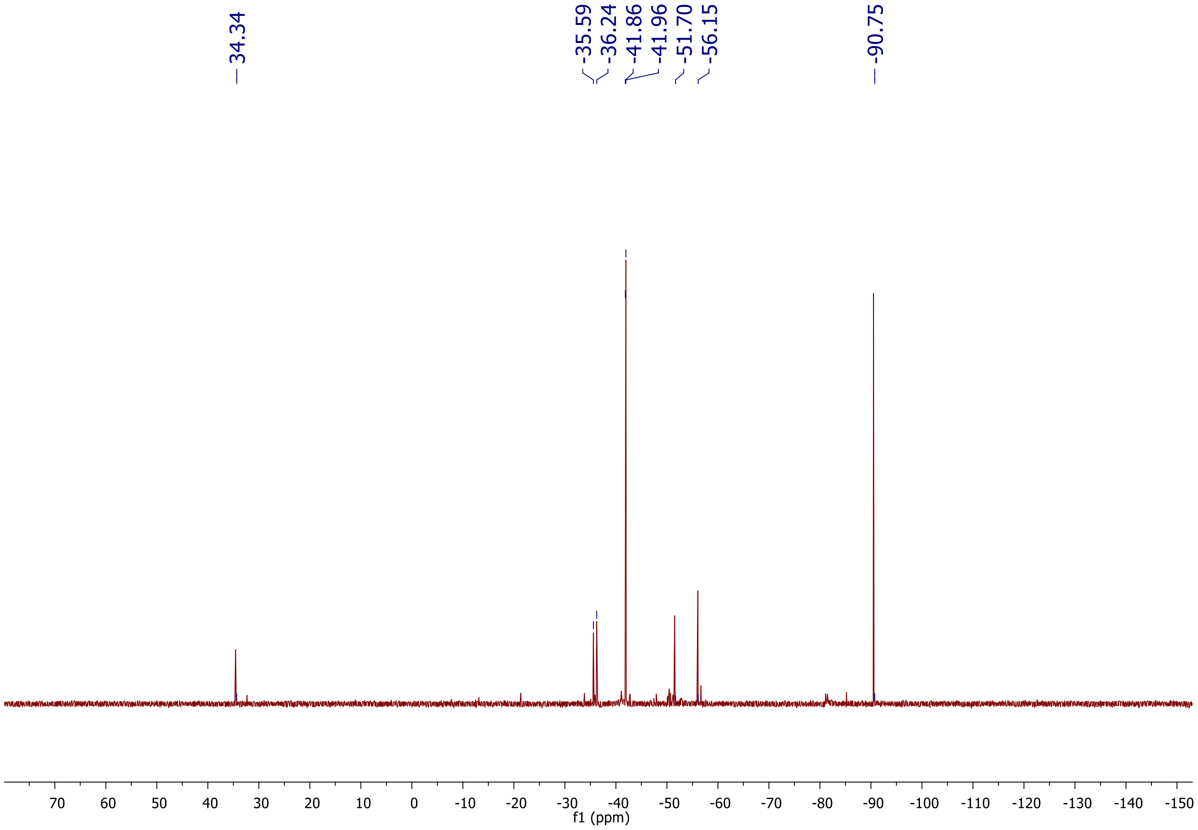

Figure S21: ^{29}Si-NMR spectrum of a mixture of **7a,7b** after 2h irradiation at 405 nm (C_6D_6 solution, vs ext. TMS, ppm)

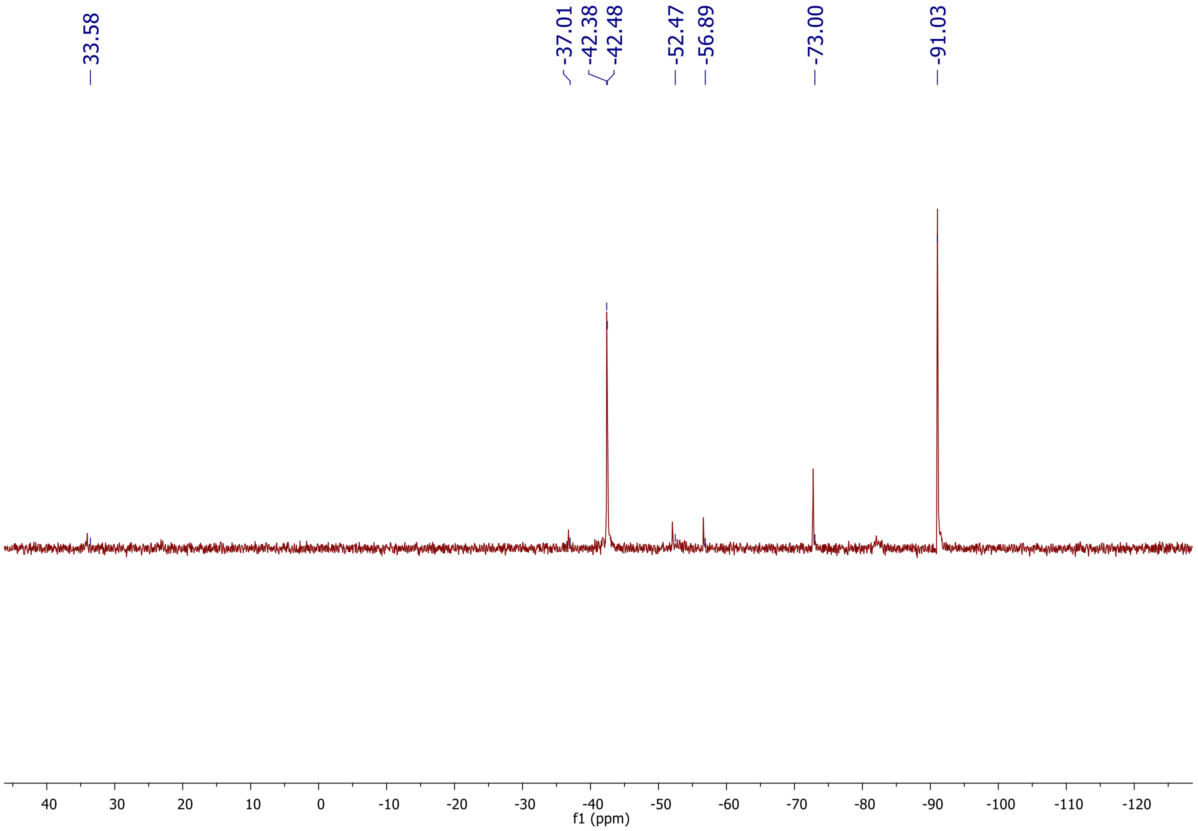

Figure S22: ^{13}C-NMR spectrum of a mixture of **7a,7b** after 2h irradiation at 405 nm (C_6D_6 solution, vs ext. TMS, ppm)

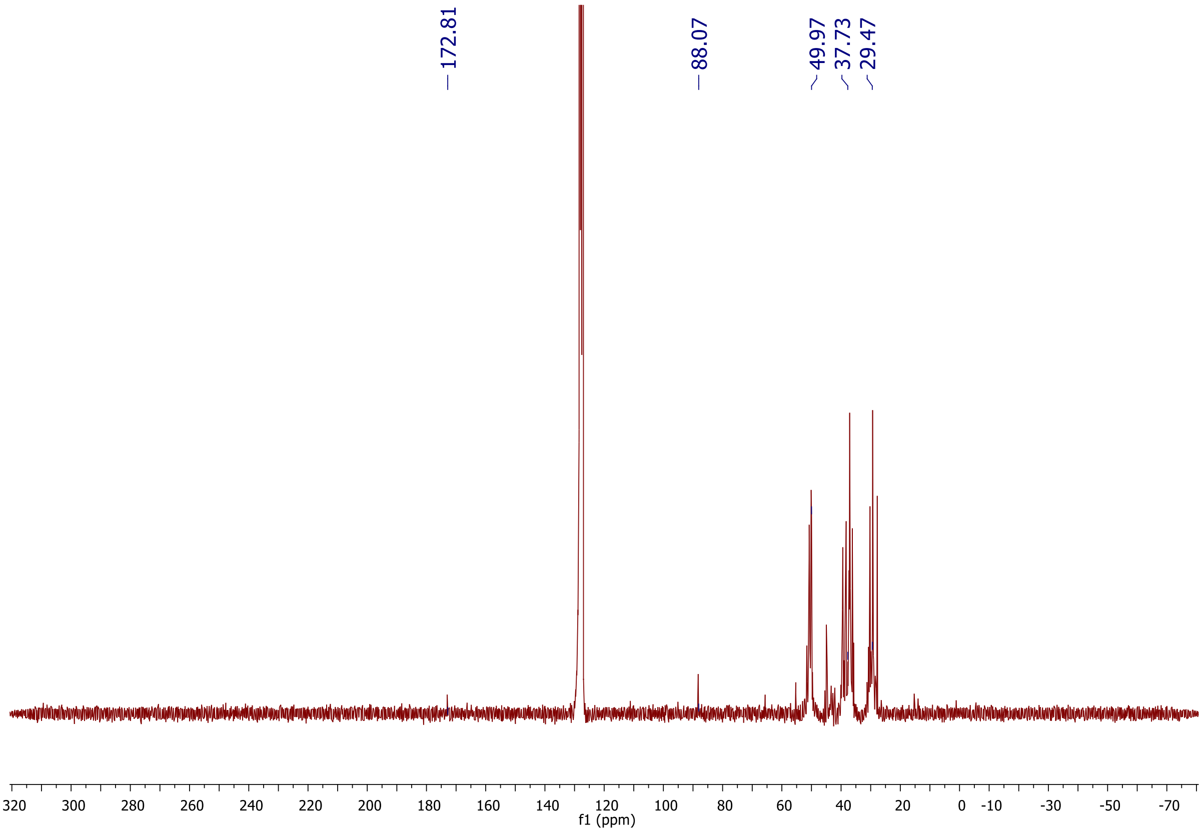

Figure S23: ^{29}Si-NMR spectrum of a mixture of **7a,7b** after 16h irradiation at 405 nm (C_6D_6 solution, vs ext. TMS, ppm)

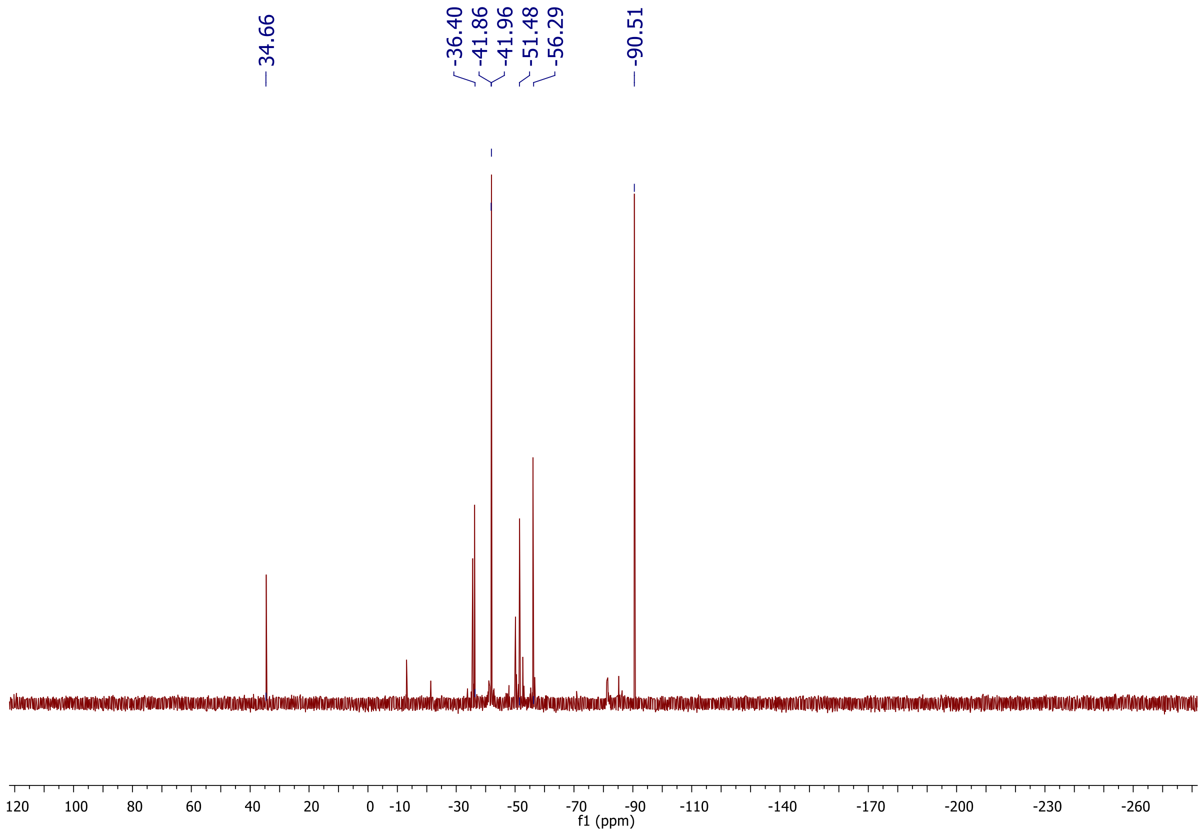

Figure S24: ^{1}H-NMR spectrum of **9a** (C_6D_6 solution, vs ext. TMS, ppm)

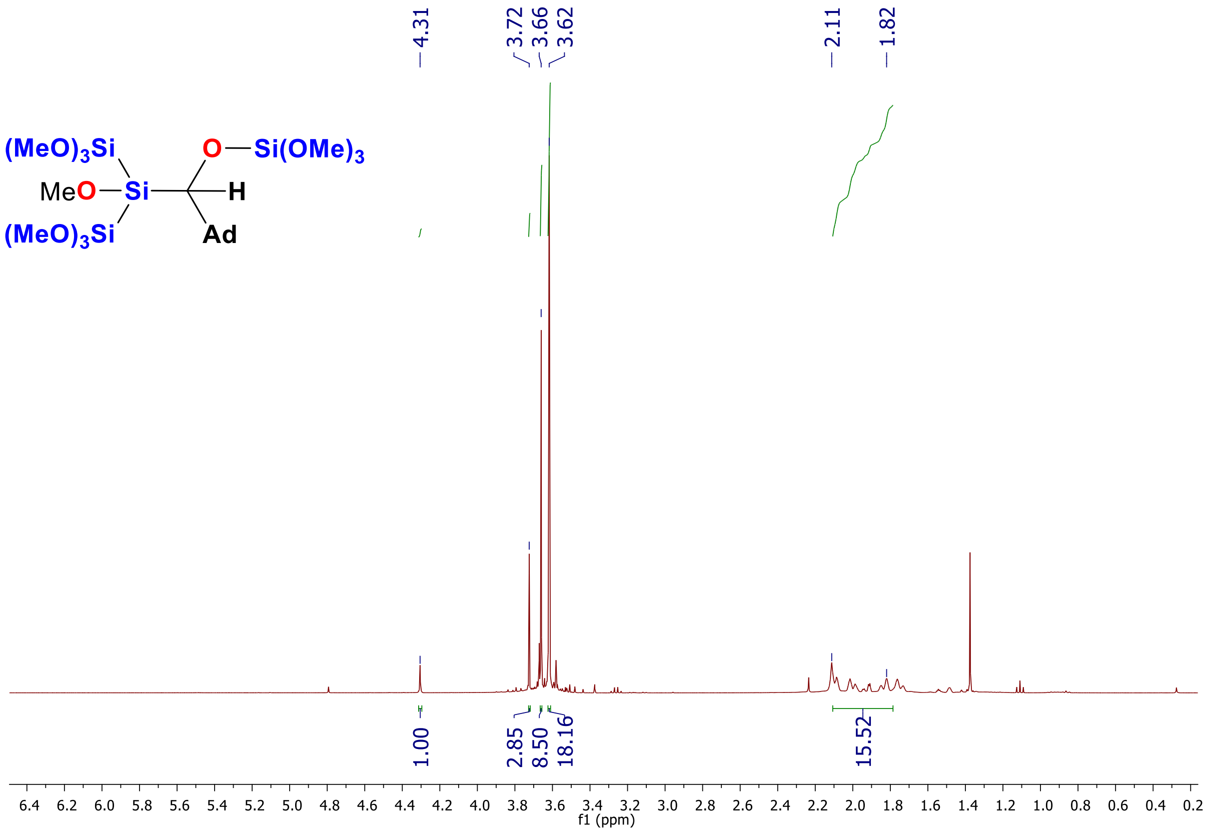

Figure S25: ^{29}Si-INEPT-NMR spectrum of **9a** (C_6D_6 solution, vs ext. TMS, ppm)

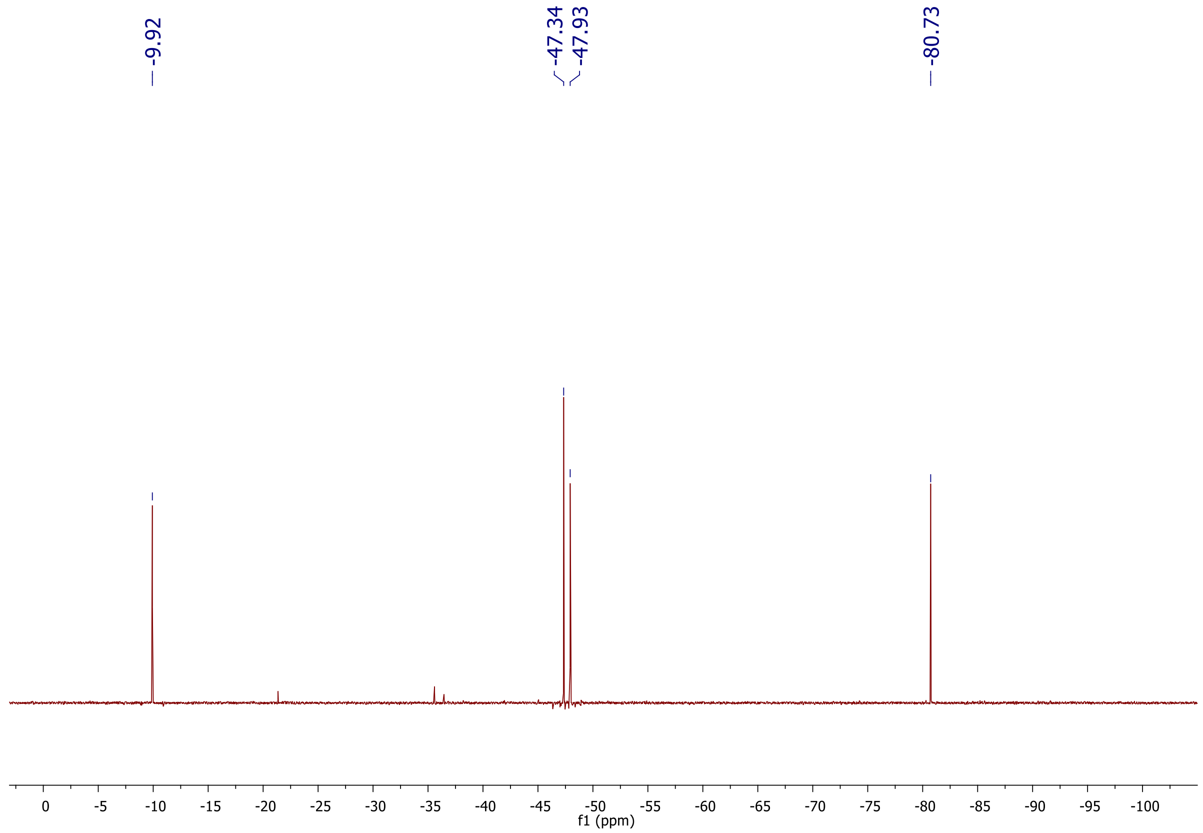

Figure S26: ^{13}C-NMR spectrum of **9a** (C_6D_6 solution, vs ext. TMS, ppm)

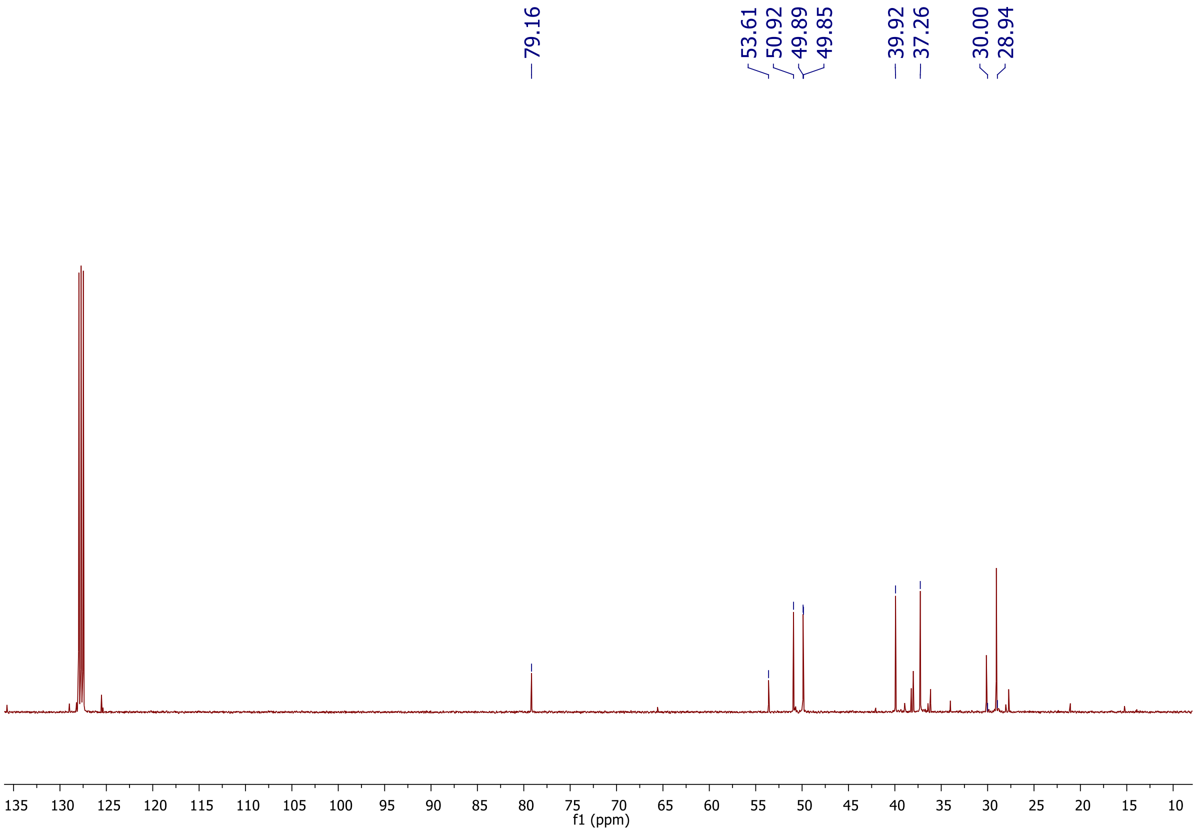

Figure S27: ^{1}H-NMR spectrum of a mixture of **8a**,**8b** and **8c** after 25 min irradiation at 405 nm (C_6D_6 solution, vs ext. TMS, ppm)

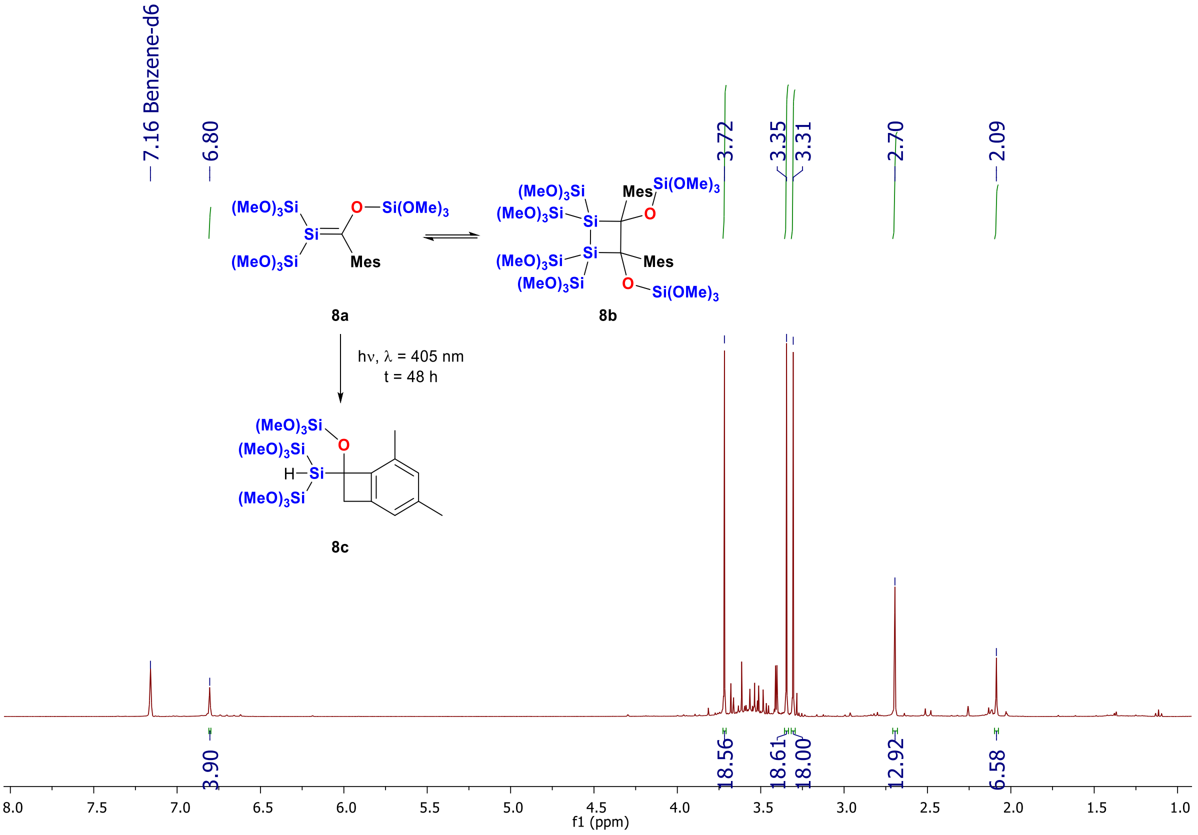

Figure S28: ^{29}Si-INEPT_NMR spectrum of a mixture of **8a,8b and 8c** after 25 min irradiation at 405 nm (C_6D_6 solution, vs ext. TMS, ppm)

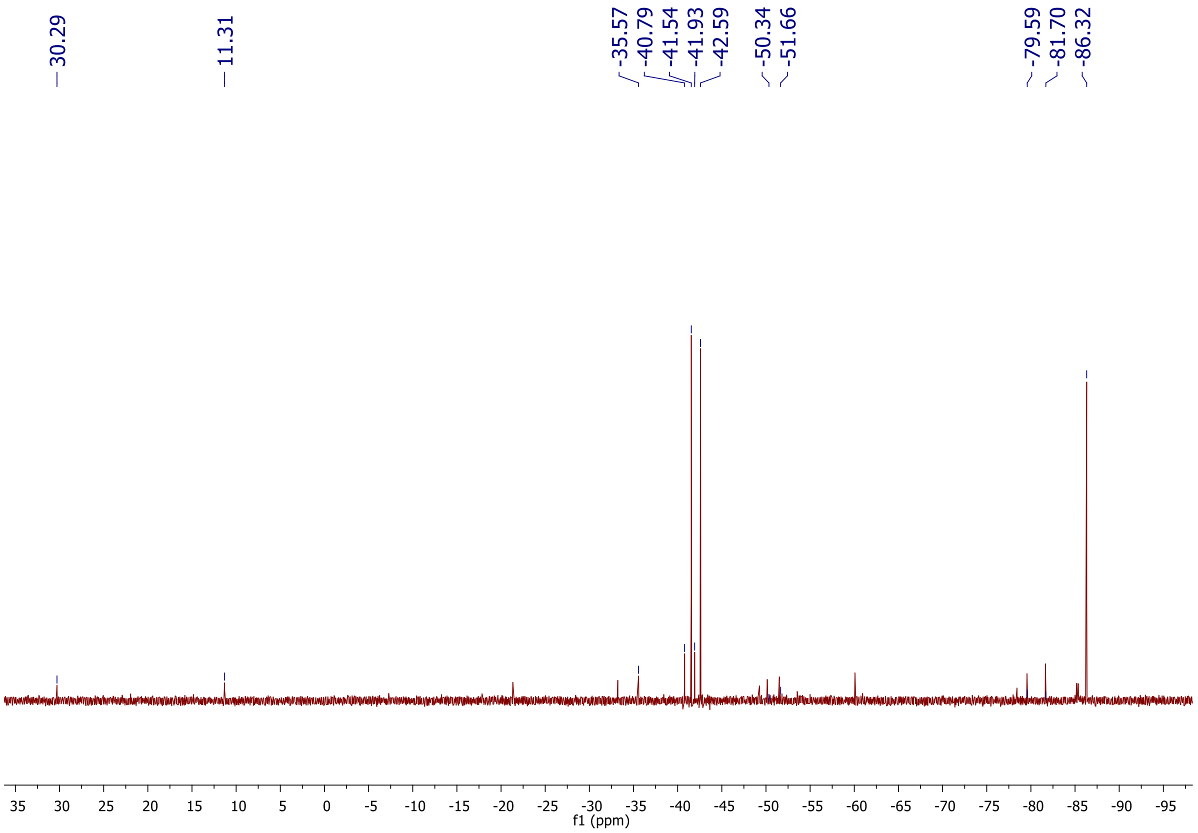

Figure S29: ^{13}C-NMR spectrum of a mixture of **8a,8b** and **8c** after 25min irradiation at 405 nm (C_6D_6 solution, vs ext. TMS, ppm)

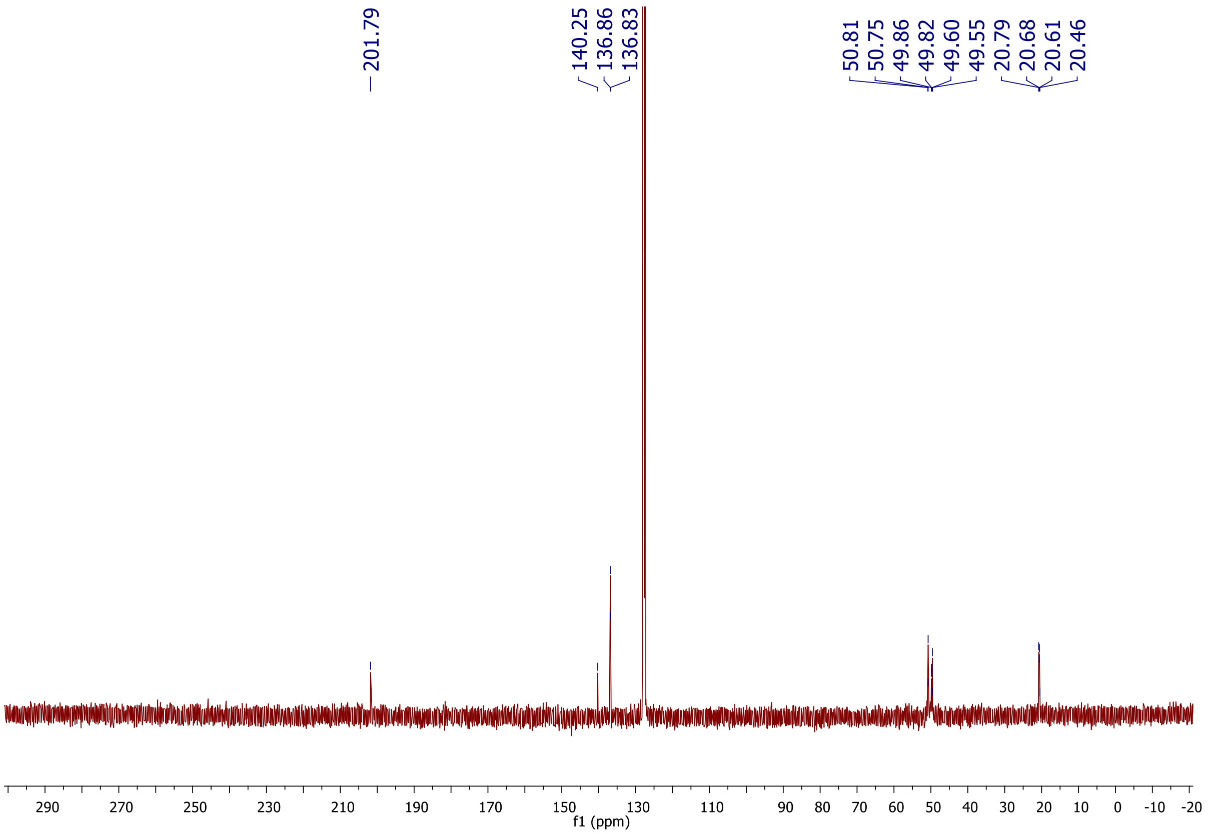

Figure S30: ^{1}H-NMR spectrum of **8c** after 48 h irradiation at 405 nm (C_6D_6 solution, vs ext. TMS, ppm)

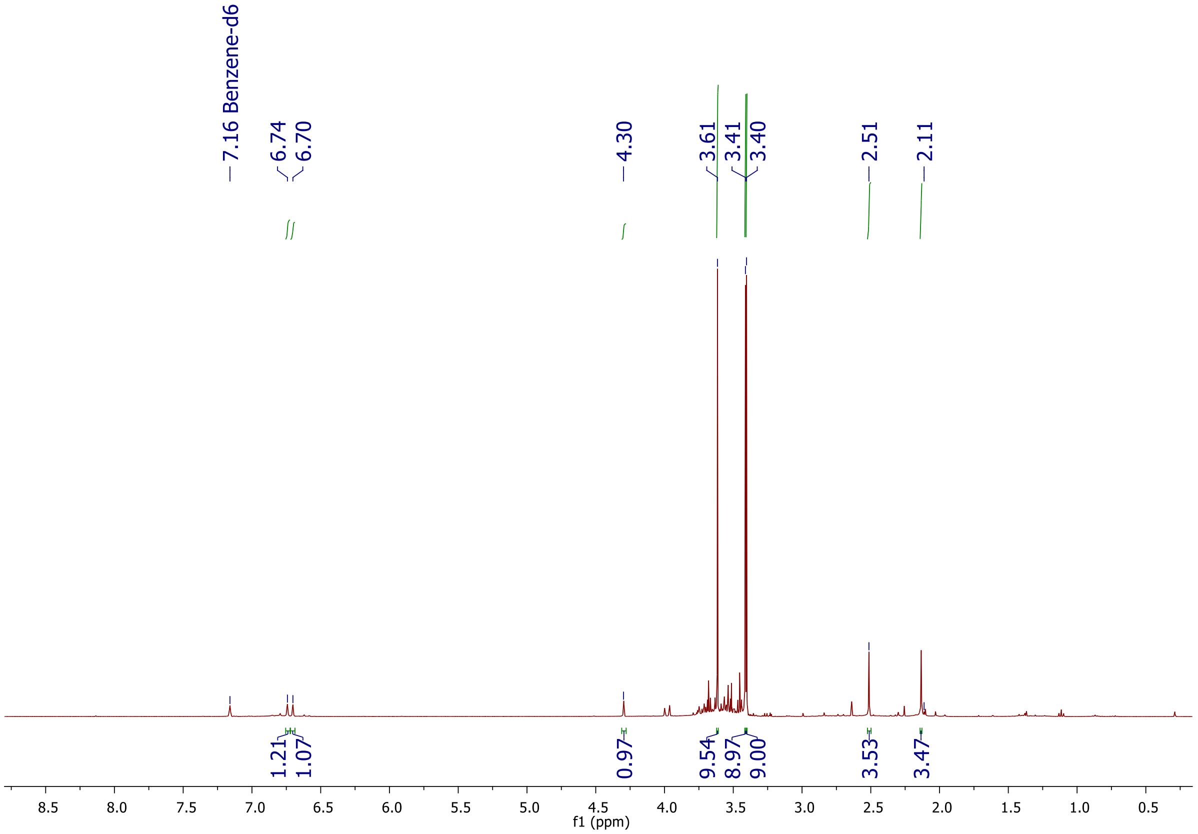

Figure S31: ^{29}Si-INEPT-NMR spectrum of **8c** after 48 h irradiation at 405 nm (C_6D_6 solution, vs ext. TMS, ppm)

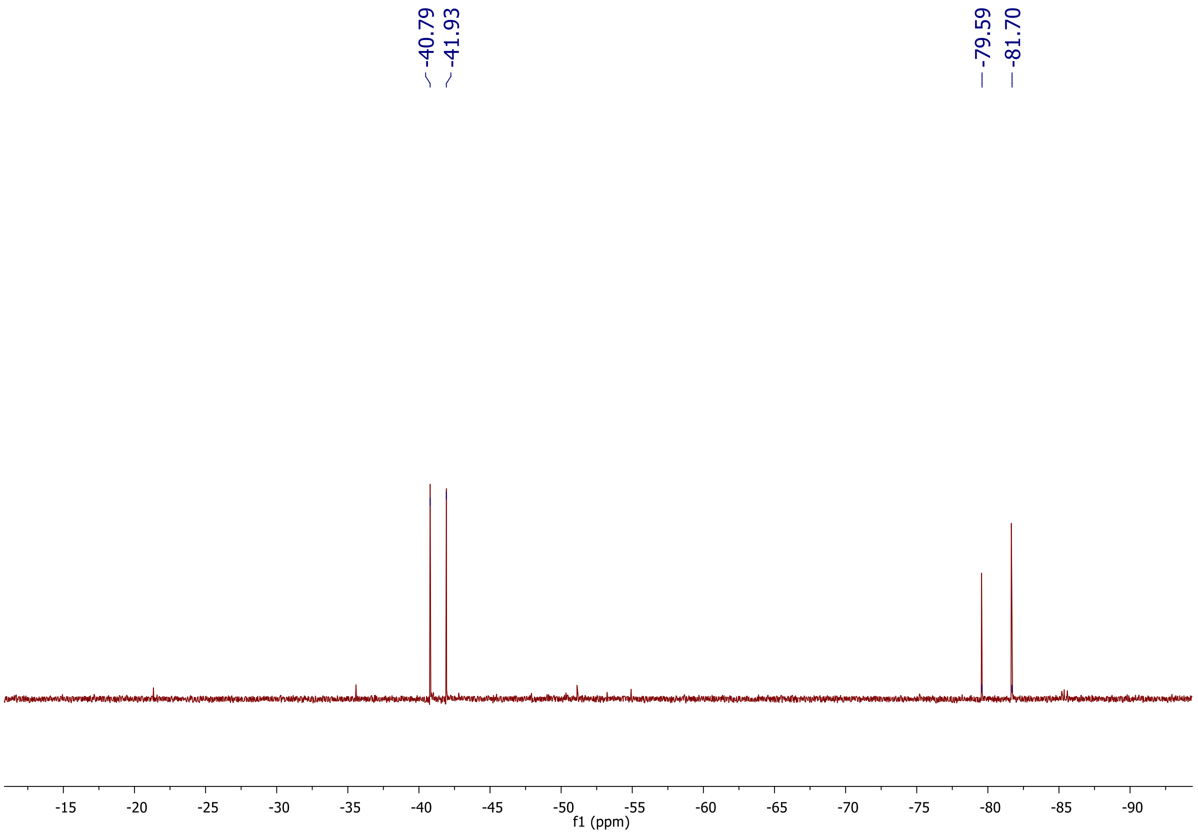

Figure S32: ^{13}C-NMR spectrum of **8c** after 48 h irradiation at 405 nm (C_6D_6 solution, vs ext. TMS, ppm)

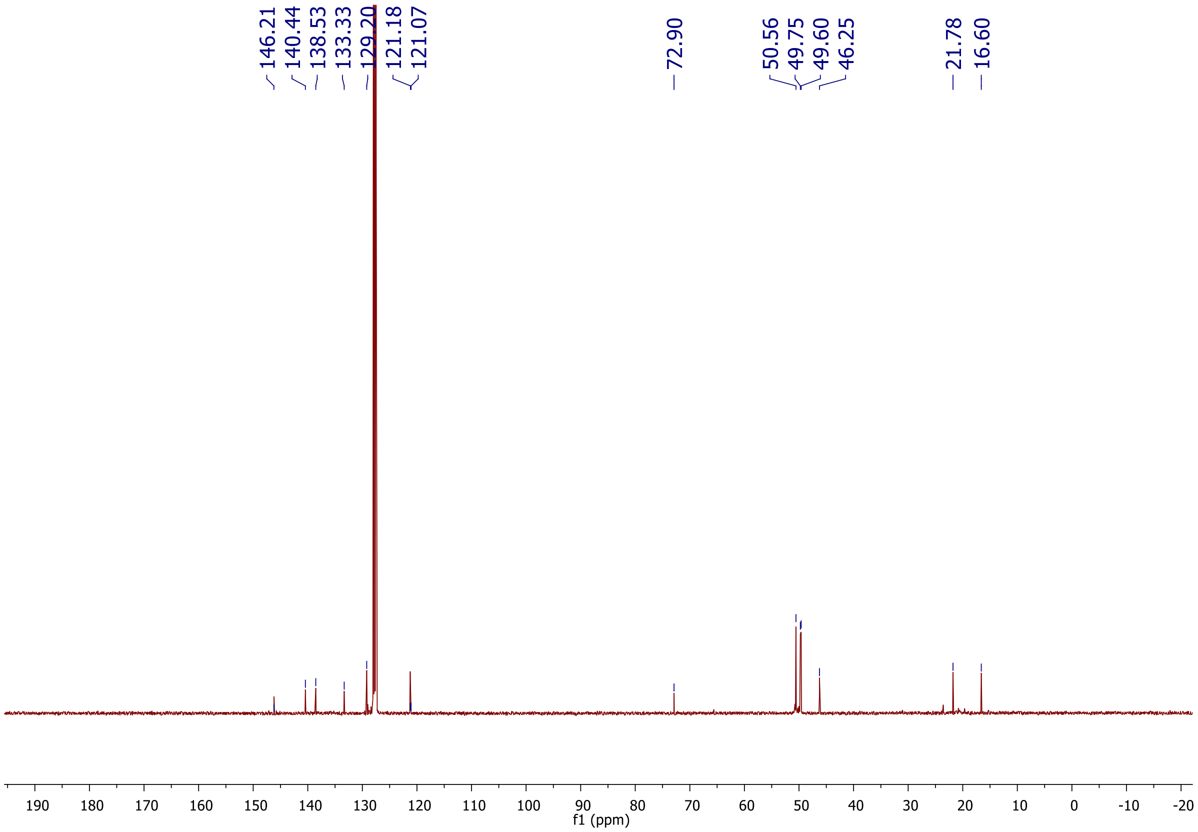

Figure S33: ^{1}H-NMR spectrum of **9b** after irradiation at 405 nm (C_6D_6 solution, vs ext. TMS, ppm)

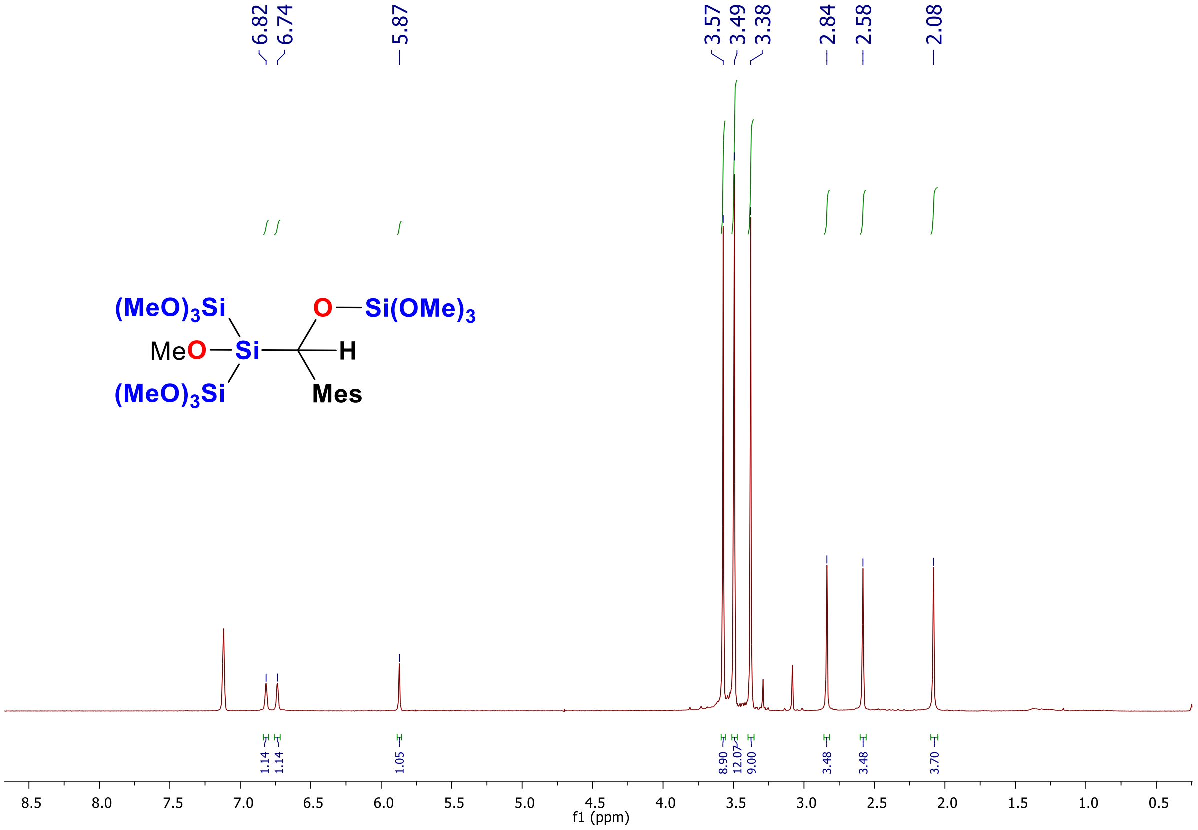

Figure S34: ^{29}Si-INEPT-NMR spectrum **9b** after irradiation at 405 nm (C_6D_6 solution, vs ext. TMS, ppm)

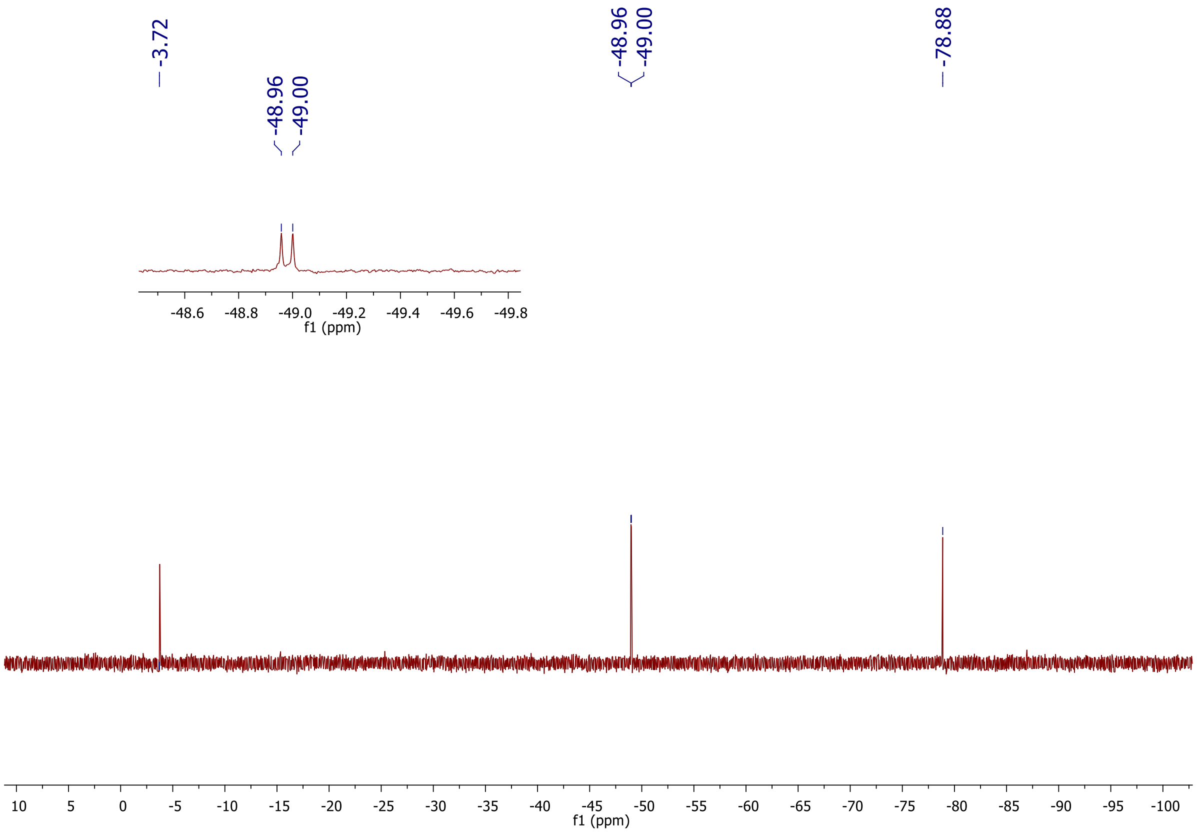

Figure S35: ^{13}C-NMR spectrum of **9b** after irradiation at 405 nm (C_6D_6 solution, vs ext. TMS, ppm)

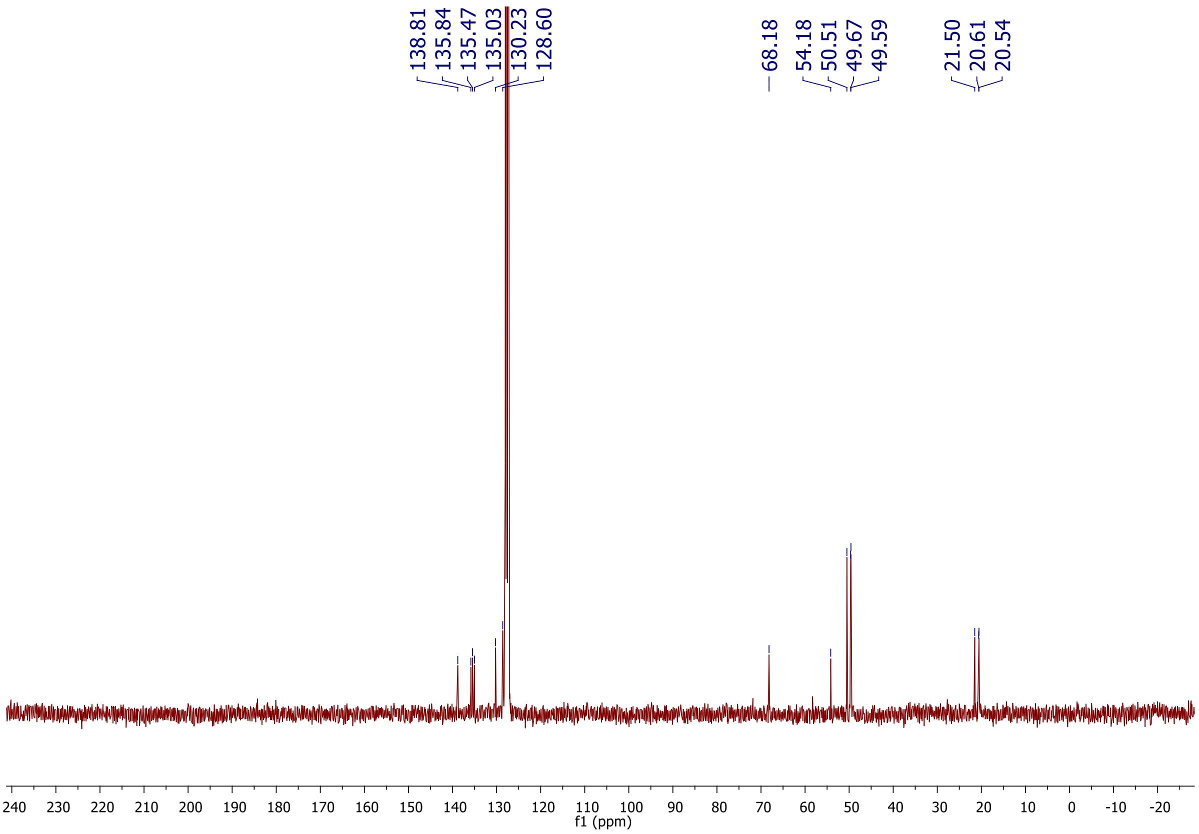

Figure S36: ^{1}H-NMR spectrum of **11** (C_6D_6 solution, vs ext. TMS, ppm)

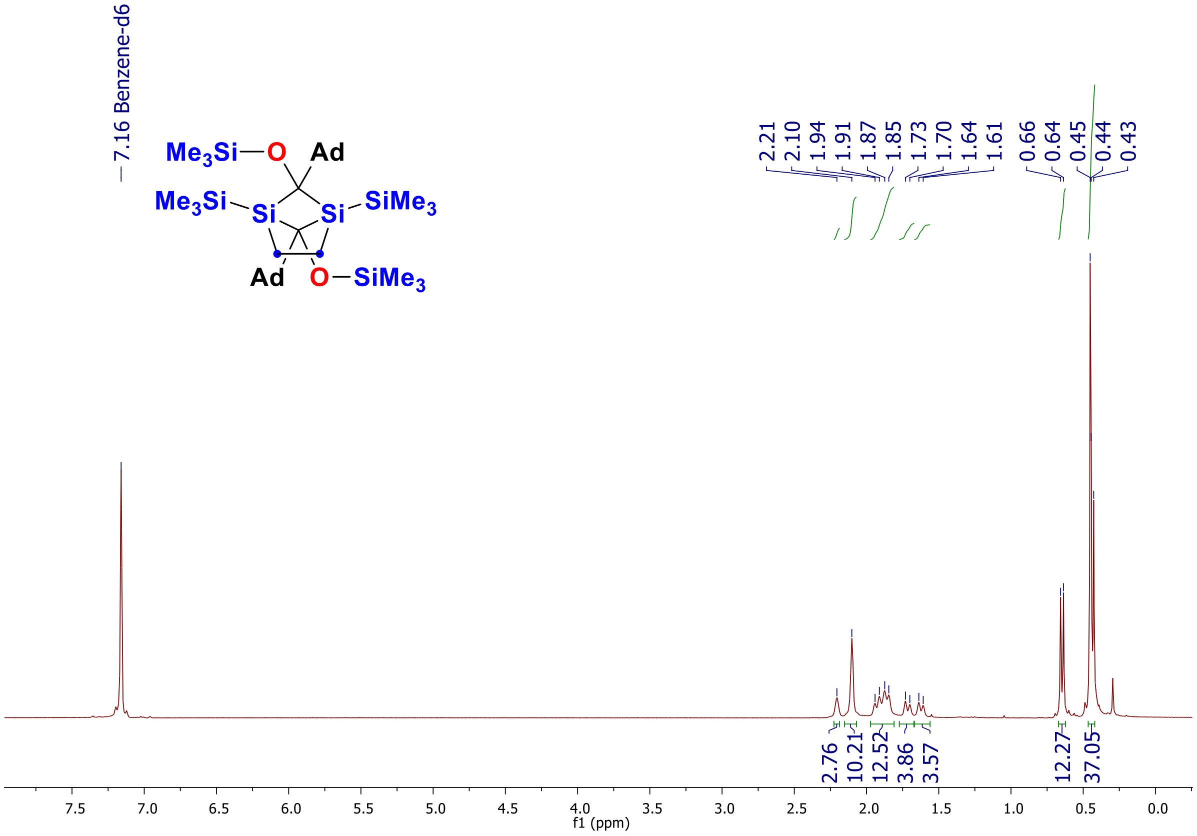

Figure S37: ^{29}Si-INEPT-NMR spectrum of **11** (C_6D_6 solution, vs ext. TMS, ppm)

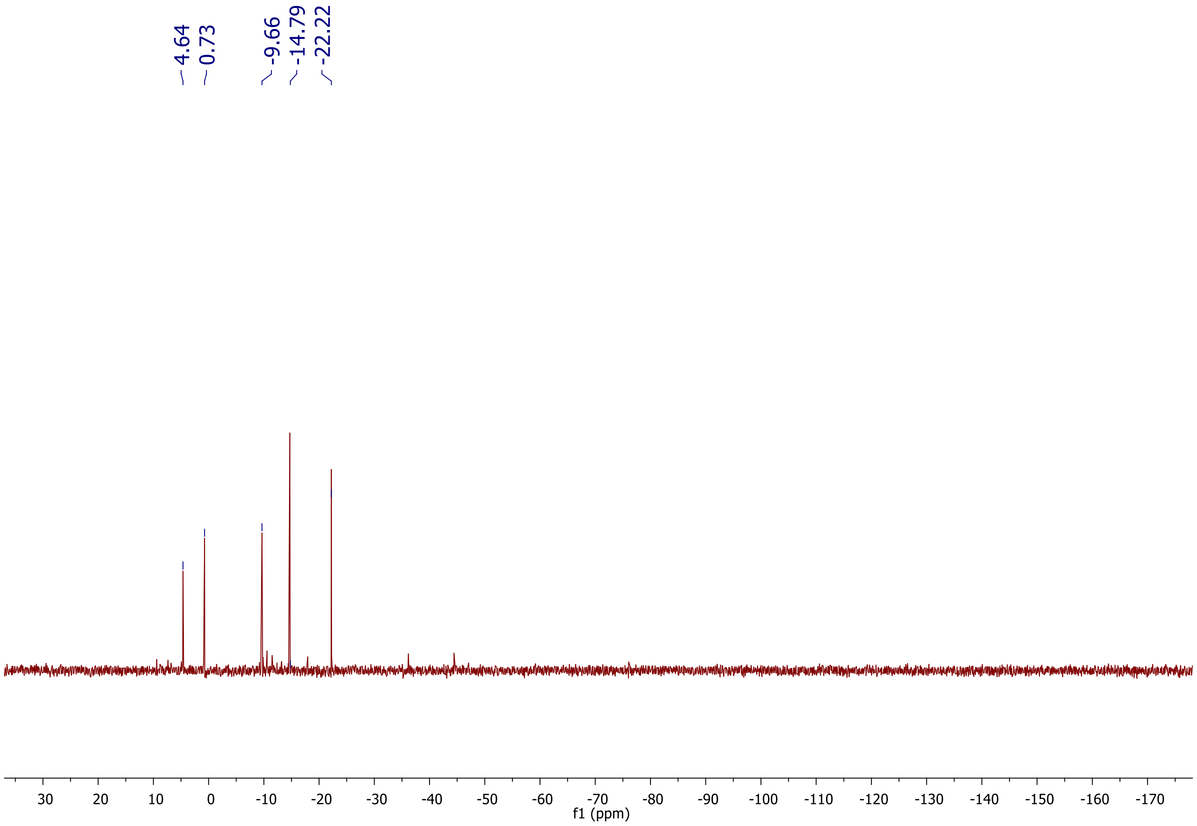

Figure S38: ^{13}C-NMR spectrum of **11** (C_6D_6 solution, vs ext. TMS, ppm)

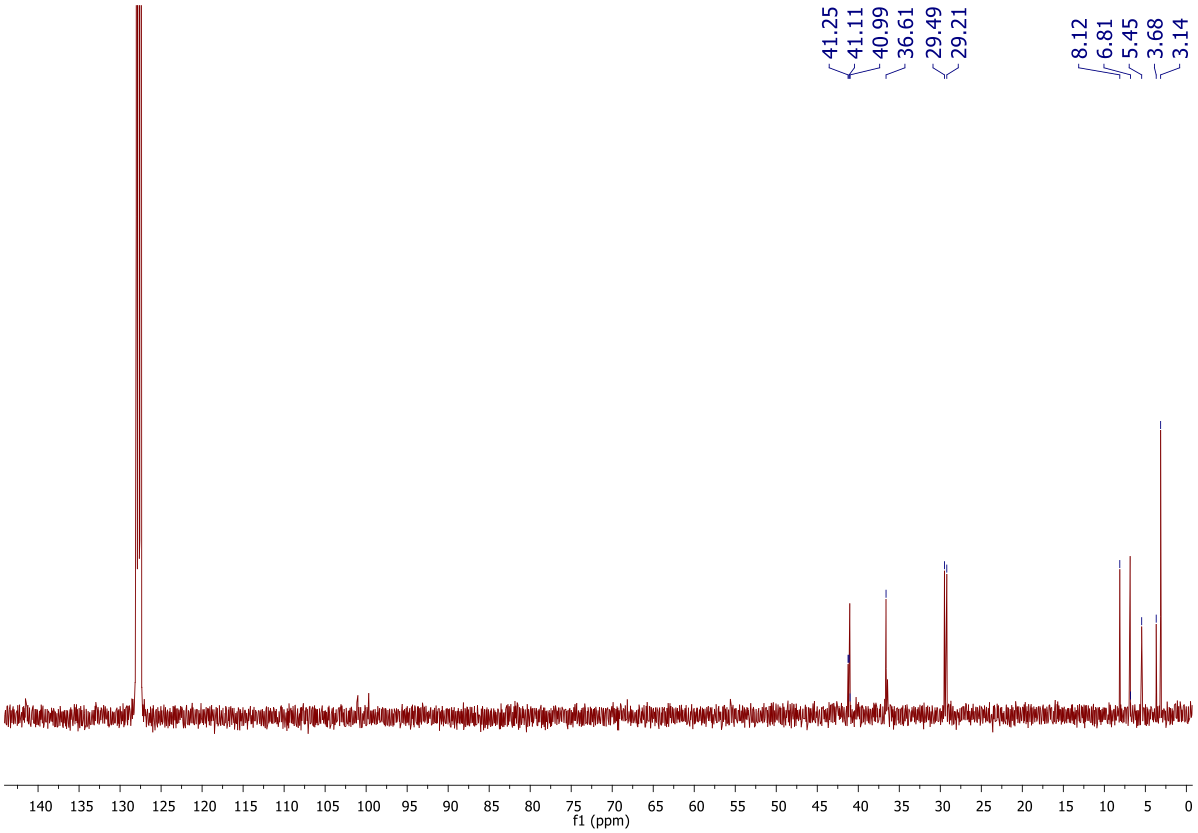

Figure S39: ^{1}H-NMR spectrum of **12** (C_6D_6 solution, vs ext. TMS, ppm)

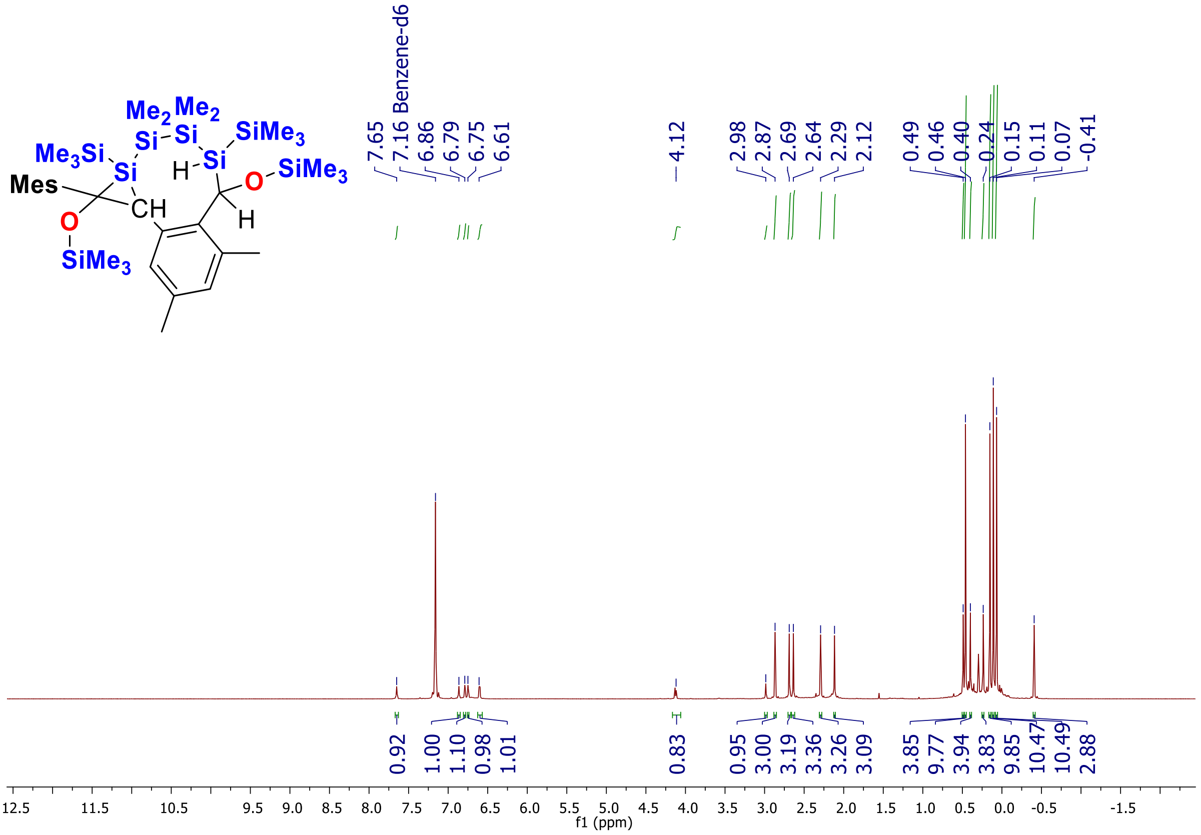

Figure S40: ^{29}Si-INEPT-NMR spectrum of **12** (C_6D_6 solution, vs ext. TMS, ppm)

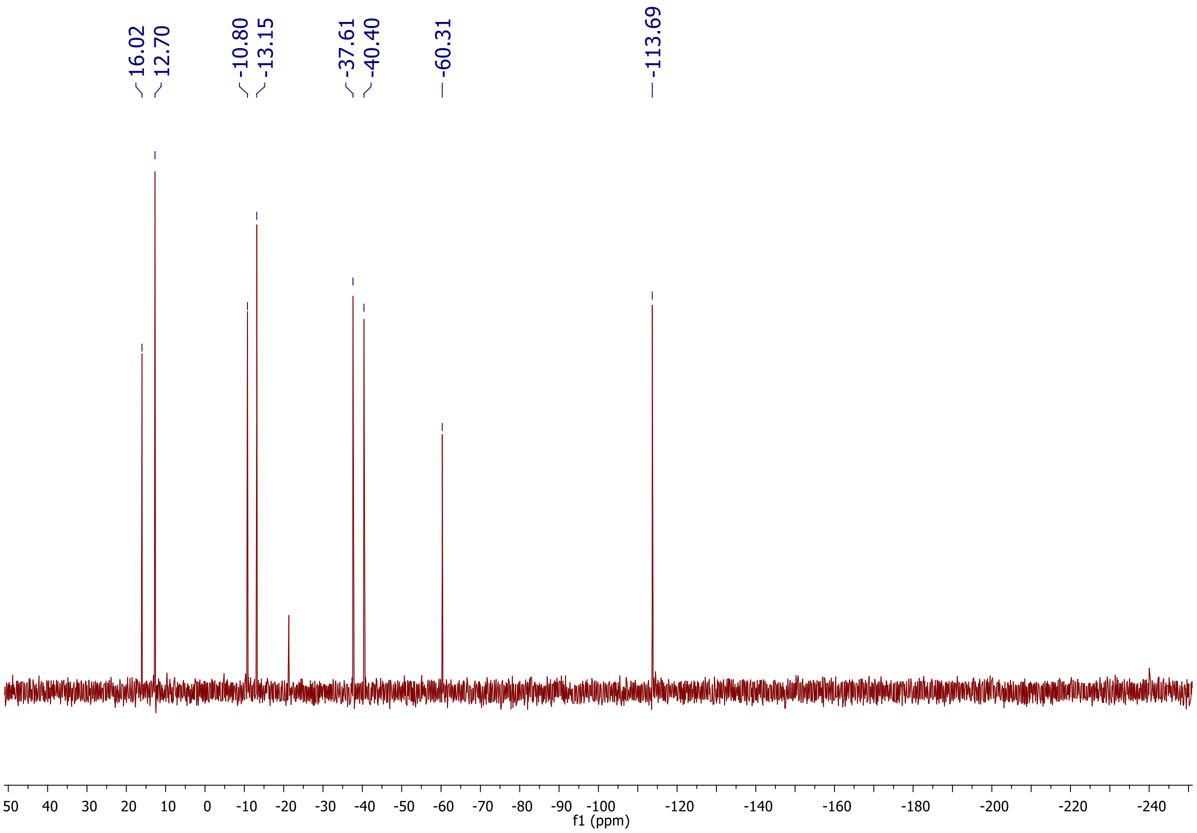

Figure S41: ^{13}C-NMR spectrum of **12** (C_6D_6 solution, vs ext. TMS, ppm)

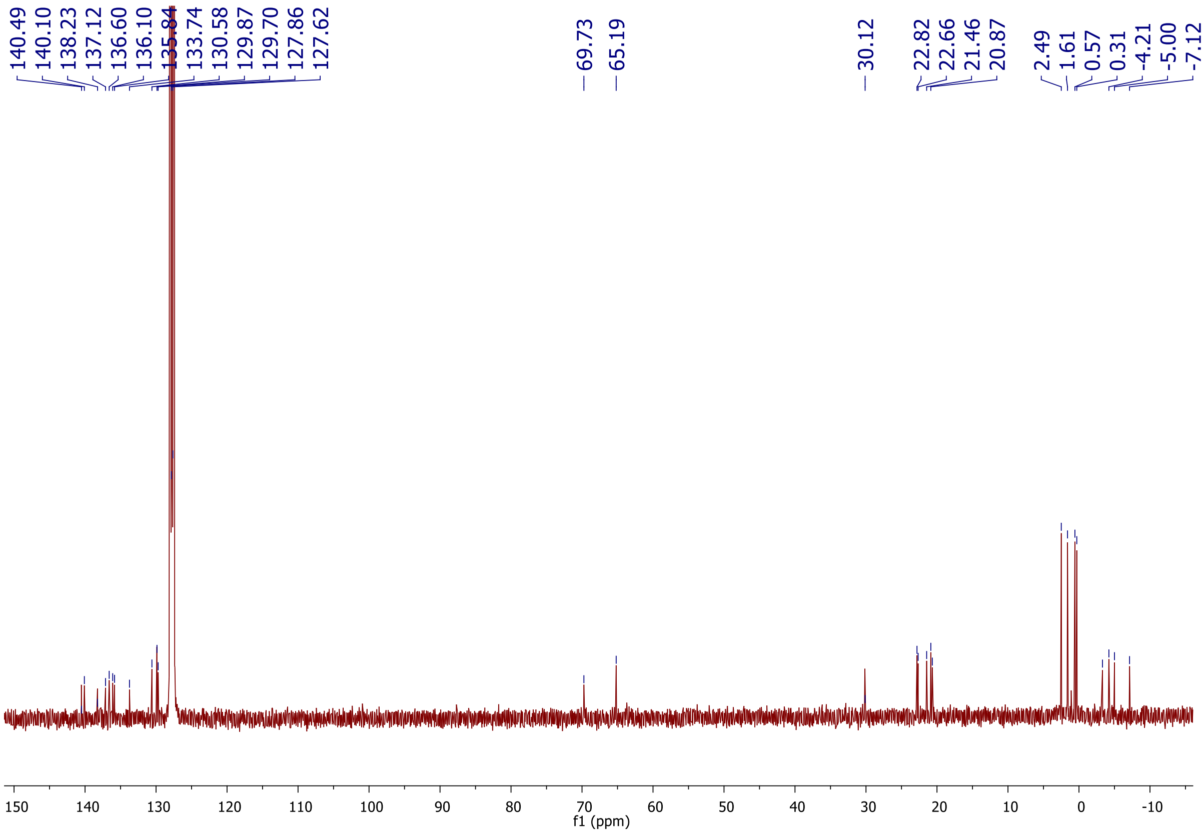

X-ray Crystallography

Table S1. Crystallographic data and details of measurements for compounds **3**, **4**, **7b, 11** and **12**.

**Compound**	**2314683 (3)**	**2314681 (4)**	**2314684 (7b)**	**2314680 (11)**
**Formula**	$C_{38}H_{78}O_2Si_8$	$C_{36}H_{70}O_2Si_8$	$C_{20}H_{42}O_{10}Si_8$	$C_{38}H_{78}O_2Si_8$
**Fw (g mol^{-1})**	791.72	759.64	554.89	791.72
a **(Å)**	11.7402(3)	13.7893(6)	11.4786(4)	11.7946(11)
b **(Å)**	11.7402(3)	15.0403(6)	13.6142(5)	12.9849(10)
c **(Å)**	34.2892(11)	11.4456(5)	18.0049(6)	14.6301(13)
α **(°)**	90	90	85.729(2)	91.151(4)
β **(°)**	90	105.935(2)	79.419(2)	91.077(5)
γ **(°)**	90	90	77.427(2)	90.358(4)
V **(Å^3)**	4726.2(2)	2282.55(17)	2697.75(17)	2239.7(3)
Z	4	2	4	4
**Crystal size (mm)**	0.32 x 0.27 x 0.19	0.31 x 0.15 x 0.09	0.2 x 0.17 x 0.14	
**Crystal habit**	Block, colourless	Block, colourless	Block, colourless	Block, colourless
**Crystal system**	Tetragonal	Monoclinic	Triclinic	Triclinic
**Space group**	*P4(3)2(1)2*	$P2_{1/c}$	*P-1*	*P1*
**d_{calc} (Mg m^{-3})**	1.113	1.105	1.366	1.174
μ **(mm^{-1})**	0.26	0.26	0.27	0.27
T **(K)**	100(2)	100	100.04	100(2)
**2*θ* range (°)**	2.5-29.5	2.5-27.1	2.6-30.1	
***F*(000)**	1736	828	1192	868
**R_{int}**	0.048	0.046	0.046	0.178
**No. of measured and independent [*I* > 2s(*I*)] reflections**	129601, 6819, 6337	63586, 4020, 3467	2213105, 15858, 13860	67257, 16452, 10895
**No. of parameters, restraints**	225, 0	219, 61	631, 0	897, 3
**Δ›$_{max}$, Δ›$_{min}$ (e Å^{-3})**		1.00, -0.25		
**R1, wR2 (all data)**	0.030, 0.061	0.043, 0.088	0.041, 0.089	0.166, 0.232
**R1, wR2 (>2*σ*)**	0.025, 0.066	0.035, 0.094	0.034, 0.094	0.114, 0.254

**Compound**	**2314682 (12)**
**Formula**	$C_{36}H_{70}O_2Si_8$
**Fw (g mol^{-1})**	759.64
a **(Å)**	19.9221(9)
b **(Å)**	11.1419(4)
c **(Å)**	22.8343(10)
α **(°)**	90
β **(°)**	112.601(2)
γ **(°)**	90
V **(Å^3)**	4679.3(3)
Z	4
**Crystal size (mm)**	0.12 x 0.08 x 0.07
**Crystal habit**	Block, colourless

**Crystal system**	Monoclinic
**Space group**	*P*2$_{1/c}$
d_{calc} **(Mg m^{-3})**	1.078
μ **(mm^{-1})**	0.26
T **(K)**	100
**2θ range (°)**	2.2-33.2
***F*(000)**	1656
R_{int}	0.119
**No. of measured and independent [*I* > 2s(*I*)] reflections**	27048, 7991, 4719
**No. of parameters, restraints**	440, 0
**Δ›$_{max}$, Δ›$_{min}$ (e Å^{-3})**	0.53, -0.46
**R1, wR2 (all data)**	0.135, 0.142
**R1, wR2 (>2σ)**	0.063, 0.197

DFT calculations

UV spectras simulation and frontier orbitals involved in highest excitation

The most important features of the computed spectra are well comparable to the recorded spectra. The S1 band is in all cases quite weak . The largest contribution of this excitation usually comes from an molecular orbital consisting of a combination of σ(Si-$SiMe_3$) and oxygen lone pair n. Interestingly, in case of compounds **1**, **5**, and **6** conformers up to 10 kJ/mol higher in energy, denoted as **1b**, **5b**, and **6b**, exhibit a red-shifted S1 band compared to the respective global minimum.

Table S2. Most important UV vertical excitation of compounds **1- 6** (ΔG in kJ/mol): wavelength (in nm), oscillator strength (italic, in brackets) and respective orbital contributions (in brackets). H denotes HOMO, L denotes LUMO.

Compound/ conformer	Wave length (*osc. strength*)	Orbitals
**1** (0.0 kJ/mol)	359 (*0.0025*)	H→L (0.70)
**1b** (7.4 kJ/mol)	384 (*0.0013*)	H→L (0.70)
**2**	384 (*0.0014*)	H→L (0.68)
**3**	386 (*0.0029*)	H-1→L (0.65)
	383 (*0.0031*)	H→L+1(0.65)
**4**	396 (*0.0040*)	H-2→L+1(0.47), H-1→L (0.45)
**5** (0.0 kJ/mol)	376 (*0.0026*)	H→L (0.40), H→L+1 (0.35)
	376 (*0.0018*)	H-1→L (0.40), H→L+1 (0.35)
**5b** (4.2 kJ/mol)	386 (*0.0016*)	H→L (0.54), H→L+1 (0.44)
	370 (*0.0033*)	H-1→L+1 (0.49)
**6** (0.0 kJ/mol)	386 (*0.0021*)	H-2→L (0.56)

	384 (*0.0032*)	H-1$\rightarrow$L+1 (0.51)
**6b** (7.6 kJ/mol)	392 (*0.0020*)	H-2$\rightarrow$L (0.57)
	381 (*0.0030*)	H-1$\rightarrow$L+1 (0.56)

Figure S42 Molecular orbitals involved in S1 excitation for compounds 3 to 6, drawn with contour values of 0.02 a.u.

3

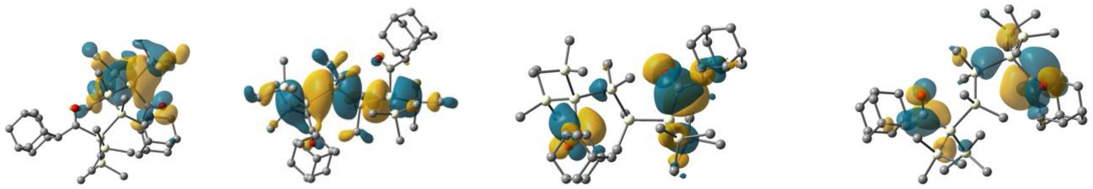

HOMO-1 HOMO LUMO LUMO+1

4

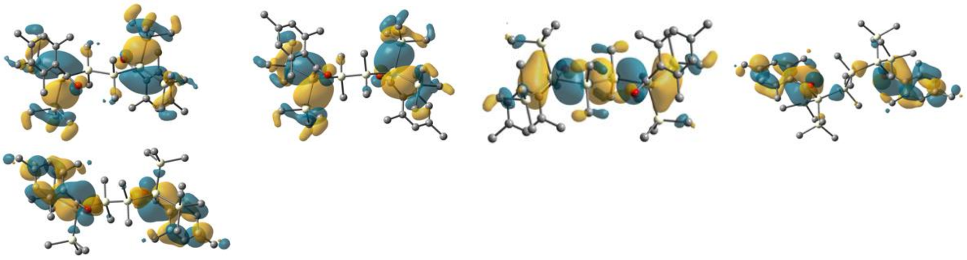

HOMO-2 HOMO-1 HOMO LUMO LUMO+1

5

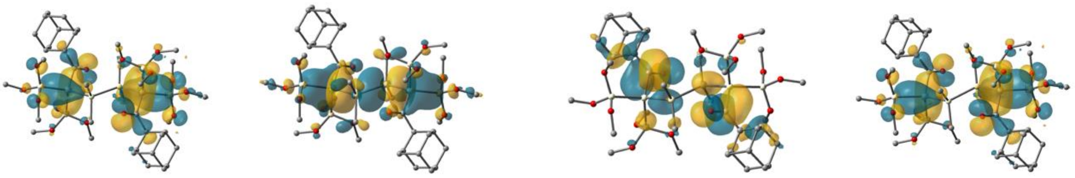

HOMO-1 HOMO LUMO LUMO+1

6

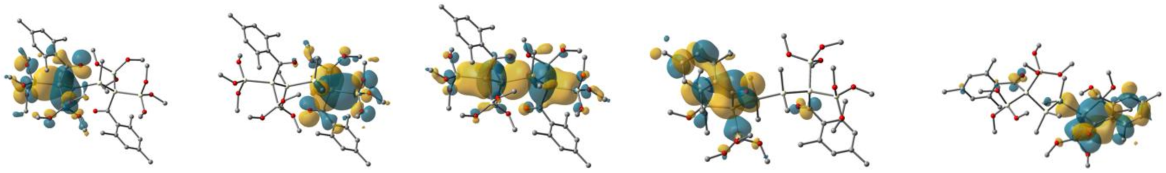

HOMO-2 HOMO-1 HOMO LUMO LUMO+1
